# Supplementary material for: Newly produced synaptic vesicle proteins are preferentially used in synaptic transmission
Source: EMBO J. 2018 Jun 27;37(15):e98044. doi: 10.15252/embj.201798044 (PMC6068464; doi:10.15252/embj.201798044)
Supplement: Supplementary file 1 — Appendix [file EMBJ-37-e98044-s001.pdf]

APPENDIX

to

Title: Newly produced synaptic vesicle proteins are preferentially used in synaptic transmission

Authors: Sven Truckenbrodt[1,2,*,‡], Abhiyan Viplav[1,3†], Sebastian Jähne[1,4], Angela Vogts[5], Annette Denker[1], Hanna Wildhagen[1], Eugenio F. Fornasiero[1,*], and Silvio O. Rizzoli[1,*]

Affiliations:
[1]Institute for Neuro- and Sensory Physiology & center for Biostructural Imaging of Neurodegeneration (BIN), University Medical Center Göttingen, , Göttingen, Germany
[2]International Max Planck Research School for Molecular Biology, Göttingen, Germany
[3]Master Molecular Biology Programme, University of Vienna, Austria
[4]International Max Planck Research School for Neurosciences, Göttingen, Germany
[5]Leibniz Institute for Baltic Sea Research, Warnemünde, Germany
*Correspondence to: Sven Truckenbrodt (strucke@gwdg.de), Eugenio F. Fornasiero (efornas@gwdg.de), Silvio O. Rizzoli (srizzol@gwdg.de)
[†]Present address: Cells in Motion Cluster of Excellence at the University of Münster, Germany
[‡]Present address: IST Austria, Am Campus 1, 3400 Klosterneuburg, Austria

TABLE OF CONTENTS

APPENDIX FIGURES 4

Appendix Figure S1: Co-clusters of synaptic vesicle proteins (Synaptotagmin 1, Synaptophysin) are revealed by Synaptotagmin 1 antibodies on the plasma membrane after exocytosis. 4

Appendix Figure S2: The Synaptotagmin 1 antibody labeling is specific, since non-specific antibodies are not taken up. 5

Appendix Figure S3: The Synaptotagmin 1 antibodies remain bound to their targets for long time periods. 7

Appendix Figure S4: Virtually all Synaptotagmin 1 antibodies bound to their targets are exocytosed upon 1200 AP stimulation. 8

Appendix Figure S5: A one-hour incubation with Synaptotagmin 1 antibodies saturates the available epitopes. 9

Appendix Figure S6: The Synaptotagmin 1 antibody used for live-tagging co-localized well with Synaptophysin, but not with Chromogranin A, Rab5 or Rab7. 11

Appendix Figure S7: An analysis of the fraction of Synaptotagmin 1 molecules labeled by the antibodies. 12

Appendix Figure S8: The Synaptotagmin 1 antibodies remain in synapses for long time periods (days). 14

Appendix Figure S9: The Synaptotagmin 1 antibodies appear in lysosomes in the neuronal cell body after a few days of incubation. 16

Appendix Figure S10: Neurons of different ages show similar levels of synaptic activity. 17

Appendix Figure S11: Neurons of different ages have similar densities of synaptic boutons, as well as similar bouton sizes and vesicle amounts. 20

Appendix Figure S12: Recycling Synaptotagmin 1 proteins become inactive within less than a day during spontaneous network activity. 21

Appendix Figure S13: Young overexpressed VAMP2 molecules are released preferentially. 23

Appendix Figure S14: Quantification of the effects of bicuculline or 8 mM Ca^{2+} on neuronal activity. 26

Appendix Figure S15: An analysis of the synaptic vesicle recycling under spontaneous network activity. 27

Appendix Figure S16: Additional experiments confirm that ageing Synaptotagmin 1 molecules co-localize with SNAP25, but not with Syntaxin 1. 30

Appendix Figure S17: The co-localization of SNAP25 with Synaptotagmin 1 is high for aged Synaptotagmin 1 molecules. 32

Appendix Figure S18: SypHy, sypHy-SNAP25, and sypHy-Syntaxin 1 show similar patterns of expression in hippocampal neurons. 33

Appendix Figure S19: Targeting Syntaxin 1 to synaptic vesicles increases the amount of SNAP25 in synaptic boutons. 34

Appendix Figure S20: The expression of SNAP25 in neurons does not affect synaptic bouton morphology or synaptic vesicle amounts. 36

Appendix Figure S21: The expression of Syntaxin 1 in neurons does not affect synaptic bouton morphology or synaptic vesicle amounts. 38

Appendix Figure S22: The expression of CSPα$_{WT}$ in neurons does not affect synaptic bouton morphology or synaptic vesicle amounts. 40

Appendix Figure S23: The expression of CSPα$_{mut}$ in neurons does not affect synaptic bouton morphology or synaptic vesicle amounts. 42

Appendix Figure S24: The expression of CSPα$_{WT}$ in neurons, along with SNAP25, does not affect synaptic bouton morphology or synaptic vesicle amounts. 44

Appendix Figure S25: The expression of CSPα$_{mut}$ in neurons, along with SNAP25, does not affect synaptic bouton morphology or synaptic vesicle amounts. 46

Appendix Figure S26: Vesicles containing sypHy-SNAP25 are efficient in exocytosis upon the first few stimuli of a train. 48

Appendix Figure S27: CSPα overexpression results in neurite degeneration. 50

Appendix Figure S28: Overexpression of CSPα does not alter the Ca^{2+} signaling of the neurons. 52

Appendix Figure S29: Aged synaptic vesicle proteins appear to be poorly endocytosed. 53

Appendix Figure S30: SNAP25 overexpression targets synaptic vesicles to recycling endosomes. 55

Appendix Figure S31: Synaptic vesicle protein entry in the inactive pool precedes the accumulation of damaged synaptic vesicle proteins, as predicted from their lifetimes. 56

MATERIAL AND METHODS 57

APPENDIX REFERNCES 63

APPENDIX FIGURES

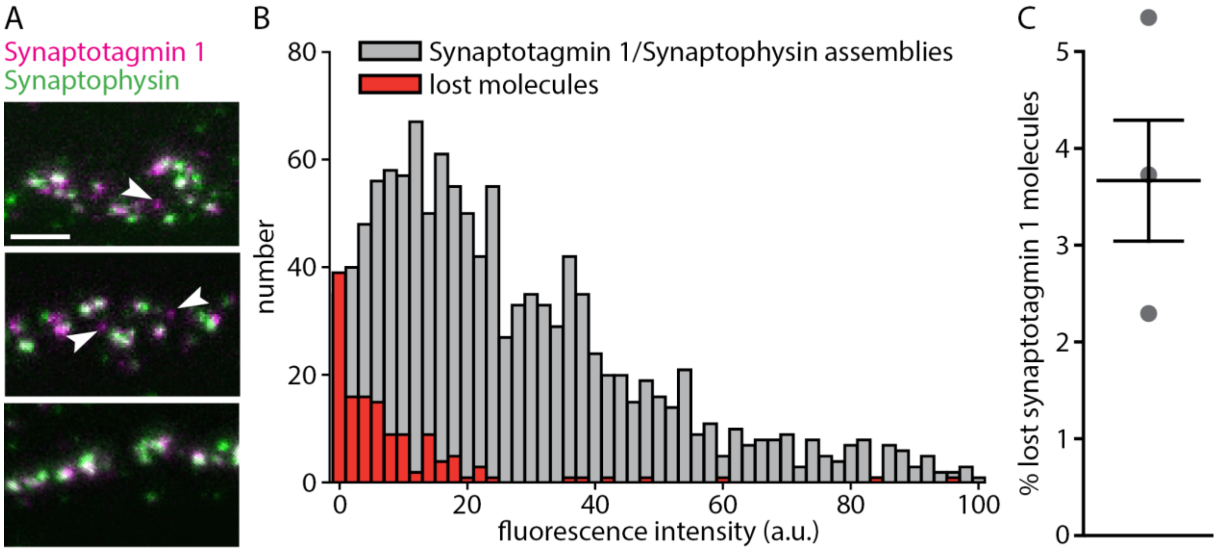

Appendix Figure S1: Co-clusters of synaptic vesicle proteins (Synaptotagmin 1, Synaptophysin) are revealed by Synaptotagmin 1 antibodies on the plasma membrane after exocytosis.

(A) Living hippocampal neurons were mildly stimulated by depolarizing for 6 minutes with 15 mM KCl, in presence of Atto647N-conjugated antibodies directed against the lumenal domains of Synaptotagmin 1. To ensure that the antibodies only revealed Synaptotagmin 1 molecules that were exocytosed during stimulation, and not epitopes that were already present on the plasma membrane, we had blocked these epitopes by incubating the neurons with non-fluorophore-conjugated antibodies (Hoopmann et al., 2010). The neurons were then fixed with ice-cold fixative (PFA solutions), to prevent endocytosis or movement of molecules during fixation, and they were immunostained for another synaptic vesicle marker, Synaptophysin, using an antibody that recognized the lumenal domain of Synaptophysin (Hoopmann et al., 2010). The immunostaining was performed without permeabilization, to dectect only surface Synaptophysin molecules. The neurons were then imaged by 2-color STED microscopy. The arrowheads point to Synaptotagmin 1 molecules (Atto647N signals) that do not co-localize with Synaptophysin, and therefore are considered to be lost from the meta-stable vesicle protein assemblies after exocytosis. Scale bar: 500 nm. We would like to point out that these make only a small fraction of all Synaptotagmin 1 molecules.

(B) We quantified the intensity of the Synaptotagmin 1 spots, in arbitrary units, separating them in spots that co-localized with Synaptophysin (gray) or spots that did not, and thus presumably correspond to “lost” Synaptotagmin 1 molecules (red) and/or to antibodies bound non-specifically to the plasma membrane. n = 1219 vesicles and 136 lost Synaptotagmin 1 molecules, from 3 independent experiments.

(C) Quantification of the amount of “lost” Synaptotagmin 1 molecules, as percent of all exocytosed Synaptotagmin 1 (same set of experiments as in panel (B)). The total amount of “lost” molecules is expressed here as percentage of the entire Synaptotagmin 1 intensity. All data represent the mean ± SEM.

Data information: All imaging was performed on a two-color STED microscope (as described in Hoopmann et al., 2010). For further information, see source data.

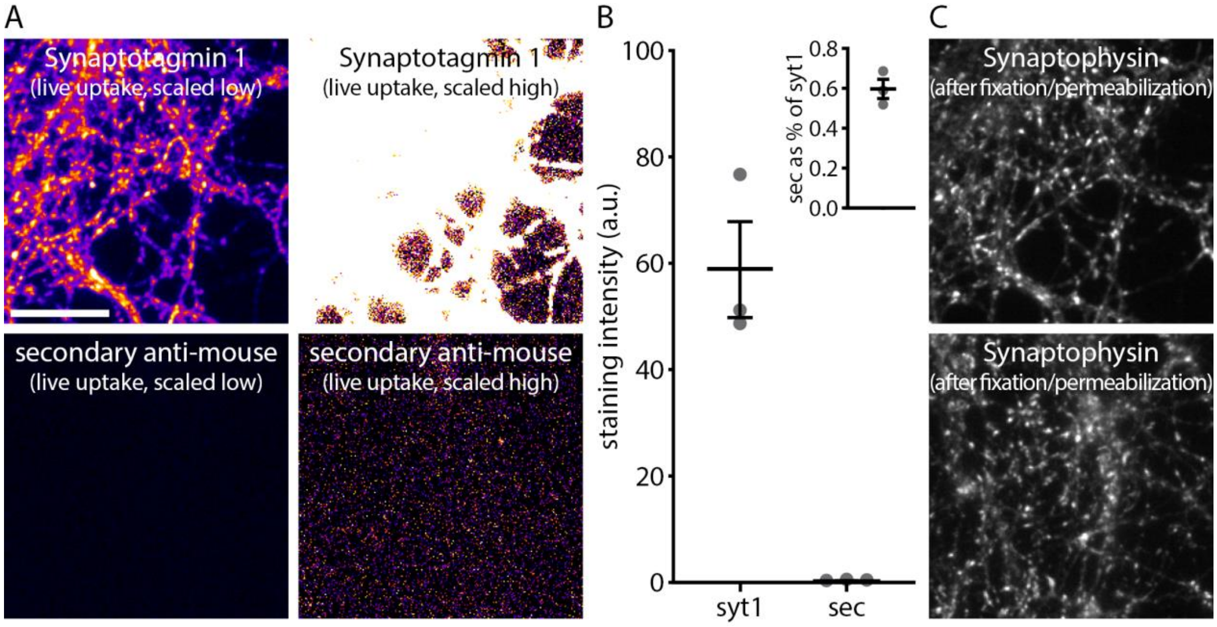

Appendix Figure S2: The Synaptotagmin 1 antibody labeling is specific, since non-specific antibodies are not taken up.

To test whether the 604.2 Synaptotagmin 1 antibody conjugated to Atto647N that we employed is taken up specifically through epitope interaction, as opposed to non-specific endocytic uptake, we incubated living neurons for one hour at 37°C with the 604.2 Synaptotagmin 1 antibody or with an equimolar amount of an anti-mouse IgG secondary antibody conjugated to Atto647N, as a non-specific antibody control.

(A) Typical example of a neuronal culture incubated with the 604.2 Synaptotagmin 1 antibody, or with the secondary antibody. Scale bar: 20 µm.

(B) Quantification of the fluorescence intensity in the two different conditions (syt1 = Synaptotagmin 1; sec = secondary anti-mouse antibody; n = 3). We found that the amount of secondary anti-mouse antibody antibody taken up non-specifically was much lower than that of Synaptotagmin 1 (<1%, see inset). All data represent the mean ± SEM.

(C) To document that neurons were indeed imaged in both experiments, even though they are not visible in the lower panels, an immunostaining for Synaptophysin was performed, after fixation and permeabilization. Synapses are evident in both the upper and lower panels.

Data information: All imaging was performed on a Nikon Ti-E epifluorescence microscope. For further information, see source data.

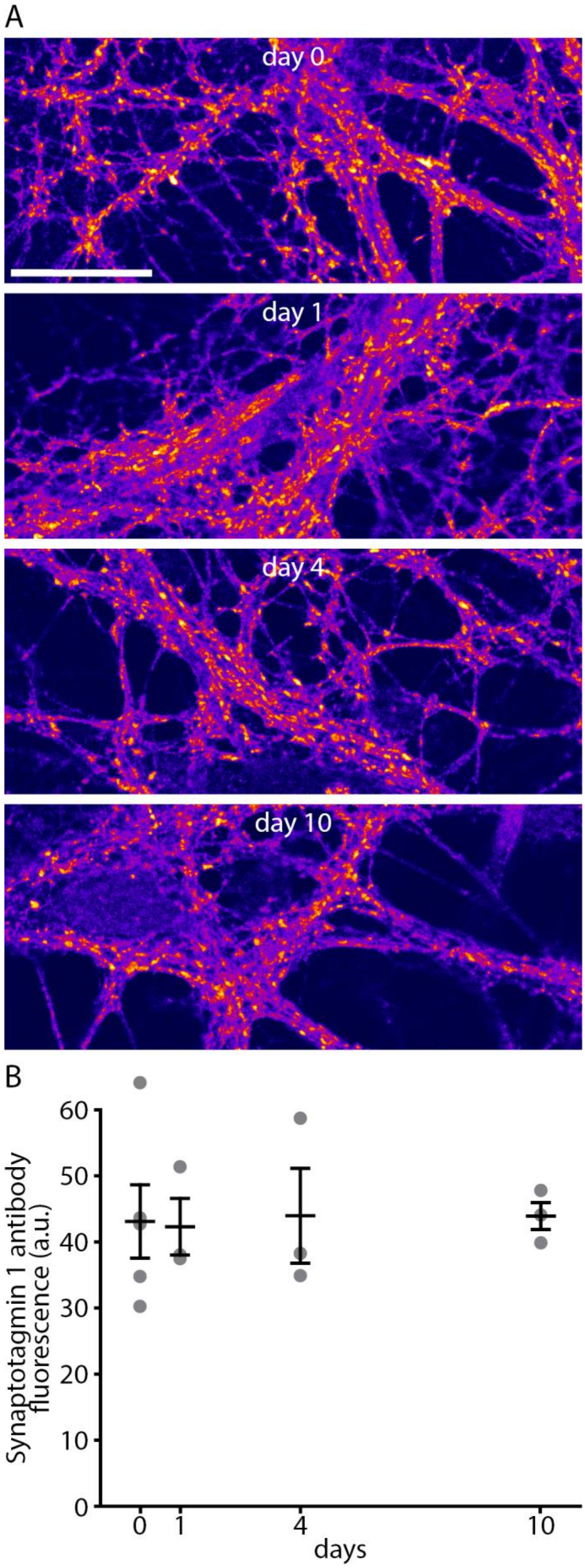
A
day 0
day 1
day 4
day 10
B
Synaptotagmin 1 antibody fluorescence (a.u.)
60
50
40
30
20
10
0
0
1
4
10
days

Appendix Figure S3: The Synaptotagmin 1 antibodies remain bound to their targets for long time periods.

To determine whether the Synaptotagmin 1 antibodies remain bound to their targets for long time periods, we incubated fixed neurons with the Atto647N-conjugated Synaptotagmin 1 antibodies (without permeabilization) for 1 hour, and then placed them in an incubator at 37°C, in a solution containing a 100x molar excess of antigenic peptide. If the antibodies come off their targets, then they become blocked by the antigenic peptide, and can no longer bind the neuronal epitopes. The coverslips were then retrieved at different time points, were washed to remove the antibodies that came off the epitopes, and were imaged to measure the Synaptotagmin 1 fluorescence. The experiments were performed in a pH 5.5 buffer, to mimic the conditions encountered by the antibodies in vesicles in living neurons.

(A) Typical images at different time points after the incubation started. Scale bar: 20 µm.

(B) Quantification of the fluorescence intensity, from confocal imaging as in panel (A), using a Leica confocal microscope. No significant loss of the Synaptotagmin 1 antibody could be observed (n = 5, 3, 3, 3 independent experiments per time point; no statistically significant differences as determined via one-way ANOVA, $p = 0.9945$, $F(3, 13) = 0.02$). All data represent the mean ± SEM.

Date information: All imaging was performed on a Leica SP5 confocal microscope. For further information, see source data.

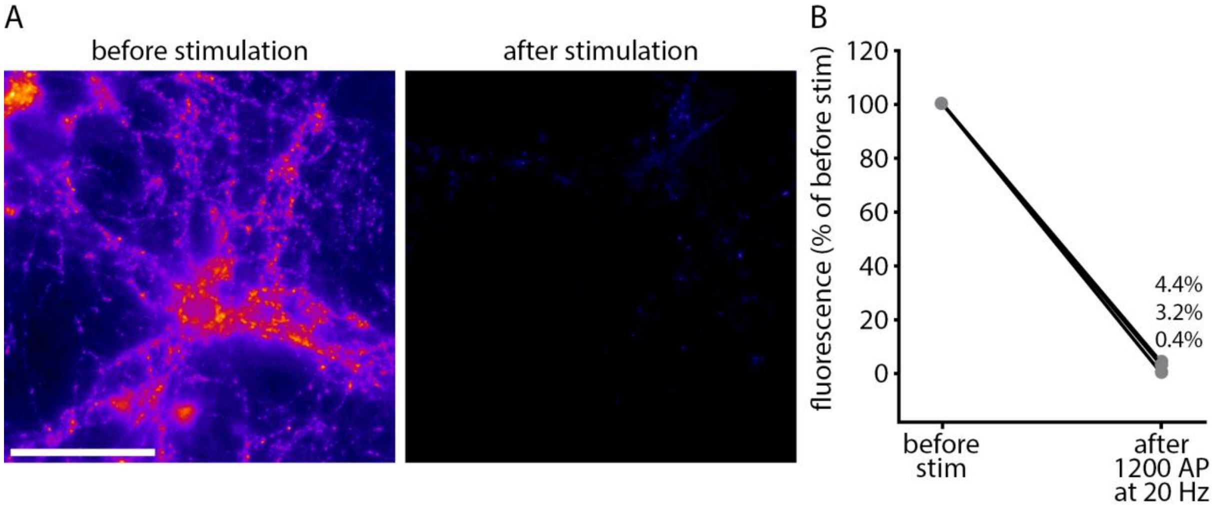

Appendix Figure S4: Virtually all Synaptotagmin 1 antibodies bound to their targets are exocytosed upon 1200 AP stimulation.

To test if the Synaptotagmin 1 antibody tagged predominantly synaptic vesicles, as opposed to, for example, endosomal organelles, we stimulated neurons electrically immediately after 1 h of antibody live-tagging with CypHer5E-conjugated Synaptotagmin 1 antibodies. CypHer5E is pH-sensitive, and is fluorescent within the vesicles, at the 5.5 pH, but is not fluorescent upon exocytosis, when it is exposed to neutral pH. We therefore stimulated the samples electrically, to determine the amount of release. Since endosome release is not readily triggered by electrical stimulation, the amount of antibodies exocytosed indicates the minimal amount of antibodies that is bound to synaptic vesicles.

(A) Left panel: example image of neurites in which the Synaptotagmin 1 epitopes were labeled using CypHer5E-conjugated antibodies. A bright fluorescence is observed, due to antibodies found within acidic (low pH) organelles, such as synaptic vesicles. Scale bar: 50 µm. Right panel: the same field was imaged after stimulation (1200 APs at 20 Hz, in presence of bafilomycin to prevent re-acidification during imaging). The fluorescence is lowered by exocytosis.

(B) We quantified the image fluorescence from images such as those presented here (taken with a Nikon Ti-E epifluorescence microscope). We observed that almost all antibodies were exocytosed, and that they were therefore bound to synaptic vesicles. The values for the remaining fraction of non-responsive (not released) antibodies were 0.4%, 3.2%, and 4.4% in the 3 independent experiments we performed ($p < 0.0001$, $t(2) = 82.1314$). Statistical significance was evaluated using the paired t-test.

Data information: All imaging was performed on a Nikon Ti-E epifluorescence microscope, at 37°C. For further information, see source data.

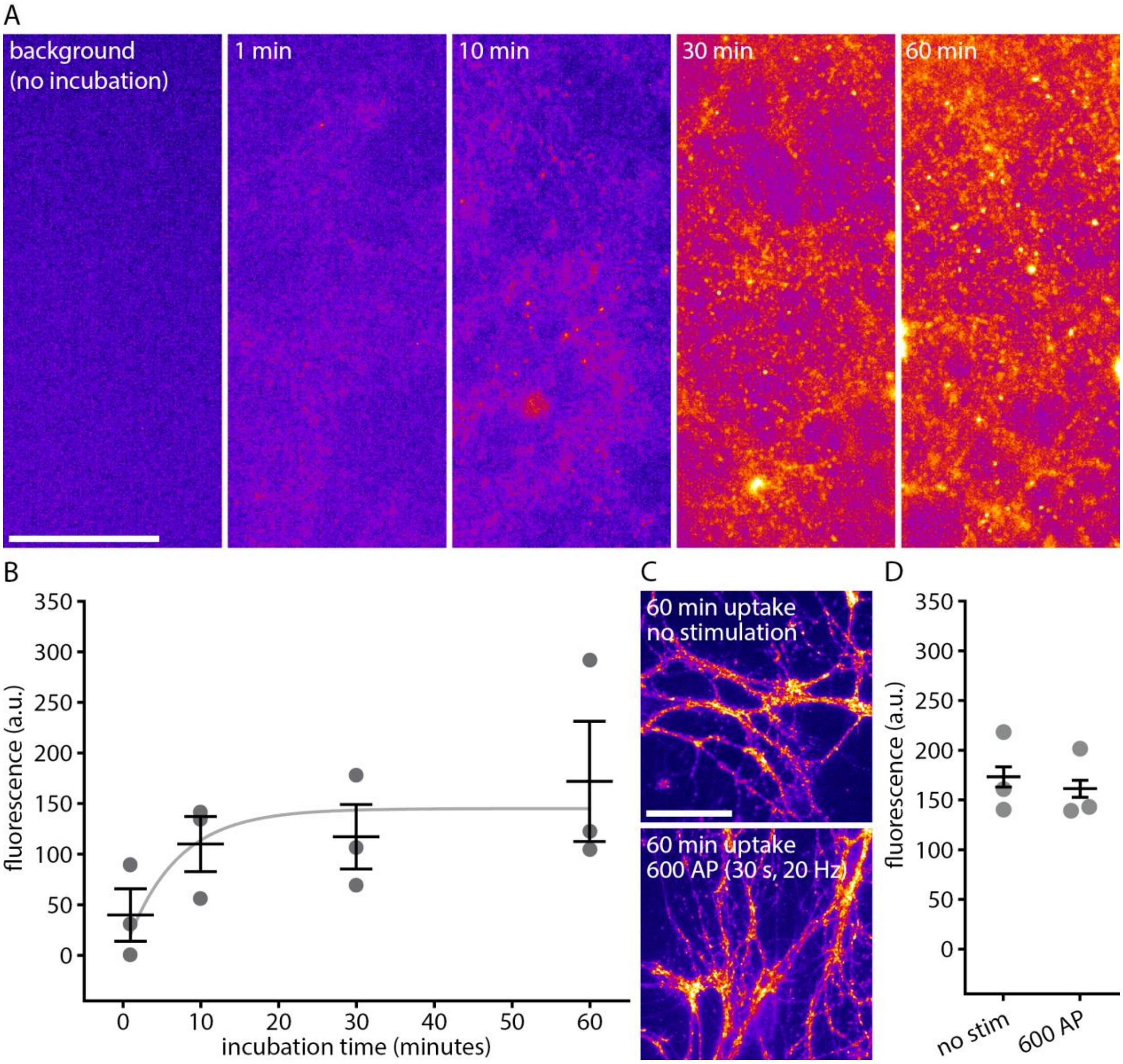

Appendix Figure S5: A one-hour incubation with Synaptotagmin 1 antibodies saturates the available epitopes.

(A) We incubated live neurons with Atto647N-conjugated Synaptotagmin 1 antibodies for different time intervals. Scale bar: 50 µm. All imaging was performed on a Cytation 3 cell imaging device.

(B) We analyzed the fluorescence intensity, and found that the labeling saturates after ~30-60 min of incubation (n = 3 experiments per data point), as indicated by the fit of an exponential rise to maximum curve in light grey (r^2 = 0.80, P < 0.0001).

(C-D) Alternatively, we stimulated the neurons after one hour of incubation, in presence of the antibodies, for 30 seconds at 20 Hz, to release and recycle all vesicles from the recycling pool (bottom). We imaged them using a Nikon Ti-E epifluorescence microscope, and we analyzed the intensity as above. No significant change took place (p = 0.72, t(4) = 0.385). Statistical significance was evaluated using the paired t-test.

Data information: All data represent the mean ± SEM. For further information, see source data.

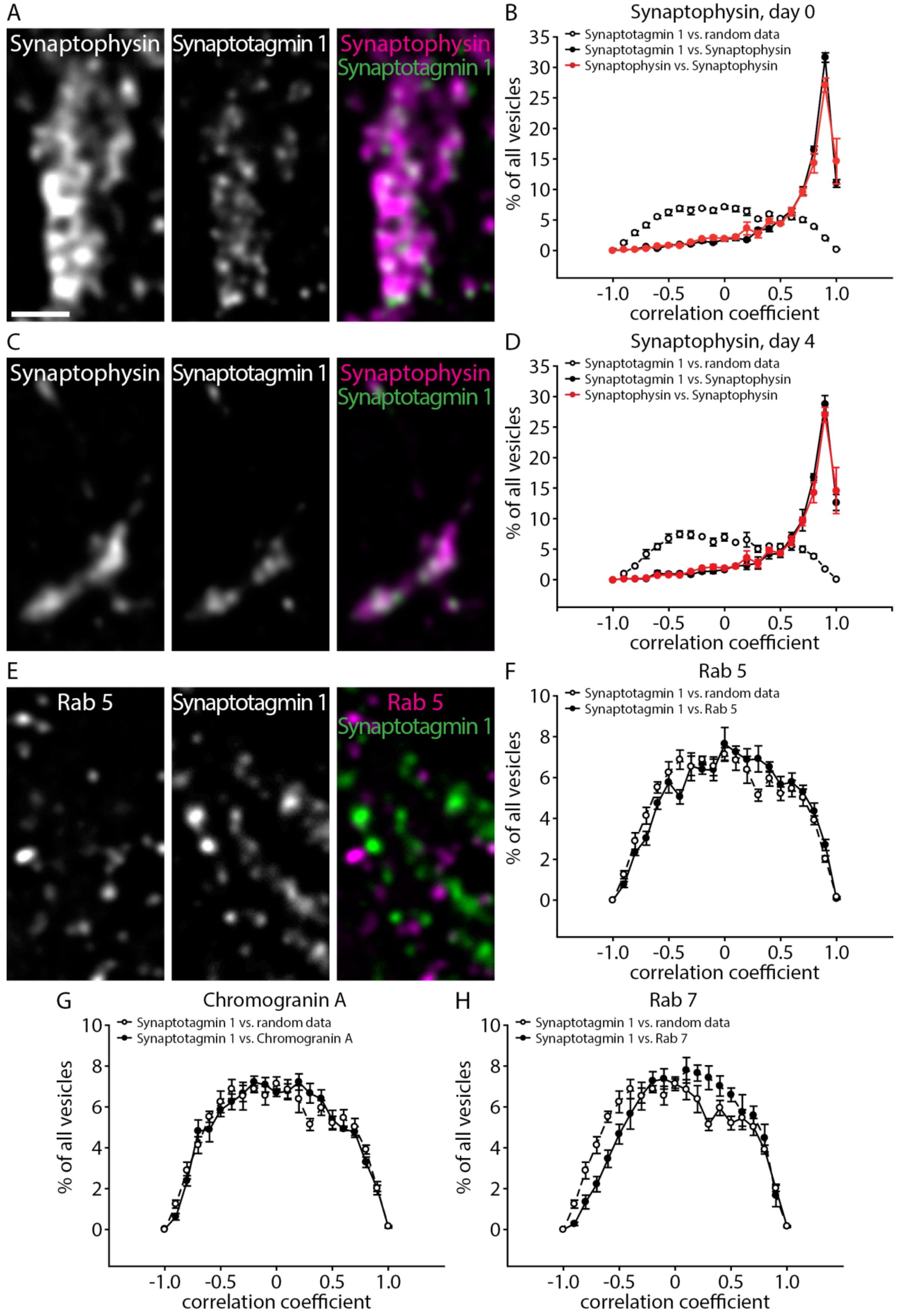
A
Synaptophysin
Synaptotagmin 1
Synaptophysin
Synaptotagmin 1
B
Synaptophysin, day 0
Synaptotagmin 1 vs. random data
Synaptotagmin 1 vs. Synaptophysin
Synaptophysin vs. Synaptophysin
% of all vesicles
correlation coefficient
C
Synaptophysin
Synaptotagmin 1
Synaptophysin
Synaptotagmin 1
D
Synaptophysin, day 4
Synaptotagmin 1 vs. random data
Synaptotagmin 1 vs. Synaptophysin
Synaptophysin vs. Synaptophysin
% of all vesicles
correlation coefficient
E
Rab 5
Synaptotagmin 1
Rab 5
Synaptotagmin 1
F
Rab 5
Synaptotagmin 1 vs. random data
Synaptotagmin 1 vs. Rab 5
% of all vesicles
correlation coefficient
G
Chromogranin A
Synaptotagmin 1 vs. random data
Synaptotagmin 1 vs. Chromogranin A
% of all vesicles
correlation coefficient
H
Rab 7
Synaptotagmin 1 vs. random data
Synaptotagmin 1 vs. Rab 7
% of all vesicles
correlation coefficient

Appendix Figure S6: The Synaptotagmin 1 antibody used for live-tagging co-localized well with Synaptophysin, but not with Chromogranin A, Rab5 or Rab7.

(A,C,E) We incubated primary hippocampal cultures with Atto647N-conjugated lumenal Synaptotagmin 1 antibodies, as in Fig 1. We then fixed and permeabilized them, and performed a co-immunostaining for markers of synaptic vesicles (Synaptophysin, (A-D)), constitutively releasing compartments (Chromogranin A, (C,D)), and endosomal compartments (Rab 5, (E); Rab 7, (F)). Synaptotagmin 1 is shown in green in the exemplary images, with the protein of interest shown in magenta. Scale bar: 500 nm. The Synaptophysin colocalization was tested both under conditions of direct fixation (C) and after a further 4 days of incubation of the neurons at 37°C (E).

(B,D,F,G,H) We then analyzed the co-localization of the Synaptotagmin 1 antibody with the protein of interest immunostaining, by drawing line-scans through each spot of the Synaptotagmin 1 staining, and correlating the respective line scan to an identical line scan drawn in the imaging channel of the protein of interest (using Pearson's correlation coefficient). We typically analyzed 1000-1300 spots in each independent experiment, and we generated histograms of correlation coefficient distributions. Average histograms are shown ($n = 3$ independent experiments per data point). To compare the correlation coefficient distributions to positive and negative controls, we used the following. Positive control: a double immunostaining of Synaptophysin, using one primary antibody and two secondary antibodies conjugated to two different fluorophores (same fluorophores as in the other experiments). Negative control: the same analysis, performed in the Chromogranin A images, after rotating horizontally (mirroring) the green image. The correlation coefficient distribution obtained for the Synaptotagmin 1 antibody with the synaptic vesicle marker Synaptophysin (B,D) was significantly different from the negative control ($p < 0.0001$), but indistinguishable from the positive control ($p > 0.5$). Conversely, the correlation coefficient distributions obtained for the other markers were indistinguishable from the negative control: (D) $p = 0.9999$, $t(20) = 0.0001$; (E) $p = 0.9999$, $t(20) < 0.0001$; (F) $p = 0.9999$, $t(20) < 0.0001$).

All imaging was performed using two-color STED microscopy on an Abberior 3D STED setup. All data represent the mean ± SEM. Statistical significance was evaluated using ANOVA ($p < 0.0001$, $F(2, 12) = 332.86$), followed by Bonferroni procedure (B), or unpaired t-tests (D-F).

Data information: For further information, see source data.

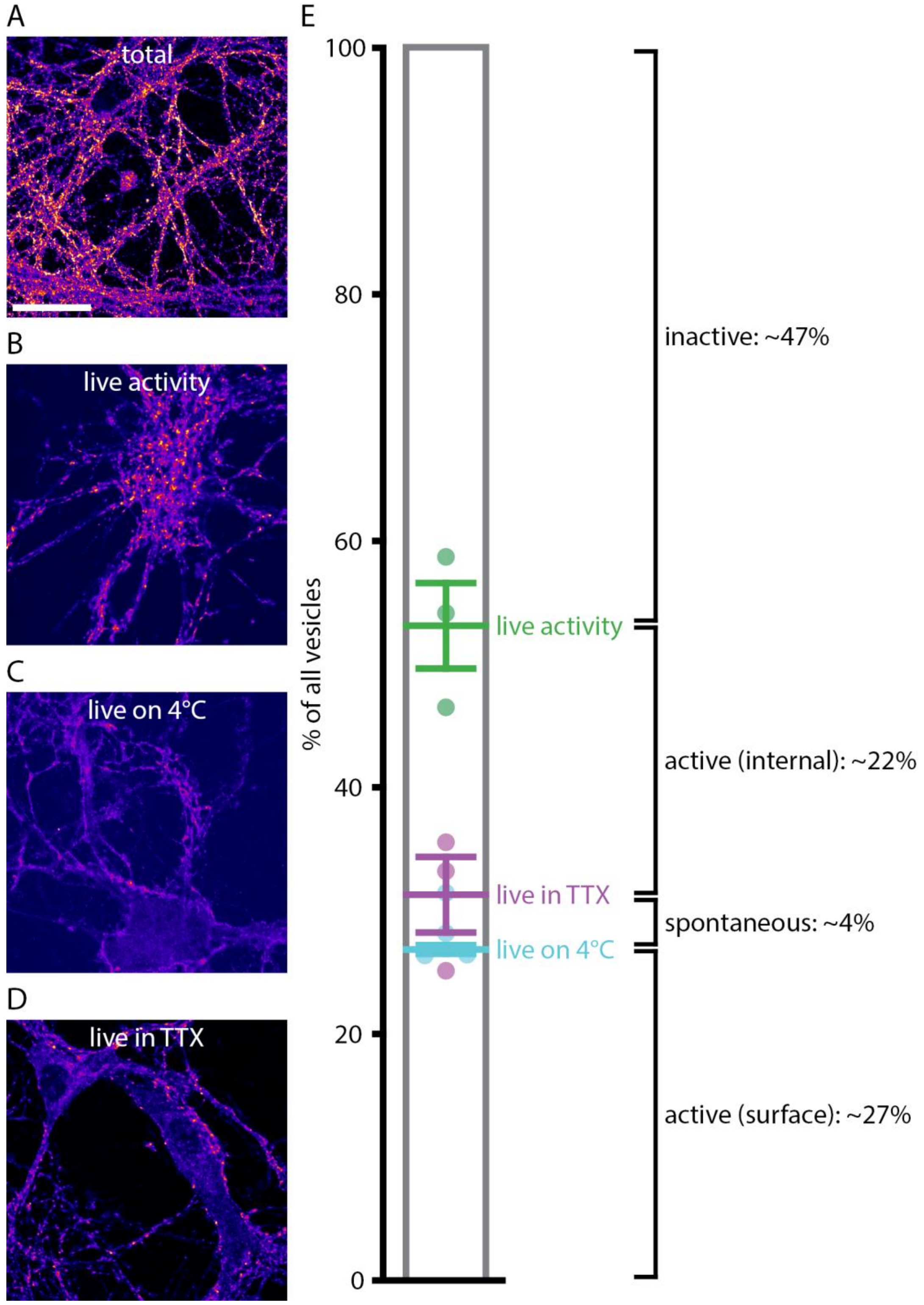

Appendix Figure S7: An analysis of the fraction of Synaptotagmin 1 molecules labeled by the antibodies.

We set out to determine the sizes of the different functional vesicle molecule pools (as known from the literature) that are labeled by the Synaptotagmin 1 antibodies.

(A) We determined the intensity of the entire vesicle pool by fixing neurons and immunostaining them with Atto647N-conjugated Synaptotagmin 1 antibodies, thus revealing all Synaptotagmin 1 epitopes. Scale bar: 20 µm.

(B) In separate coverslips from the same cultures, we determined the intensity of the actively recycling pool by incubating the living neurons with Atto647N-conjugated Synaptotagmin 1 antibodies for 1 hour at 37°C, as in Fig 1A.
(C) To split this latter pool into surface Synaptotagmin 1 epitopes and internalized Synaptotagmin 1 epitopes, we measured the former by applying the antibodies at 4°C, which reveals only the surface-exposed molecules.
(D) Finally, to reveal the vesicle pool that recycles spontaneously, in the absence of action potential stimulation, we incubated the neurons with the antibodies in presence of tetrodotoxin (TTX). Please note that this also labels the surface pool, so the sum of the two is measured in this experiment.
(E) All vesicle pools (Synaptotagmin 1 molecule fractions) are represented as percentage of all vesicles (of the entire vesicle pool intensity; n = 3 independent experiments per data point, at least 10 neurons sampled per experiment). The following definitions are used here: active = releasable vesicles that recycle in response to stimulation; these are found in vesicles from the interior of the bouton, or fused to the plasma membrane (on the surface); spontaneous = vesicles that recycle when action potentials are blocked by tetrodotoxin (TTX); inactive = non-recycling vesicles. All data represent the mean ± SEM.
Data information: All images were taken using a Leica SP5 confocal microscope. For further information, see source data.

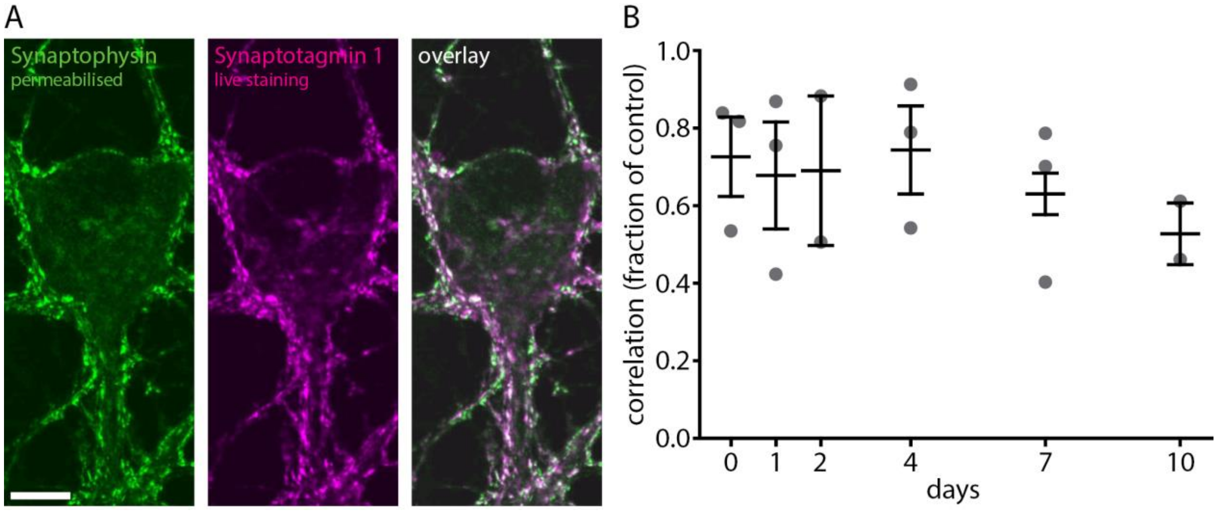

Appendix Figure S8: The Synaptotagmin 1 antibodies remain in synapses for long time periods (days).

(A) We set out to determine whether the Synaptotagmin 1 antibodies remain in synapses for long periods of time, or whether they segregate from synaptic areas. We performed live antibody stainings for Synaptotagmin 1, by incubating the neurons with Atto647N-conjugated Synaptotagmin 1 antibodies for 1 hour at 37°C, as in Fig 1A, and we then fixed and permeabilized the neurons, and immunostained them for the synaptic vesicle marker Synaptophysin. As expected from previous studies, the two stainings correlate well. Scale bar: 10 µm.

(B) We then compared the correlation between the two vesicle markers in neurons that were not immediately fixed, but were returned to the incubator, and were retrieved for fixation and Synaptophysin immunostaining at different time points. The correlation remains high throughout the entire duration of the experiment (data are from the experiments shown in Fig 1B,C). The Pearson's correlation coefficient of the two stainings was calculated, and is shown as fraction of a positive control (two-color immunostaining of Synaptophysin). No statistically significant differences were determined via one-way ANOVA, $p = 0.8582$, $F(5, 15) = 0.37$. All data represent the mean ± SEM.

Data information: All images were taken using a Leica SP5 confocal microscope. For further information, see source data.

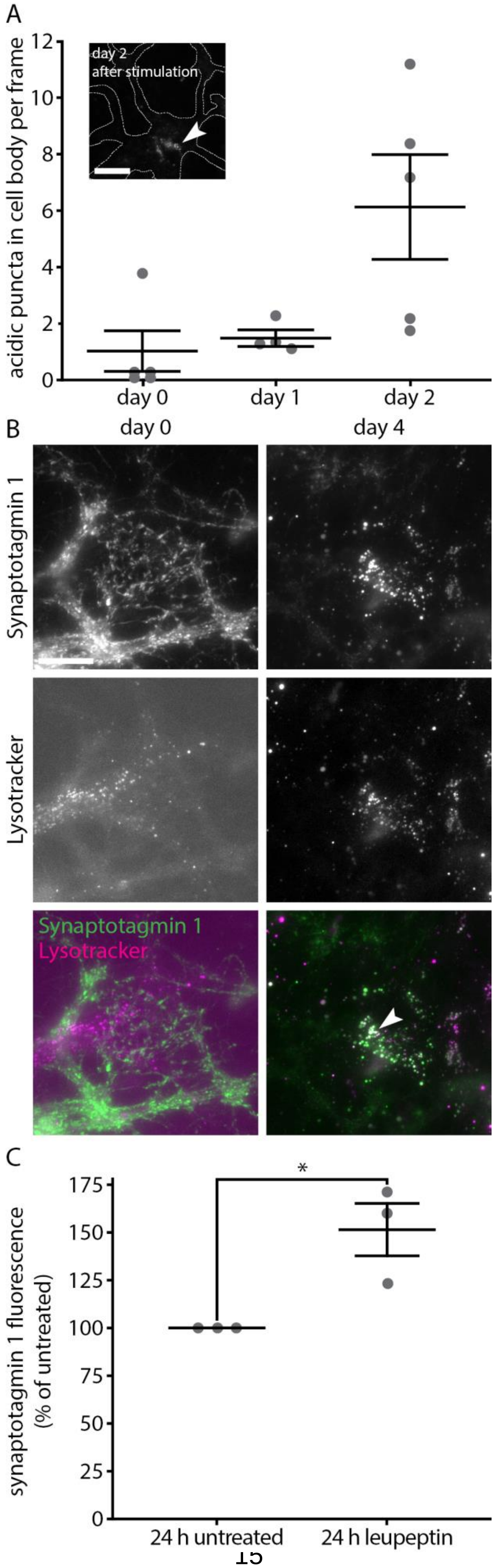
A
acidic puncta in cell body per frame
day 2
after stimulation
day 0
day 1
day 2
B
day 0
day 4
Synaptotagmin 1
Lysotracker
Synaptotagmin 1
Lysotracker
C
synaptotagmin 1 fluorescence
(% of untreated)
*
24 h untreated
24 h leupeptin

Appendix Figure S9: The Synaptotagmin 1 antibodies appear in lysosomes in the neuronal cell body after a few days of incubation.

(A) To determine whether, as expected from the literature, the Synaptotagmin 1 molecules were degraded in lysosomes, we imaged the neuronal cell bodies carefully. Large, highly fluorescent organelles became evident in the cell bodies of neurons at 2 days after live tagging of Synaptotagmin 1 molecules with fluorophore-conjugated Synaptotagmin 1 antibodies (n = 5, 4, and 5 independent experiments per time point). We used the pH-sensitive CypHer5E fluorophore in this experiment, as this enabled us to test whether the respective organelles engaged in exocytosis upon stimulation. They did not (see inset image). Their acidity, size, localization and the time of occurrence of antibody fluorescence are consistent with lysosomes. Scale bar: 10 µm.

(B) We corroborated the findings from (A) by performing the same Synaptotagmin 1 antibody uptake experiment, but with Atto647N-conjugated antibodies, and then applied Lysotracker immediately after the labeling with the antibodies, or four days later. We observed no co-localization of Synaptotagmin 1 (syt1) antibody with Lysotracker (lyso) in the cell body directly after antibody uptake. After four days of incubation, the Synaptotagmin 1 antibody was present in the lysosomes of the cell body, indicating that it was targeted, presumably along with Synaptotagmin 1, to these organelles, thereby recapitulating the life cycle of synaptic vesicle predicted by the literature. Scale bar: 20 µm.

(C) To further test whether the Synaptotagmin 1 molecules were degraded in lysosomes, we incubated neurons with Atto647N-conjugated Synaptotagmin 1 antibodies, as in Fig 1A, for 1 hour at 37°C, and then placed them in the incubator for 24 hours. During this period one set of cells was treated with the lysosomal degradation inhibitor leupeptin. We then determined the loss of Synaptotagmin 1 antibody fluorescence over the 24 h period, by comparing coverslips treated for 24 hours with coverslips imaged immediately after the initial labeling. The results suggest that the vesicles are indeed degraded by lysosomal activity, since inhibiting lysosomal degradation maintains the levels of Synaptotagmin 1 labeling at higher levels in the synapses than in the control case (n = 3 independent experiments per data point, at least 10 neurons sampled per experiment; $^*p = 0.0237$, $t(4) = 3.5561$).

Data information: All data represent the mean ± SEM. Statistical significance was evaluated using an unpaired t-test. All experiments in panels (A) and (C) were imaged using a Leica SP5 confocal microscope. The experiments in (B) were imaged using a Nikon Ti-E epifluorescence microscope. For further information, see source data.

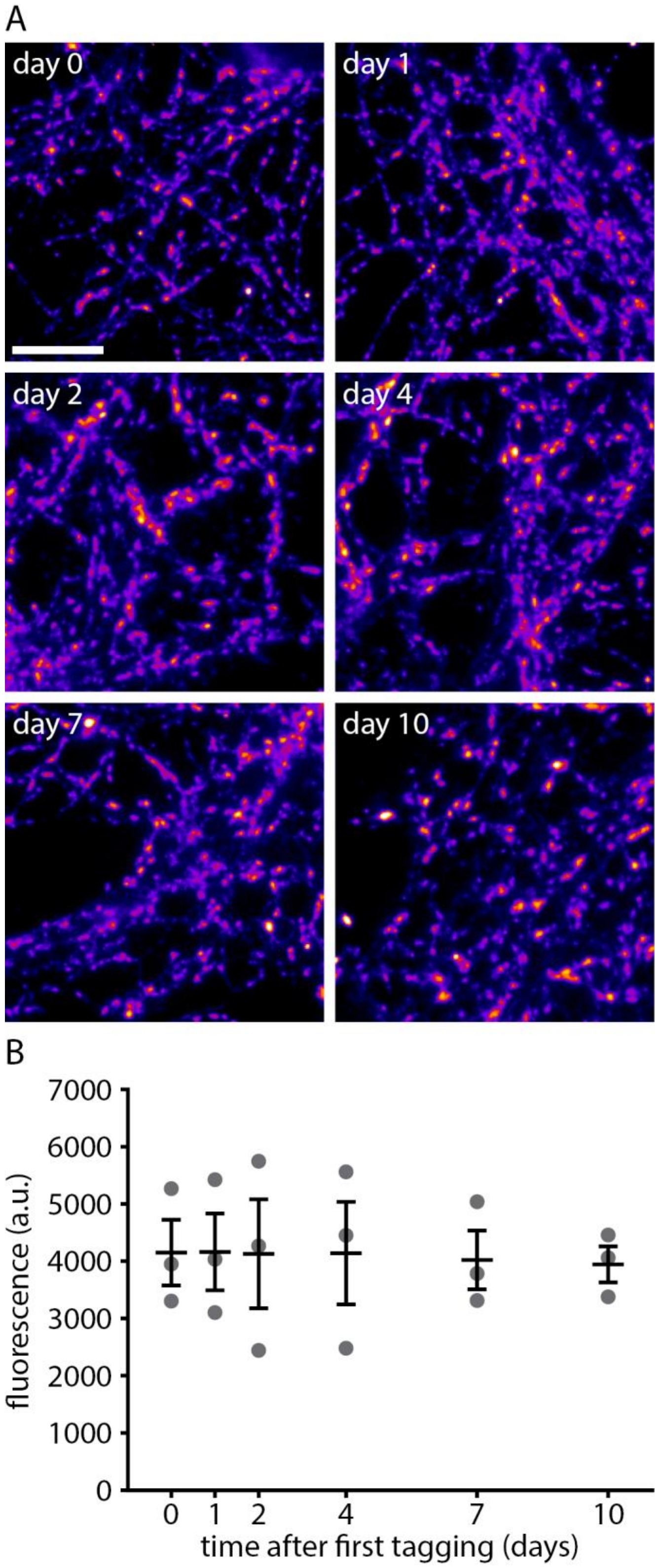

Appendix Figure S10: Neurons of different ages show similar levels of synaptic activity. (A) To exclude the possibility that synaptic vesicle release activity of our cultures changed during our 10-day time course experiments, which might be an alternative explanation for the loss of tagged synaptic vesicle releasability we observed in Fig 1, we performed synaptic vesicle tagging with the Synaptotagmin 1 antibody on each of the indicated days, up to 10 days after the first tagging experiment. We then immediately fixed the cultures and measured the

Synaptotagmin 1 uptake intensity by imaging with an epifluorescence Nikon Ti-E microscope. Scale bar: 10 µm.

(B) An analysis of the intensities at different time points indicated that the release activity of the cultures remained constant (n = 3 independent experiments per time point). No statistically significant differences were determined via one-way ANOVA, $p = 0.3046$, $F(5, 17) = 1.36$. All data represent the mean ± SEM.

Data information: All imaging was performed on a Nikon Ti-E epifluorescence microscope. For further information, see source data.

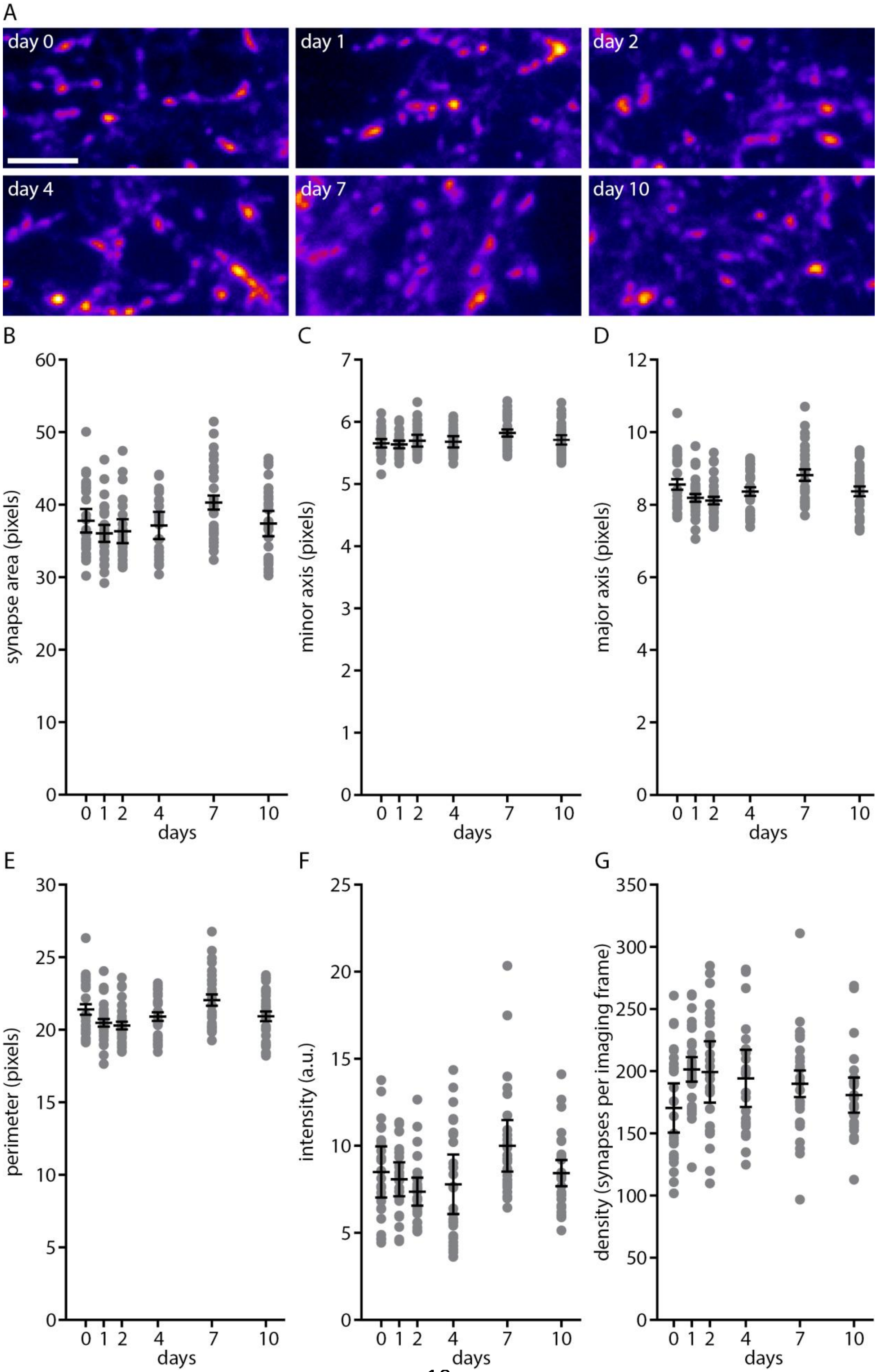
A
day 0
day 1
day 2
day 4
day 7
day 10
B
synapse area (pixels)
days
C
minor axis (pixels)
days
D
major axis (pixels)
days
E
perimeter (pixels)
days
F
intensity (a.u.)
days
G
density (synapses per imaging frame)
days

Appendix Figure S11: Neurons of different ages have similar densities of synaptic boutons, as well as similar bouton sizes and vesicle amounts.

(A) We immunostained cultures of different ages, over a 10-day time course, starting at 14 days *in vitro* (d.i.v.) of the cultures, for Synaptophysin, as a synaptic vesicle marker, and thereby also synaptic bouton marker. Representative images, taken with a Nikon Ti-E epifluorescence microscope, are shown. Day 0 represents the first day of the experiment (14 d.i.v.). Scale bar: 5 µm.

(B-G) We analyzed the synaptic boutons, determining their area (B), minor axis (C), major axis (D), perimeter (E), as well as their intensities (F; as a measure of the vesicle amounts) and densities (G). All of these parameters change little during the 10-day course ($n = 3$ independent experiments per time point, with 9-10 individual neurons imaged in each experiment). All data represent the mean ± SEM. No statistically significant changes occur over time in any of the measured parameters, as tested in one-way ANOVA: (B) $p = 0.4743$, $F(5, 17) = 0.97$; (C) $p = 0.3046$, $F(5, 17) = 1.36$; (D) $p = 0.6040$, $F(5, 17) = 0.75$; (E) $p = 0.6899$, $F(5, 17) = 0.62$; (F) $p = 0.3462$, $F(5, 17) = 1.25$; (G) $p = 0.7542$, $F(5, 17) = 0.52$.

Data information: All imaging was performed on a Nikon Ti-E epifluorescence microscope. For further information, see source data.

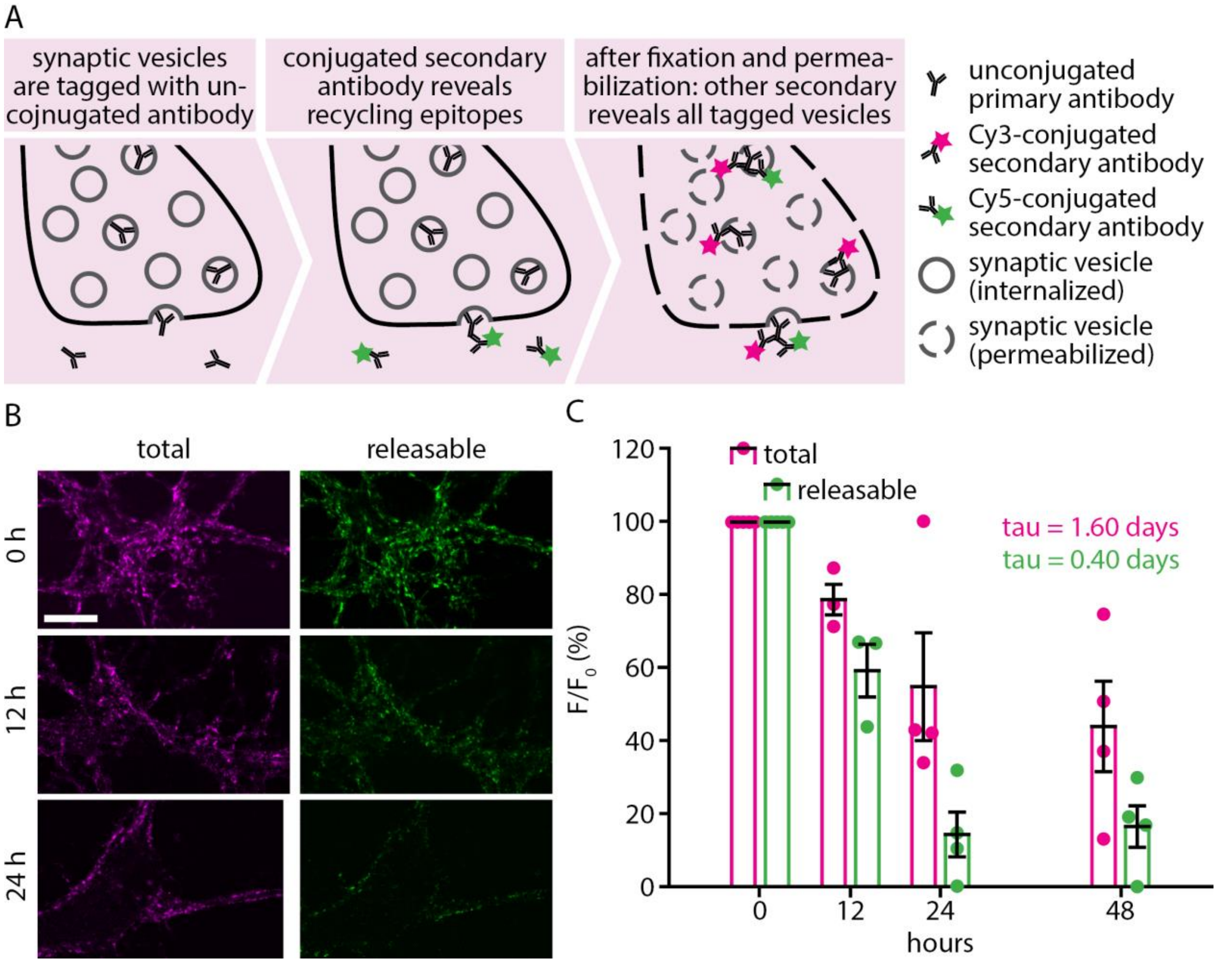

Appendix Figure S12: Recycling Synaptotagmin 1 proteins become inactive within less than a day during spontaneous network activity.

(A) To investigate the behavior of the actively recycling Synaptotagmin 1 molecules under normal (unstimulated) network activity, we tagged them with non-fluorescently conjugated antibodies at one time point, by incubation for 1 hour at 37°C, as in Fig 1A. We then, after different time intervals, from 12 hours to 48 hours, applied Cy5-conjugated secondary antibodies (green) onto the living cultures. These antibodies reveal all of the Synaptotagmin 1 antibodies that are exposed during vesicle recycling. After incubating the living cultures with the secondary antibodies for one hour, we fixed and permeabilized them, and applied Cy3-conjugated secondary antibodies (magenta), to reveal all of the remaining Synaptotagmin 1 antibodies. This procedure thus indicates the proportion of the tagged Synaptotagmin 1 molecules that are still recycling in the synapses under normal activity (shown in green), as well as the proportion that are present in the synapses (shown in magenta).

(B) Exemplary images of dually labeled synapses, as revealed at different time intervals after the initial incubation with non-fluorescently conjugated Synaptotagmin 1 antibodies. Scale bar: 10 µm.

(C) We quantified the green and red intensity at different time points, and expressed them as percentages of their respective values at the 0 hour time point. The fraction of the vesicles that are still actively recycling (green) decreases more rapidly than the fraction that are present in the synapse in an inactive state (magenta). One could thus conclude that synaptic vesicle proteins lose the ability to release during spontaneous network activity within less than a day, but they persist within the synapse in an inactive state for much longer (n = 5, 3, 4, 4

independent experiments per respective time point, at least 10 neurons sampled per experiment). All data represent the mean ± SEM.

Data information: All experiments were imaged using a Leica SP5 confocal microscope. For further information, see source data.

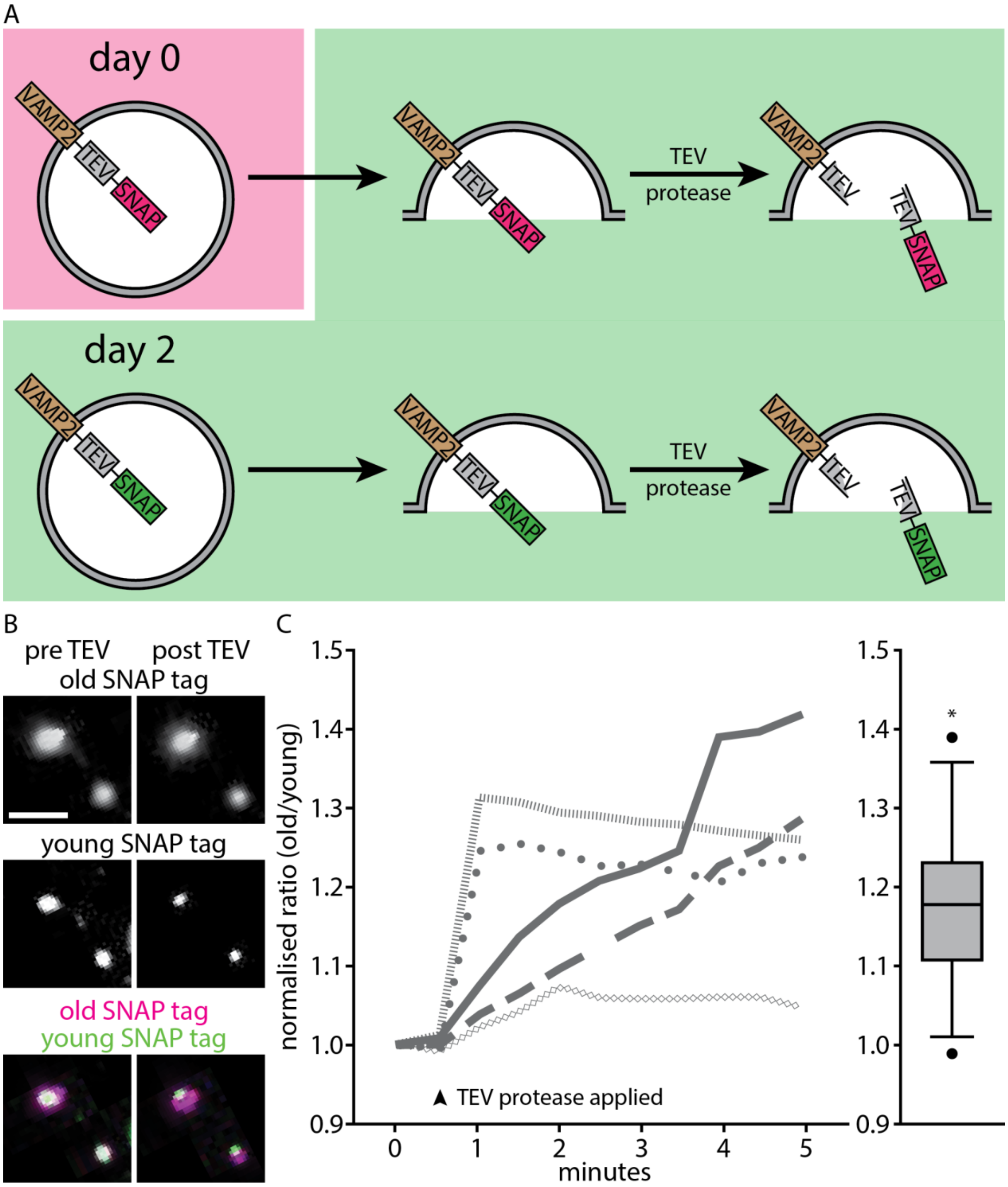

Appendix Figure S13: Young overexpressed VAMP2 molecules are released preferentially.

(A) We expressed in the neurons VAMP2 coupled to a SNAP-tag, and separated from the original sequence by a TEV protease cleavage site: VAMP2-TEV-SNAPtag. The neurons were allowed to express this construct for 3-4 days, and then the VAMP2 moieties were labeled by applying a membrane-permeable fluorophore, tetramethyl-rhodamine-Star (TMR-Star; magenta). Then the fluorophore was washed off, and the neurons were returned to their incubator for an additional day, when a second fluorophore was applied, 647-SiR (green). This fluorophore thus labels all of the VAMP2 moieties that had been synthesized since the last labeling procedure. The cells were then removed from the incubator, were placed in a live

imaging chamber at 37°C, and were allowed to recycle vesicles normally, under the physiological rate of network activity. After collecting the initial imaging frames, the TEV protease was applied to the medium to cleave the labelled SNAP tags from the recycling vesicles. The cleavage of green-coupled tags implies that young vesicles are used in recycling. The cleavage of red-coupled tags indicates the opposite – the older red-coupled VAMP2 molecules are used in recycling.
(B) Exemplary images for labelled young and old vesicles in the same synapses, before or after TEV protease cleavage. Scale bar: 2 µm.
(C) Quantification of the normalized intensity ratio between the old (magenta) and young (green) VAMP2 molecules during the experiment. The TEV protease application is indicated by the arrow. For clarity, 5 representative traces are shown out of a total of 11 independent experiments. Despite the differences in kinetics, which reflect the differences in network activity between the cultures, it is apparent that the ratio increases in all experiments. This implies that the levels of old-tagged molecules (red) increase over the young-tagged ones (green), meaning that the latter are preferentially removed by the TEV protease. The inset shows the difference in ratio after five minutes of TEV protease application (n = 11 independent experiments), which was significant compared to the last data point before application of the TEV protease (*p = 0.0001, t(20) = 4.9700). Average data are represented as box plots with median and upper and lower quartile boundaries, plus 1.5-times inter-quartile range (whiskers) and outliers (dots). Statistical significance was evaluated using an unpaired t-test.
Data information: All experiments were imaged using a Nikon Ti-E epifluorescence microscope, at 37°C. For further information, see source data.

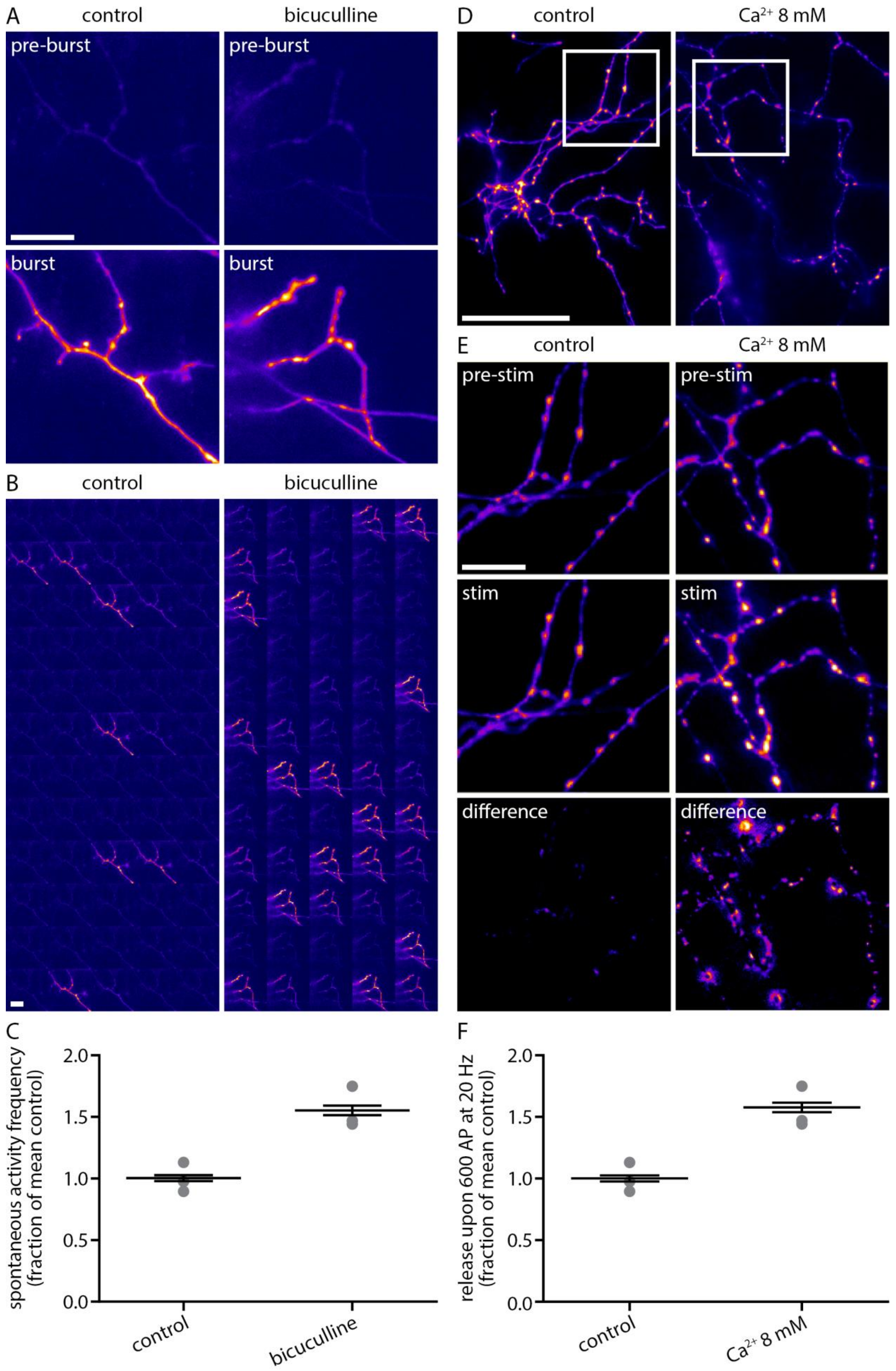
A
control
bicuculline
pre-burst
pre-burst
burst
burst
B
control
bicuculline
C
spontaneous activity frequency (fraction of mean control)
2.0
1.5
1.0
0.5
0.0
control
bicuculline
D
control
Ca2+ 8 mM
E
control
Ca2+ 8 mM
pre-stim
pre-stim
stim
stim
difference
difference
F
release upon 600 AP at 20 Hz (fraction of mean control)
2.0
1.5
1.0
0.5
0.0
control
Ca2+ 8 mM

Appendix Figure S14: Quantification of the effects of bicuculline or 8 mM Ca^{2+} on neuronal activity.

Neurons were incubated for 12 hours with 20 µM of the $GABA_A$ receptor antagonist bicuculline, or with a Ca^{2+} concentration raised to 8 mM. The neuronal activity was then analyzed as follows.

(A) The neurons were transfected with the calcium sensor protein GCaMP6, and the spontaneous activity of the cultures was determining by measuring the changes in the GCaMP6 fluorescence. Typical images are shown, scale bar: 15 µm.

(B) Typical intensity traces for a control neuron (left) and a bicuculline-treated neuron (right). The image sequence is from left to right and top to bottom, and the images in the time course were taken 2 s apart. Scale bar: 15 µm.

(C) An analysis of the activity (burst) frequency indicates that bicuculline increases the spontaneous culture activity by ~1.5-fold (n = 3). This difference is statistically significant: p = 0.0100, t(4) = 4.6005.

(D) The same experiment could not be performed in cultures treated with 8 mM Ca^{2+}, as this seemed to interfere with the GCaMP6 measurements. To determine whether the cultures were still more activity-prone after this incubation, we resorted to pHluorin imaging, using a Synaptophysin-based pHluorin construct (sypHy). The images show typical sypHy images before and during stimulation at 20 Hz (600 APs). The pHluorin moiety is quenched in the acidic pH of the vesicle lumen, and is more fluorescent upon exocytosis. Typical images are shown, scale bar: 50 µm.

(E) Typical pHluorin signals before and after stimulation, for a control neuron (left) and a Ca^{2+} 8 mM-treated neuron (right). Scale bar: 15 µm.

(F) An analysis of the fraction of all vesicles that were exocytosed, determined after normalizing the traces to the maximal fluorescence obtained after incubation with 100 mM NH_4^+, which neutralizes the vesicular activity. This implies that the treated cultures are more release-prone upon activation (~1.5-fold; n = 3). This difference is statistically significant: p = 0.0175, t(4) = 3.9012.

Data information: All data represent the mean ± SEM. Statistical significance was evaluated using the unpaired t-test. All images were taken using a Nikon Ti-E epifluorescence microscope, at 37°C. For further information, see source data.

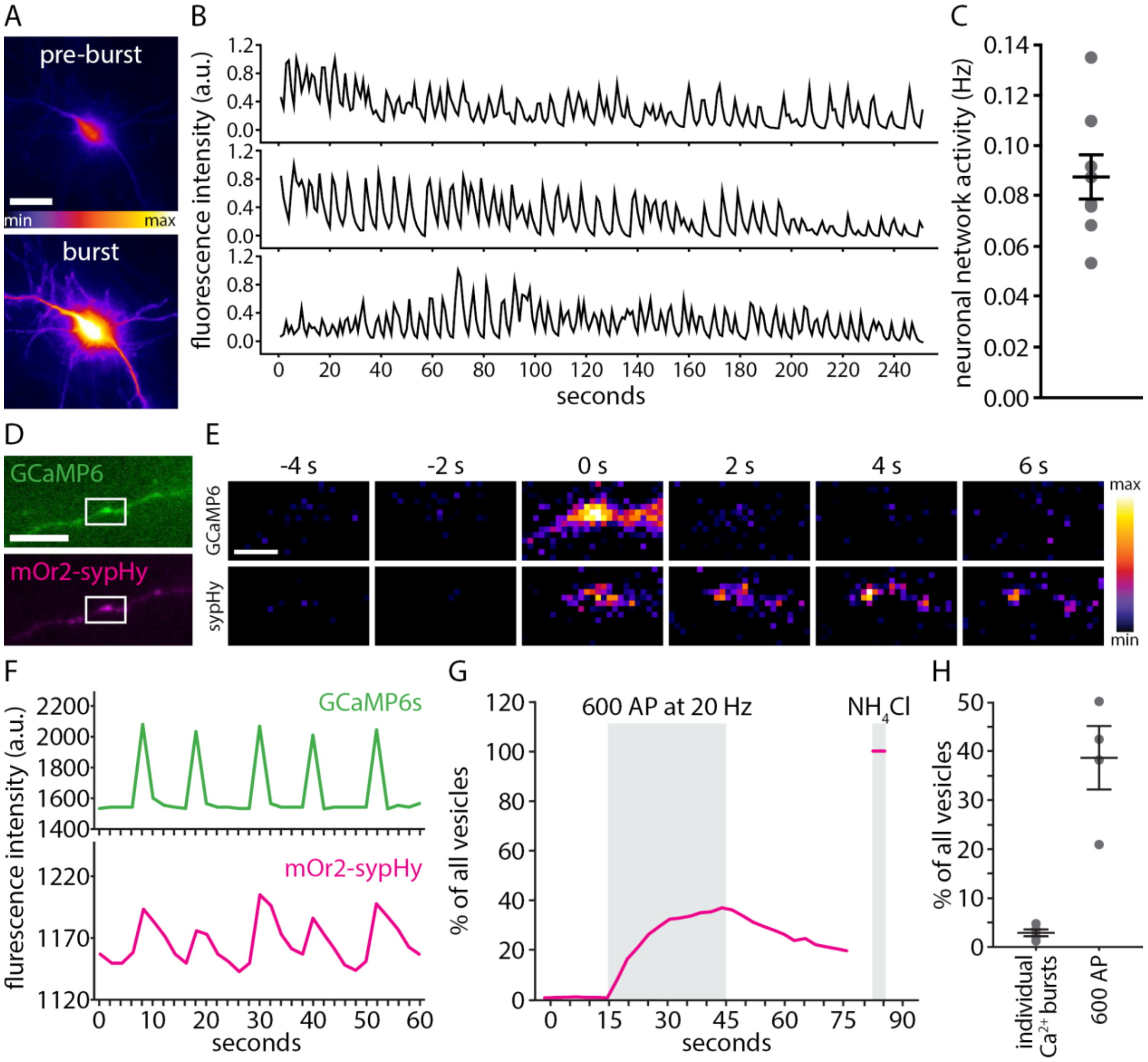

Appendix Figure S15: An analysis of the synaptic vesicle recycling under spontaneous network activity.

(A) To determine the frequency of neuronal activity under spontaneous (non-stimulated) conditions, neurons were transfected with the calcium sensor GCaMP6 for 3-4 days, and then imaged using a Nikon Ti-E microscope (epifluorescence). The panels show typical images of a neuron during a high activity phase (burst; high Ca^{2+} concentration) or during a low activity phase (one second before the burst, written as burst -1 s; low Ca2+ concentration). Scale bar 20 µm.

(B) Exemplary traces of spontaneous network activity in hippocampal cultures. The fluorescence signal from cells such as those in panel (A) was measured for several minutes, and is shown here as arbitrary fluorescence units.

(C) We analyzed the average frequency of the activity bursts in hippocampal cultures (n = 7 experiments), from traces as those shown in panel (B).

(D) To determine the fraction of the vesicles that recycle during one burst of activity, we co-transfected neurons with GCaMP6 and with the vesicle recycling sensor sypHy, and we then assessed the increases in sypHy fluorescence coinciding with activity bursts. Scale bar 2 µm.

(E) Exemplary images of changes in GCaMP6 and sypHy fluorescence during a pulse of spontaneous network activity (white box magnified from (E)).

(F) Exemplary traces of activity (GCaMP6 signal) and exocytosis levels (sypHy signal) in hippocampal cultures.
(G) As a comparison to the spontaneous exocytosis measurements from (D-F), we show here an exemplary trace indicating exocytosis (sypHy signal) during a train of electrical stimulation with 600 action potentials (AP) at 20 Hz. This stimulus releases the entire recycling pool (Wilhelm *et al*, 2010). To assess the fraction of the vesicles that were released (the fraction of the sypHy molecules that are located in the recycling pool), a pulse of NH_4Cl was applied at the end of the experiment, which reveals all sypHy molecules, both in the recycling and in the inactive pool.
(H) We quantified the amount of sypHy exocytosed during one activity burst, from traces as shown in panel (G), as percentage of the total amount of sypHy, revealed by a pulse of NH_4Cl, as in panel (H). As a comparison, the recycling pool, revealed by stimulation with 600 action potentials (AP) at 20 Hz (as in panel h) is shown. (n = 4 experiments for both conditions).

The parameters determined here can be then used to estimate the average number of recycling rounds undergone by a vesicle before inactivation, using the following formula:

$$N = 2 * \tau * f * \frac{F_{burst}}{F_{releasable}} * \frac{F_{internalized}}{F_{releasable}}$$

where

- N is the number of recycling events per synaptic vesicle, per lifetime
- 2 is a correction factor for the total time the vesicle spends in the recycling pool. This correction factor is introduced to take into account the fact that synaptic vesicles are only observed to be in the recycling pool after they are tagged with the Synaptotagmin 1 antibody. Since this parameter probably follows a Gaussian distribution, it has to be assumed that the vesicles are tagged, on average, for only half of the total time they spend in the releasable population.
- τ is the time constant of synaptic vesicles staying in the recycling pool (obtained from Appendix Fig S12C), which is equivalent to the total amount of time a single vesicle spends in the recycling pool. As indicated for the previous parameter, this factor underestimates the real time period by 2-fold, and therefore requires “2” as a correction factor. As indicated in Appendix Fig S12C, τ = 0.4 days.
- f is the frequency of activity bursts in the cultures, as measured by Ca^{2+} imaging. As indicated in Appendix Fig S15C, f = 0.09 Hz.
- $\frac{F_{burst}}{F_{releaseable}}$ is the fraction of the releasable population that is released during each neuronal activity burst, as measured by pHluorin imaging. As indicated in Appendix Fig S15H, $\frac{F_{burst}}{F_{releaseable}}$ = 0.075 (single Ca^{2+} burst divided by 600 AP).
- $\frac{F_{internalized}}{F_{releaseable}}$ is the fraction of the releasable population that is internalized, and is therefore available for release during activity bursts. As indicated in Appendix Fig S7E, $\frac{F_{internalized}}{F_{releaseable}}$ = 0.45 (internalized fraction of the releasable population divided by total releasable population).

Data information: The data in (C) and (H) represent the mean ± SEM. All images were taken using a Nikon Ti-E epifluorescence microscope, at 37°C. For further information, see source data.

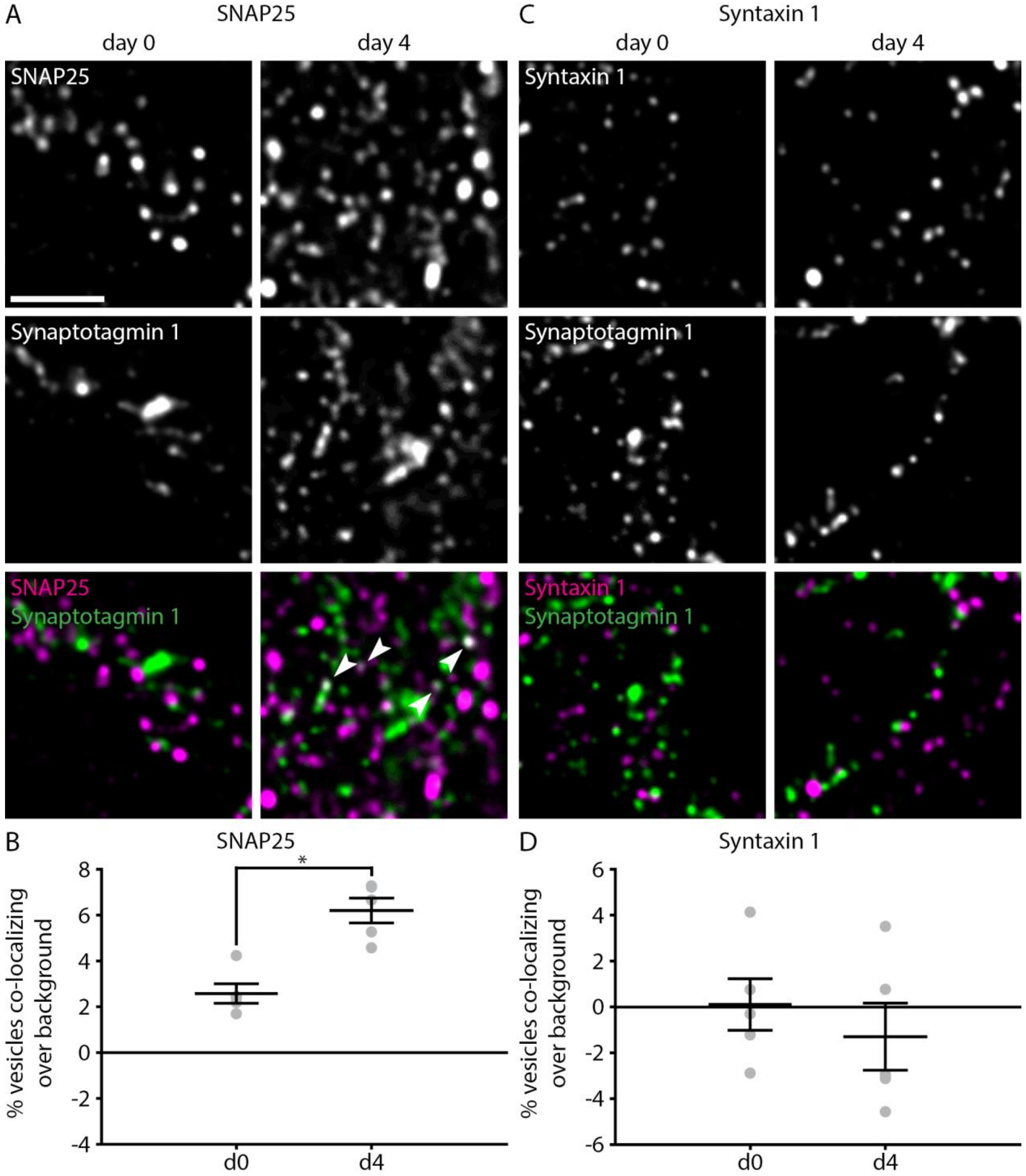

Appendix Figure S16: Additional experiments confirm that ageing Synaptotagmin 1 molecules co-localize with SNAP25, but not with Syntaxin 1.

(A,C) Two-color STED analysis of changes in synaptic vesicle protein levels during the transition from the releasable state to the inactive state, as in Fig 8. Living neurons were incubated with Atto647N-conjugated Synaptotagmin 1 lumenal domain antibodies for 1 hour at 37°C, to label the actively recycling vesicles, as in Fig 1, and were then fixed and co-immunostained for different proteins of interest directly, or were placed in a cell culture incubator for 3-4 days, to enable the antibody-labeled molecules to enter the inactive pool, before fixation and co-immunostaining. The samples were embedded in Mowiol, before 2-color 3D STED imaging, using an Abberior STED setup.

Exemplary images are shown in for Syntaxin 1 and SNAP25. Scale bar: 1 µm.

(B,D) We then analyzed the co-localization of the Synaptotagmin 1 antibody with the proteins of interest immunostaining, by drawing line-scans through each spot of the Synaptotagmin 1 staining, and correlating the respective line scan to an identical line scan drawn in the imaging channel of the protein of interest (using Pearson's correlation coefficient). We typically analyzed 1000-1300 spots in each independent experiment. The bar graphs show the percentage of Synaptotagmin 1 spots that correlated well with the protein of interest signal (correlation coefficients equal to, or larger than 0.7), corrected for the values obtained in a negative control performed after rotating horizontally (mirroring) the green images. For both SNAP25 and Syntaxin 1, we analyzed 5 datasets from 3 independent neuronal cultures. The statistical analysis shows a significant increase in SNAP25 from day 0 to day 4 (*$p = 0.0008$, $t(8) = 5.1912$), but not for Syntaxin 1 ($p = 0.4903$, $t(8) = 0.7229$).
Data information: All data represent the mean ± SEM. Statistical significance was evaluated using unpaired t-tests. All imaging was performed using two-color STED microscopy on an Abberior 3D STED setup. For further information, see source data.

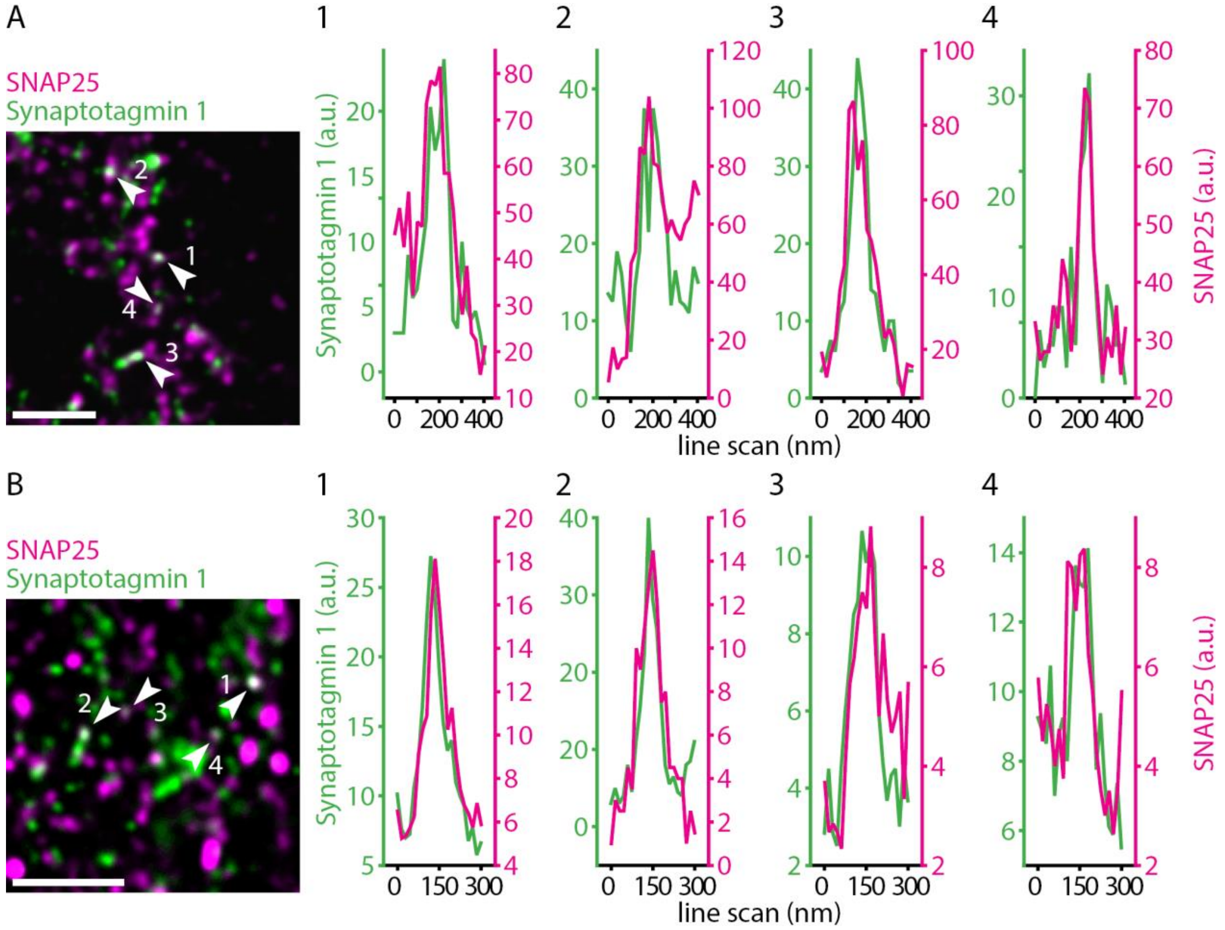

Appendix Figure S17: The co-localization of SNAP25 with Synaptotagmin 1 is high for aged Synaptotagmin 1 molecules.

(A) Four exemplary line scans are shown for the exemplary images for the “day 4” condition presented in Fig 8. Scale bar: 1 µm. The line scans are performed over the spots indicated by arrowheads in the respective figure.

(B) Four exemplary line scans are shown for the exemplary images for the “day 4” condition presented in Appendix Fig S16. Scale bar: 1 µm. The line scans are performed over the spots indicated by arrowheads in the respective figure.

Data information: The experiments were performed as described in the legends of Fig 8 and Appendix Fig S16. See the respective figures for more information.

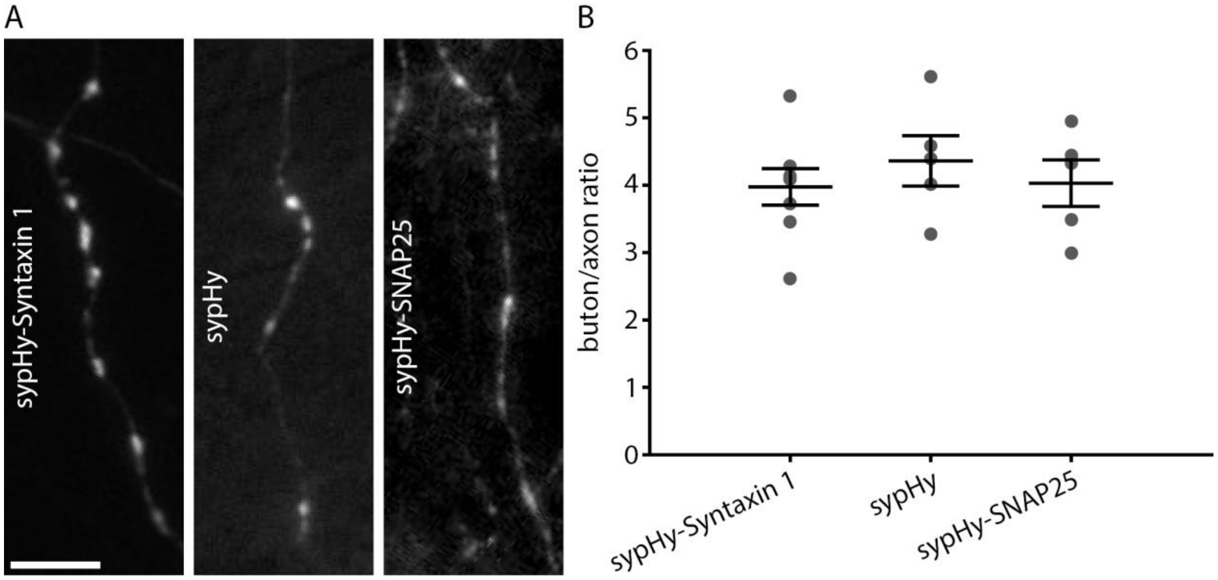

Appendix Figure S18: SypHy, sypHy-SNAP25, and sypHy-Syntaxin 1 show similar patterns of expression in hippocampal neurons.

(A) Neurons were transfected with the indicated constructs, and were imaged after the addition of NH_4^+, which neutralizes the vesicular pH, and thereby reveals all of the sypHy moieties. Scale bar: 10 µm.

(B) We analyzed the intensity of the inter-bouton areas, and compared it to the intensity of the boutons. Differences in this parameter would indicate different levels of sypHy expression in the non-vesicular, axonal compartment. No such differences could be observed in an ANOVA test (n = 7 for sypHy-Syntaxin 1, n = 5 for sypHy, n = 5 for sypHy-SNAP25; no statistically significant differences as determined via one-way ANOVA, $p = 0.6816$, $F(2, 17) = 0.39$). All data represent the mean ± SEM.

Data information: All imaging was performed with a Nikon Ti-E epifluorescence microscope, at 37°C. For further information, see source data.

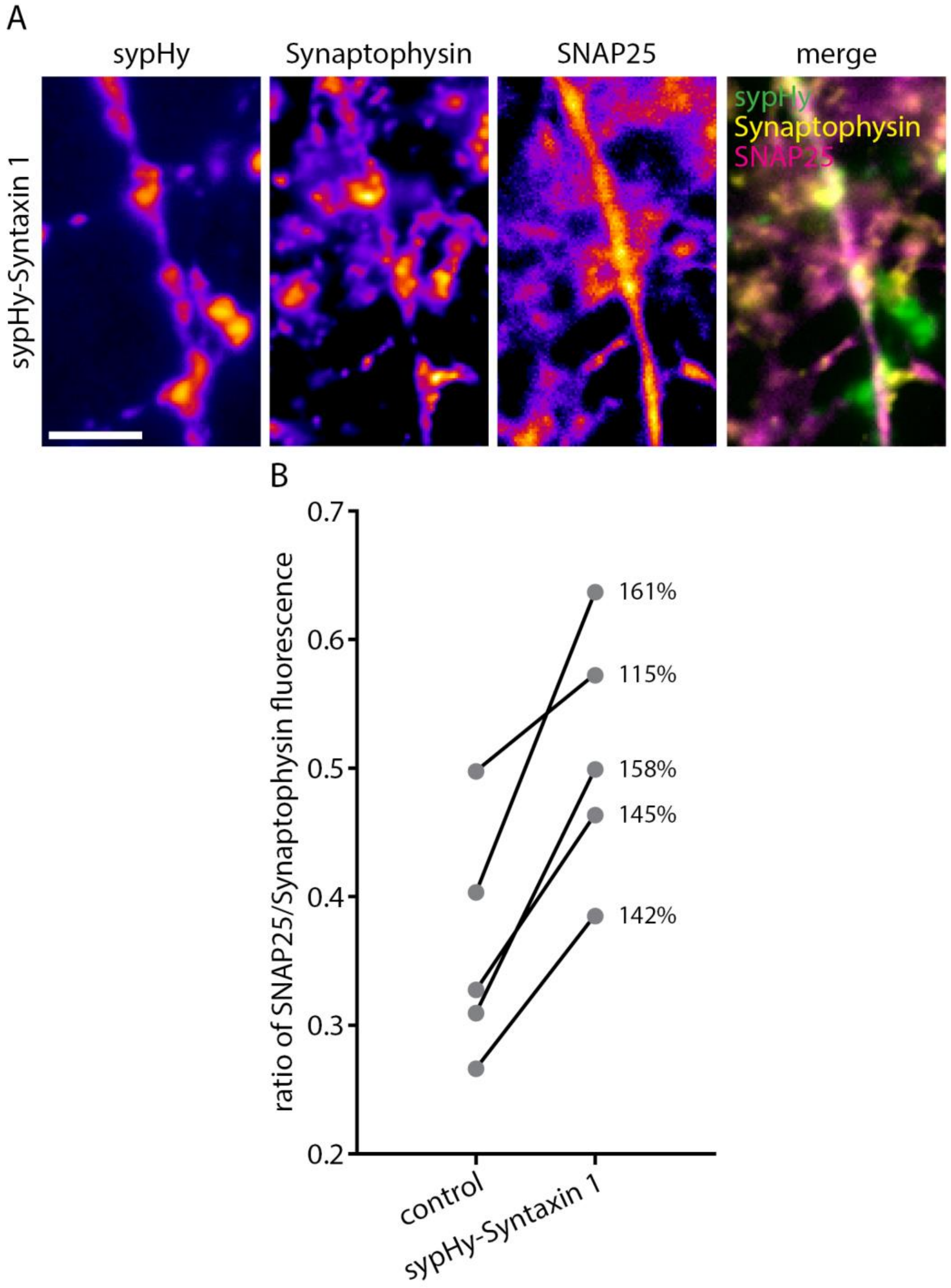

Appendix Figure S19: Targeting Syntaxin 1 to synaptic vesicles increases the amount of SNAP25 in synaptic boutons.

(A) Exemplary images show cells overexpressing sypHy-Syntaxin 1. A co-immunostaining for Synaptophysin indicates synapses, and a co-immunostaining for SNAP25 reveals the amount of that protein inside synapses overexpressing sypHy-Syntaxin 1 or without overexpression (the boutons containing no sypHy-Syntaxin 1 in the same image). The images and analysis shown here are from the same experiments as presented in Fig 9 and Appendix Fig S26. Scale bar: 15 µm.

(B) We determined the amount of SNAP25 in the synapses relative to the amount of Synaptophysin, to account for the number of synaptic vesicles in the analyzed synapses. The increase of SNAP25 is also presented as percentage increase in the graph, on the right ($n = 5$ independent experiments, $p = 0.0387$, $t(8) = 2.4707$). The control condition is represented by

non-transfected synapses from the same imaging frames as the sypHy-Syntaxin 1 transfected synapses. Statistical significance was evaluated using a paired t-test.
Data information: All imaging was performed on a Nikon Ti-E epifluorescence microscope. For further information, see source data.

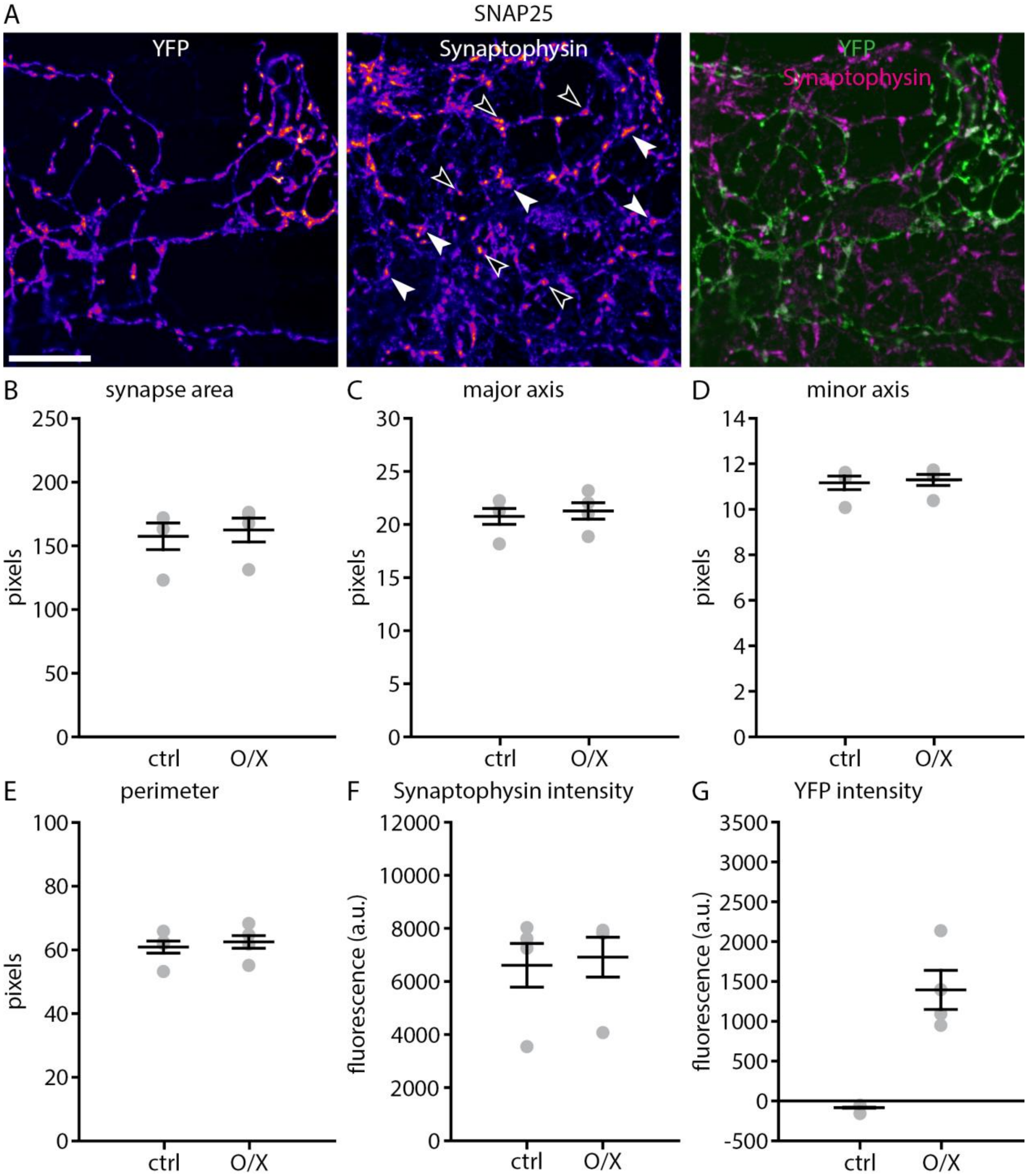

Appendix Figure S20: The expression of SNAP25 in neurons does not affect synaptic bouton morphology or synaptic vesicle amounts.

(A) Neurons were transfected with a YFP-SNAP25 construct, and were fixed and immunostained for Synaptophysin, as a synaptic vesicle marker (same data as in Fig 10). Black arrowheads indicate exemplary synapses not expressing the construct, white arrowheads indicate exemplary synapses expressing the construct. Scale bar: 10 µm.

(B-G) We analyzed the synaptic boutons, determining their area, perimeter, major and minor axes, as well as their intensities in the Synaptophysin channel (as a measure of the vesicle amounts) and in the YFP channel (as a measure of the SNAP25 expression). The measurements were split into “YFP-negative” and “YFP-positive” sets, which were averaged for each experiment. The graphs shows the mean of n = 4 independent experiments.

Data information: All data represent the mean ± SEM. All imaging was performed with a Leica SP5 confocal microscope. For further information, see source data.

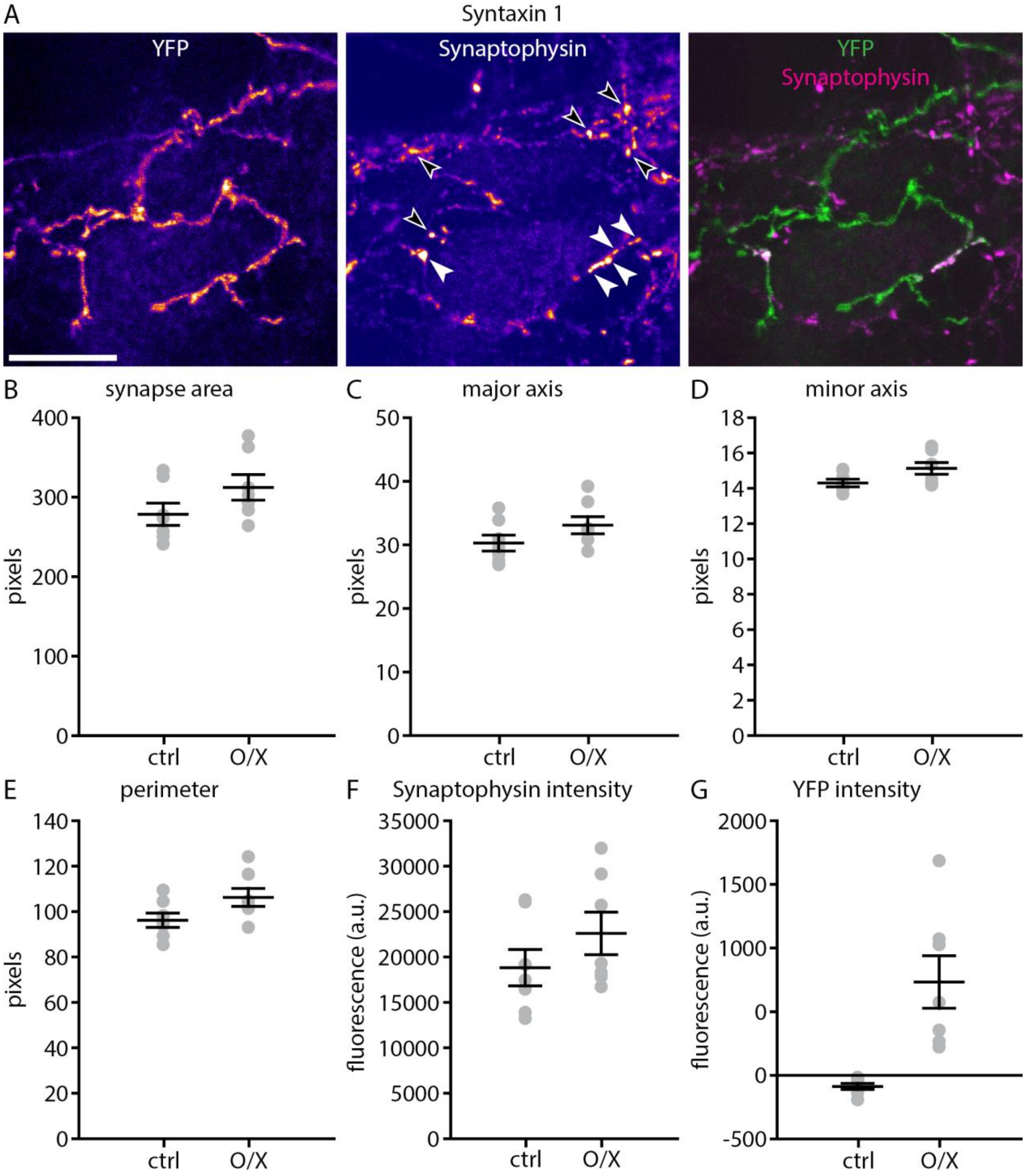

Appendix Figure S21: The expression of Syntaxin 1 in neurons does not affect synaptic bouton morphology or synaptic vesicle amounts.

(A) Neurons were transfected with a Syntaxin 1-YFP construct, and were fixed and immunostained for Synaptophysin, as a synaptic vesicle marker (same data as in Fig 10). Black arrowheads indicate exemplary synapses not expressing the construct, white arrowheads indicate exemplary synapses expressing the construct. Scale bar: 10 µm.

(B-G) We analyzed the synaptic boutons, determining their area, perimeter, major and minor axes, as well as their intensities in the Synaptophysin channel (as a measure of the vesicle amounts) and in the YFP channel (as a measure of the Syntaxin 1 expression). The measurements were split into “YFP-negative” and “YFP-positive” sets, which were averaged for each experiment. The graph shows the mean of n = 7 independent experiments.

Data information: All data represent the mean ± SEM. All imaging was performed with a Leica SP5 confocal microscope. For further information, see source data.

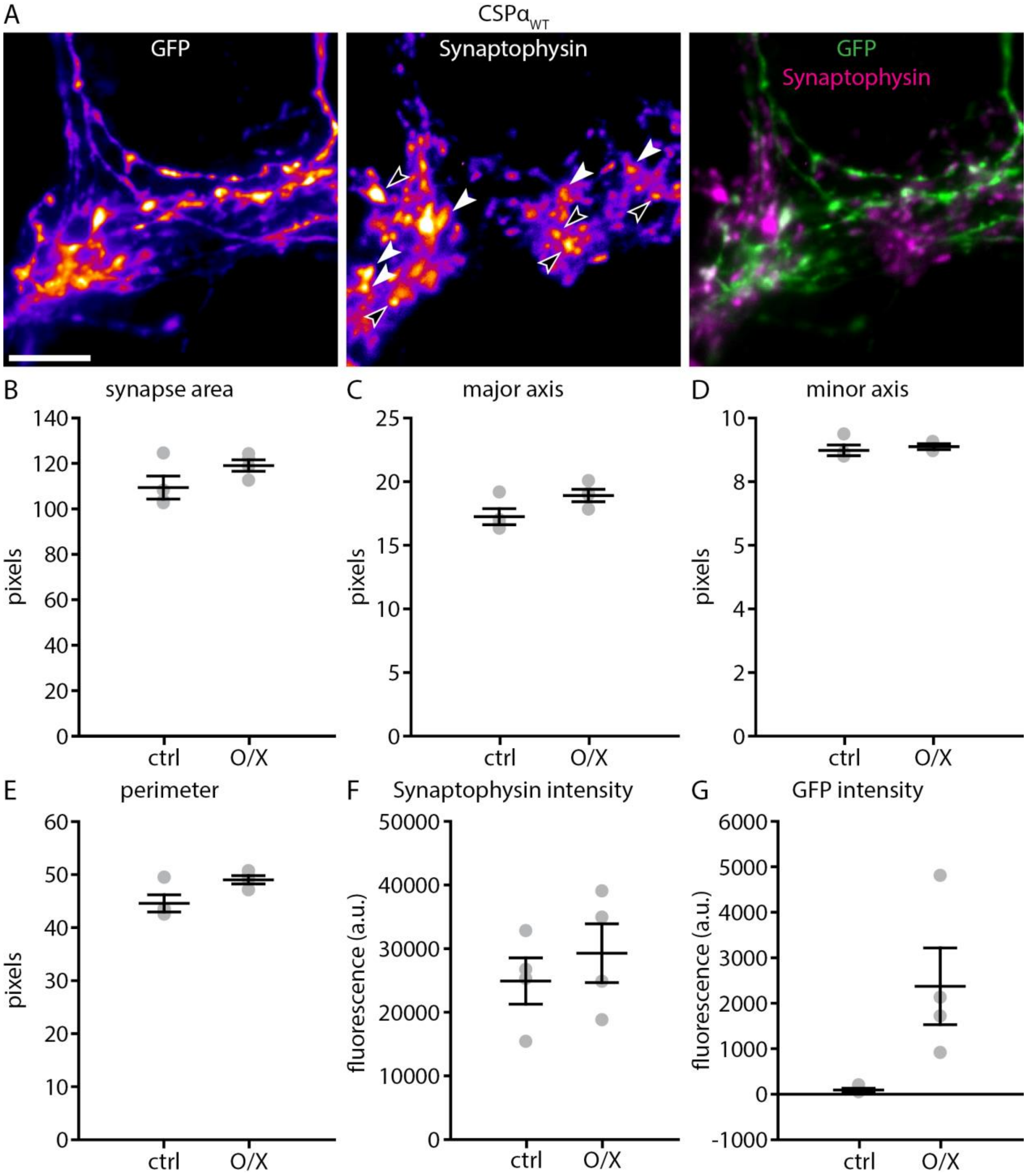

Appendix Figure S22: The expression of $CSP\alpha_{WT}$ in neurons does not affect synaptic bouton morphology or synaptic vesicle amounts.

(A) Neurons were transfected with a $CSP\alpha_{WT}$ construct, in parallel with a cytosolic GFP construct, and were fixed and immunostained for Synaptophysin, as a synaptic vesicle marker (same data as in Fig 10). Black arrowheads indicate exemplary synapses not expressing the construct, white arrowheads indicate exemplary synapses expressing the construct. Scale bar: 10 µm.

(B-G) We analyzed the synaptic boutons, determining their area, perimeter, major and minor axes, as well as their intensities in the Synaptophysin channel (as a measure of the vesicle amounts) and in the GFP channel (as an indication of $CSP\alpha_{WT}$ expression). The measurements were split into “GFP-negative” and “GFP-positive” sets, which were averaged for each experiment. The graph shows the mean of n = 4 independent experiments.

Data information: All data represent the mean ± SEM. All imaging was performed with a Nikon Ti-E epifluorescence microscope. For further information, see source data.

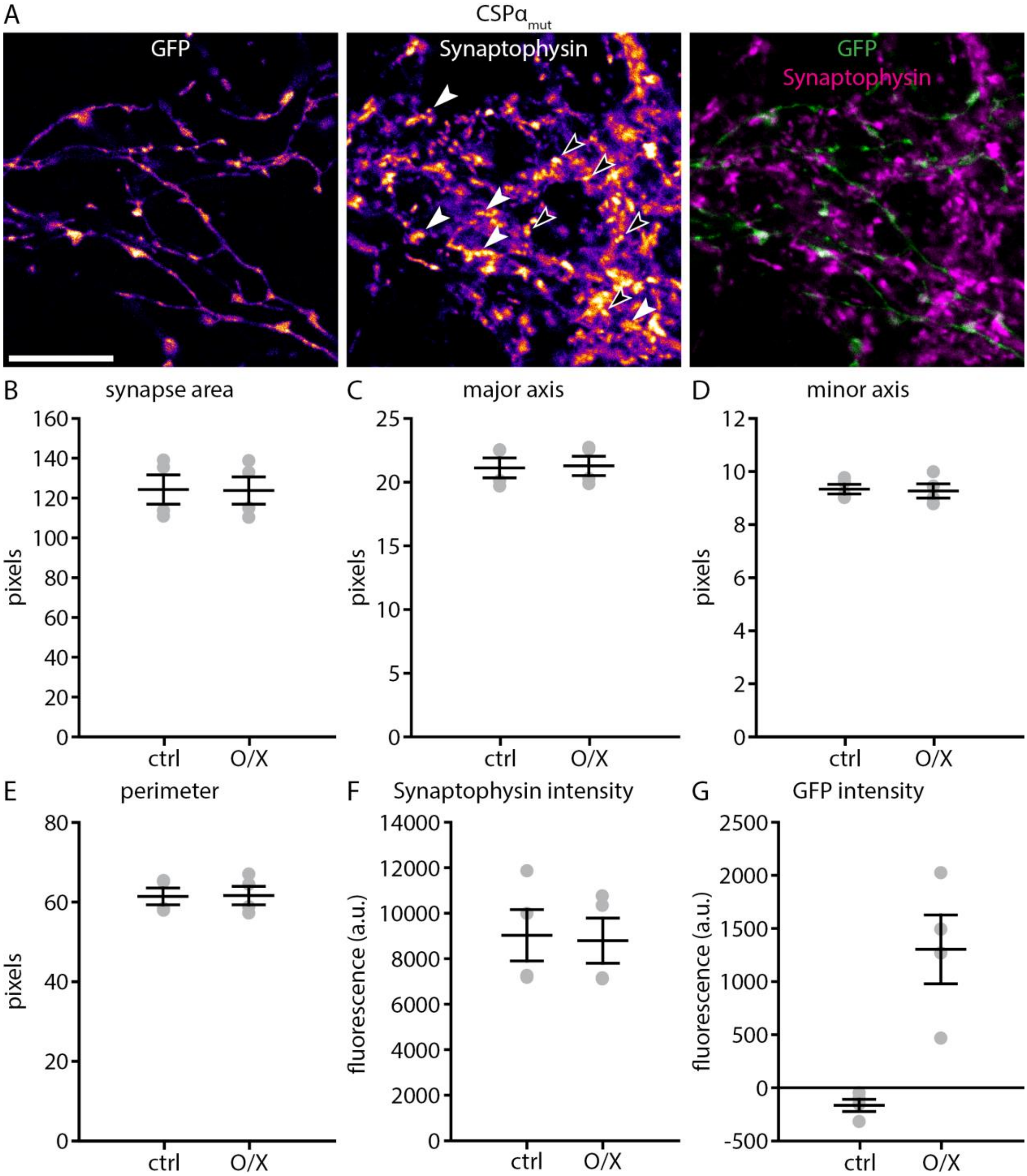

Appendix Figure S23: The expression of CSPα$_{mut}$ in neurons does not affect synaptic bouton morphology or synaptic vesicle amounts.

(A) Neurons were transfected with a CSPα$_{mut}$ construct, in parallel with a cytosolic GFP construct, and were fixed and immunostained for Synaptophysin, as a synaptic vesicle marker (same data as in Fig 10). Black arrowheads indicate exemplary synapses not expressing the construct, white arrowheads indicate exemplary synapses expressing the construct. Scale bar: 10 µm.

(B-G) We analyzed the synaptic boutons, determining their area, perimeter, major and minor axes, as well as their intensities in the Synaptophysin channel (as a measure of the vesicle amounts) and in the GFP channel (as an indication of CSP expression). The measurements were split into “GFP-negative” and “GFP-positive” sets, which were averaged for each experiment. The graph shows the mean of n = 4 independent experiments.

Data information: All data represent the mean ± SEM. All imaging was performed with a Leica SP5 confocal microscope. For further information, see source data.

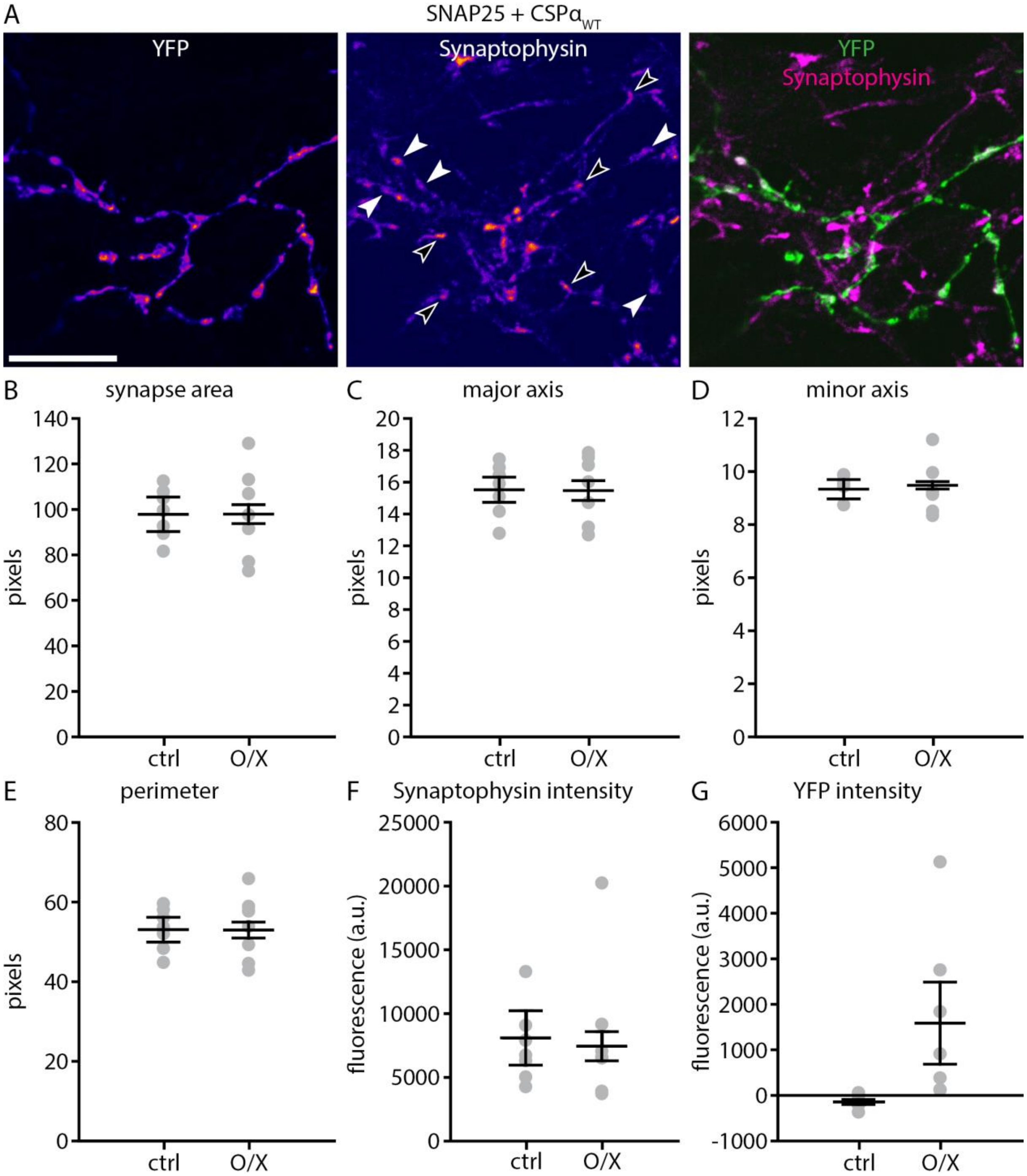

Appendix Figure S24: The expression of CSPα$_{WT}$ in neurons, along with SNAP25, does not affect synaptic bouton morphology or synaptic vesicle amounts.

(A) Neurons were transfected with a CSPα$_{WT}$ construct, in parallel with a SNAP25-YFP construct, and were fixed and immunostained for Synaptophysin, as a synaptic vesicle marker (same data as in Fig 10). Black arrowheads indicate exemplary synapses not expressing the constructs, white arrowheads indicate exemplary synapses expressing the constructs. Scale bar: 10 µm.

(B-G) We analyzed the synaptic boutons, determining their area, perimeter, major and minor axes, as well as their intensities in the Synaptophysin channel (as a measure of the vesicle amounts) and in the YFP channel (as an indication of protein expression). The measurements were split into “YFP-negative” and “YFP-positive” sets, which were averaged for each experiment. The graph shows the mean of n = 8 independent experiments.

Data information: All data represent the mean ± SEM. All imaging was performed with a Leica SP5 confocal microscope. For further information, see source data.

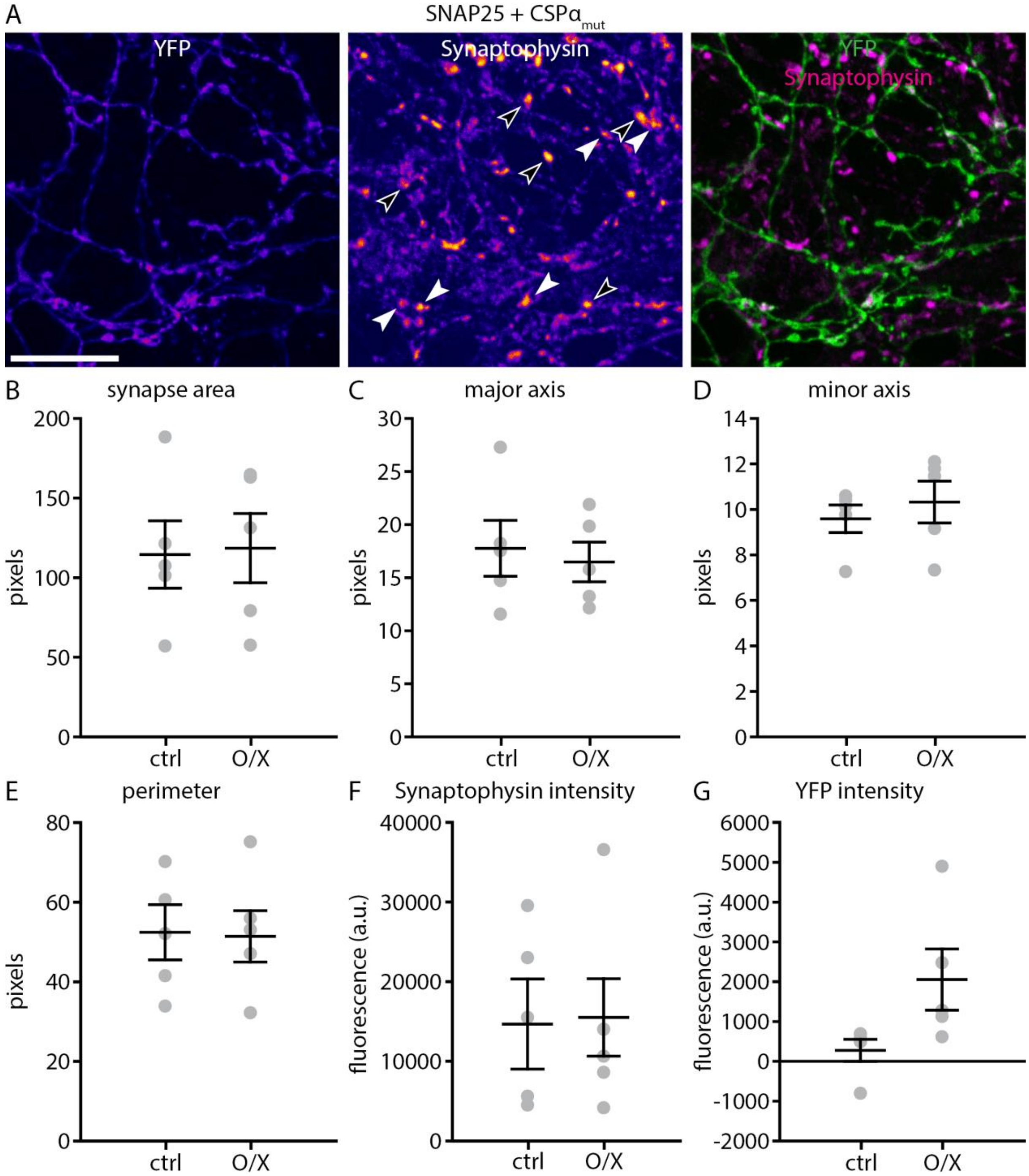

Appendix Figure S25: The expression of CSPα$_{mut}$ in neurons, along with SNAP25, does not affect synaptic bouton morphology or synaptic vesicle amounts.

(A) Neurons were transfected with a CSPα$_{mut}$ construct, in parallel with a YFP-SNAP25 construct, and were fixed and immunostained for Synaptophysin, as a synaptic vesicle marker (same data as in Fig 10). Black arrowheads indicate exemplary synapses not expressing the constructs, white arrowheads indicate exemplary synapses expressing the constructs. Scale bar: 10 µm.

(B-G) We analyzed the synaptic boutons, determining their area, perimeter, major and minor axes, as well as their intensities in the Synaptophysin channel (as a measure of the vesicle amounts) and in the YFP channel (as an indication of protein expression). The measurements were split into “YFP-negative” and “YFP-positive” sets, which were averaged for each experiment. The graph shows the mean of n = 6 independent experiments.

Data information: All data represent the mean ± SEM. All imaging was performed with a Leica SP5 confocal microscope. For further information, see source data.

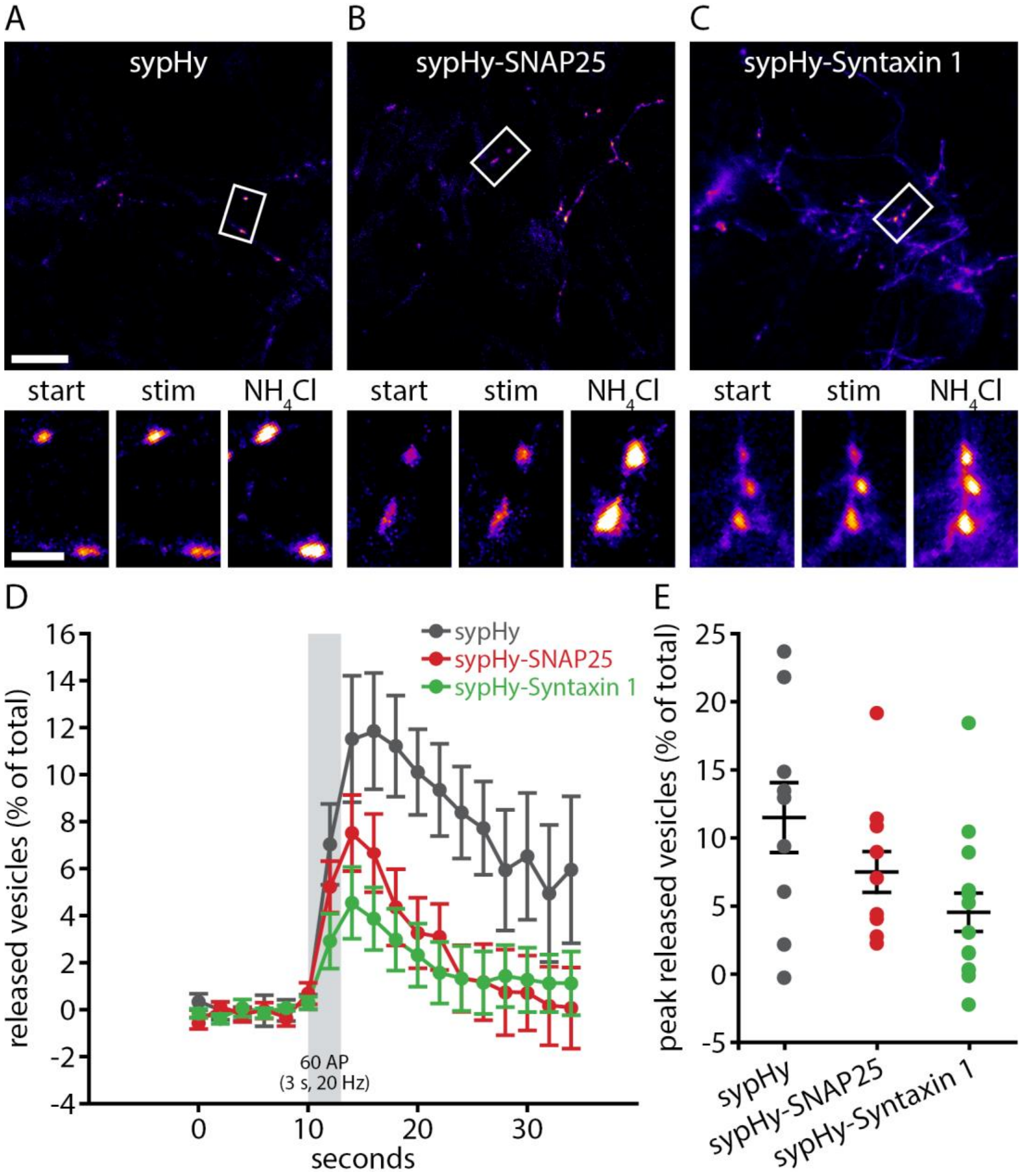

Appendix Figure S26: Vesicles containing sypHy-SNAP25 are efficient in exocytosis upon the first few stimuli of a train.

(A-C) We expressed in neurons either sypHy, a sypHy-SNAP25 construct that targets SNAP25 to synaptic vesicles, or a sypHy-Syntaxin 1 construct that targets Syntaxin 1 to the vesicles. The cells were then stimulated with 60 action potentials at 20 Hz, to trigger the release of only the primed and docked vesicles. Exemplary images are shown for both constructs, with overviews before stimulation and magnified areas before stimulation (start), at the peak of stimulation (stim), and after application of NH_4Cl. Scale bars: 20 µm (overviews) and 5 µm (magnified areas). NH_4Cl was used to reveal all sypHy moieties, to be able to represent the exocytosis response as precentage of all sypHy molecules.

(D) The cells expressing sypHy-SNAP25 displayed a less reduced response than for the longer, 600 action potential stimulation (see Fig 9).

(E) The graph shows individual data points for the peak release during stimulation for all conditions (n = 9 independent experiments for sypHy; n = 9 independent experiments for sypHy-SNAP25; n = 12 independent experiments for sypHy-Syntaxin 1; no statistically significant differences as determined via one-way ANOVA, p = 0.0705, F(2, 30) = 2.92).

Data information: All data represent the mean ± SEM. All imaging was performed with a Nikon Ti-E epifluorescence microscope, at 37°C. For further information, see source data.

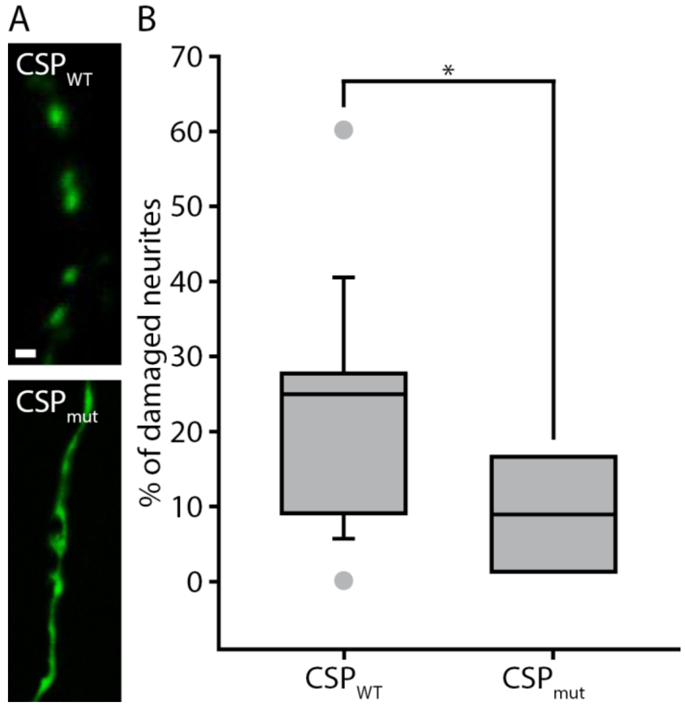

Appendix Figure S27: CSPα overexpression results in neurite degeneration.

(A) Neurons were transfected with wild-type CSPα (CSP_{WT}, top panel), or with a mutated version of CSPα unable to target to vesicles (CSP_{mut}, bottom panel). The cells were co-transfected with soluble EGFP, to detect the morphology of the neurites, in green, as shown in these panels. As shown in Fig 7, CSP_{WT} abolishes the timer mechanism and leads to the use of aged vesicles (labeling of all vesicles with Synaptotagmin 1 antibodies), while CSP_{mut} does not have any effect on vesicle use. The images show two neurites, one of which is fragmented and is composed of swollen fragments. This phenotype is typical of degenerating neurons, and was more frequent in neurons transfected with CSP_{WT}. Scale bar: 1 µm.

(B) Quantification of neurite damage in neurites overexpressing CSP_{WT} or CSP_{mut}. Overexpression of any construct only slightly impairs neuron health, but CSP_{WT} overexpression had a significantly stronger effect than CSP_{mut}, which is shown here as a control. We counted the number of neurons whose neurites were damaged, as percentage of all neurons analyzed in the respective experiments (n = 17 neurons from 4 independent experiments for CSP_{WT}, n = 8 neurons from 5 independent experiments for CSP_{mut}; *p = 0.0305, t(23) = 2.3055). All data are represented as box plots with median and upper and lower quartile boundaries, plus 1.5-times inter-quartile range (whiskers) and outliers (dots). Statistical significance was determined using the unpaired t-test.

Data information: For further information, see source data.

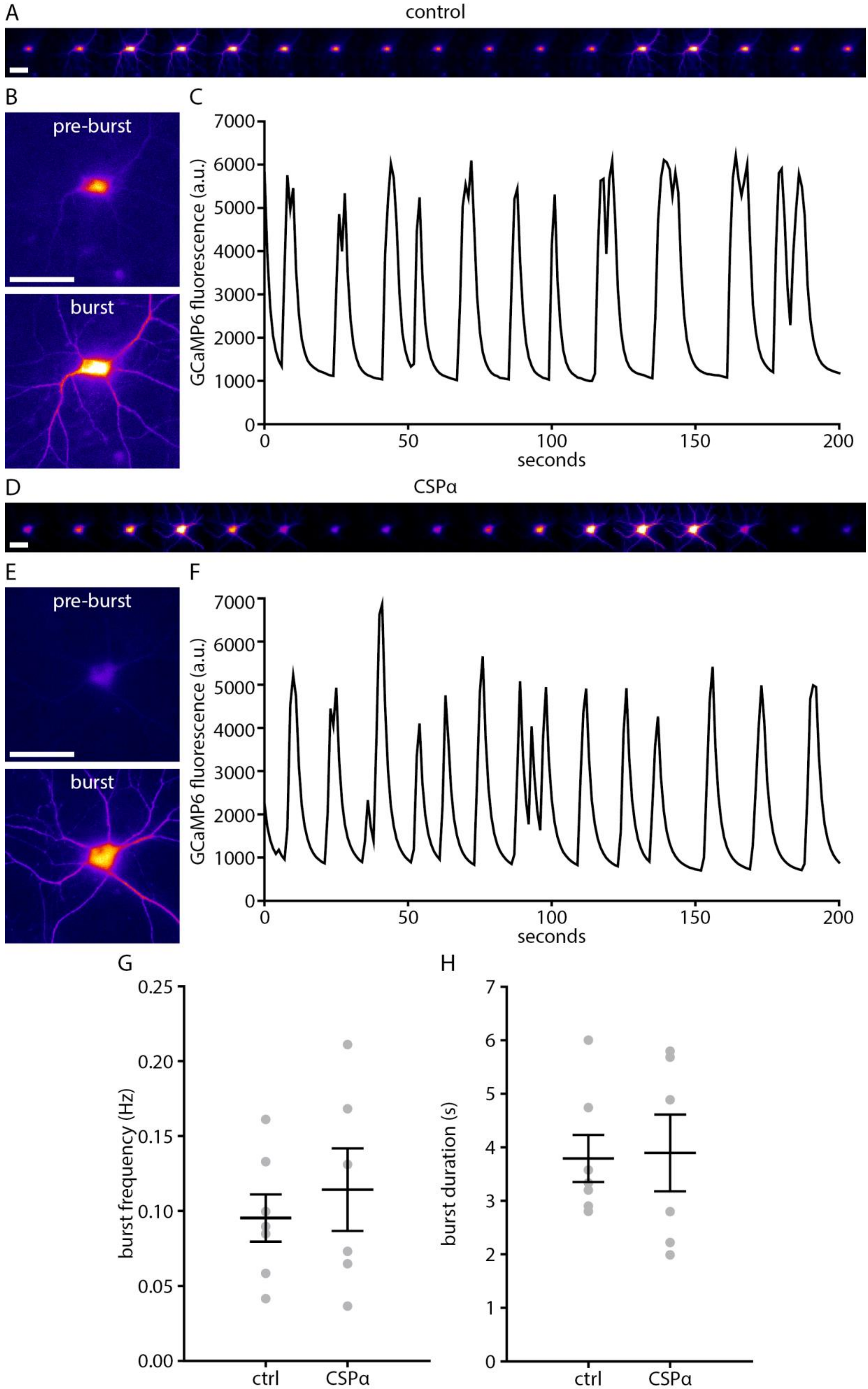

A
control
B
pre-burst
burst
C
GCaMP6 fluorescence (a.u.)
seconds
D
CSPα
E
pre-burst
burst
F
GCaMP6 fluorescence (a.u.)
seconds
G
burst frequency (Hz)
ctrl
CSPα
H
burst duration (s)
ctrl
CSPα

Appendix Figure S28: Overexpression of CSPα does not alter the Ca^{2+} signaling of the neurons.

Ca^{2+} bursts of control neurons (A,B) and neurons overexpressing CSPα (D,E) were measured with GCaMP6, as in Appendix Fig S15. Exemplary images and traces (images taken 1 s apart) are shown here. Scale bars: 50 µm.

(C,F) Exemplary fluorescence traces are shown.

(G,H) An analysis of the Ca^{2+} burst frequency (G; $p = 0.5518$, $t(11) = 0.6138$) and length (H; $p = 0.9028$, $t(11) = 0.1249$) does not show significant differences between control neurons (without overexpression, n = 7 independent experiments) and neurons overexpressing CSPα (n = 6 independent experiments). All data represent the mean ± SEM. Statistical significance was determined using unpaired t-tests.

Data information: All imaging was performed with a Nikon Ti-E epifluorescence microscope, at 37°C. For further information, see source data.

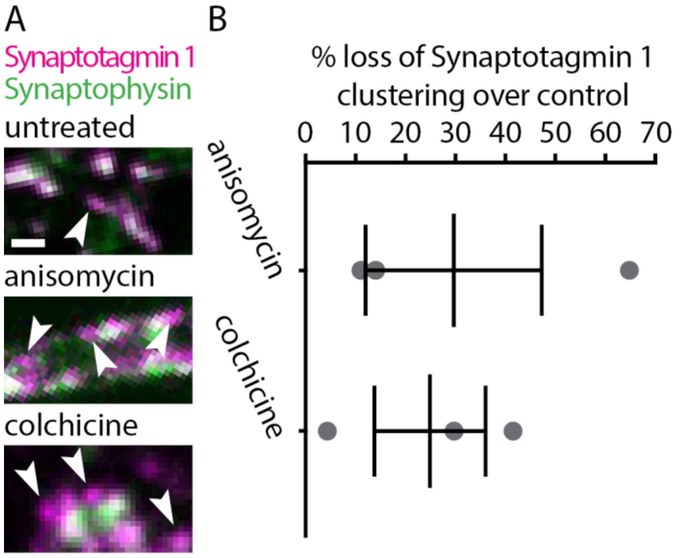

Appendix Figure S29: Aged synaptic vesicle proteins appear to be poorly endocytosed. (A) We analyzed here the co-localization of Synaptotagmin 1 and Synaptophysin molecules in the axons, in neurons from the experiment shown in Fig 5 (please see the respective figure legend for the details). In brief, the actively recycling Synaptotagmin 1 molecules were labeled in these neurons using Atto647N-conjugated antibodies, through live incubation at 37°C for 1 hour. The neurons were then returned to the incubator, and individual coverslips were treated with anisomycin, to block protein biosynthesis, or with colchicine, to inhibit axonal transport. Both treatments removed the entry of newly synthesized vesicles into the boutons (Fig 3), implying that these neurons could only recycle older vesicles for the particular time interval. We then retrieved the neurons, subjected them to a round of stimulation designed to release all recycling vesicles (20 Hz for 30s), and fixed and immunostained them for Synaptophysin, as a second marker for synaptic vesicles. We analyzed the amount of Synaptotagmin 1 molecules that did not co-localize with the Synaptophysin signals, and therefore are indicative of a loss of Synaptotagmin 1 from vesicles during vesicle recycling. We found an apparent increase for the neurons that had been treated with the drugs (see arrowheads). Scale bar: 1 µm.

(B) Quantification of increase in Synaptotagmin 1 loss of drug treated conditions over untreated control (n = 3 independent experiments per condition). All data represent the mean ± SEM.

Data information: All experiments were imaged using a Leica SP5 confocal microscope. For further information, see source data.

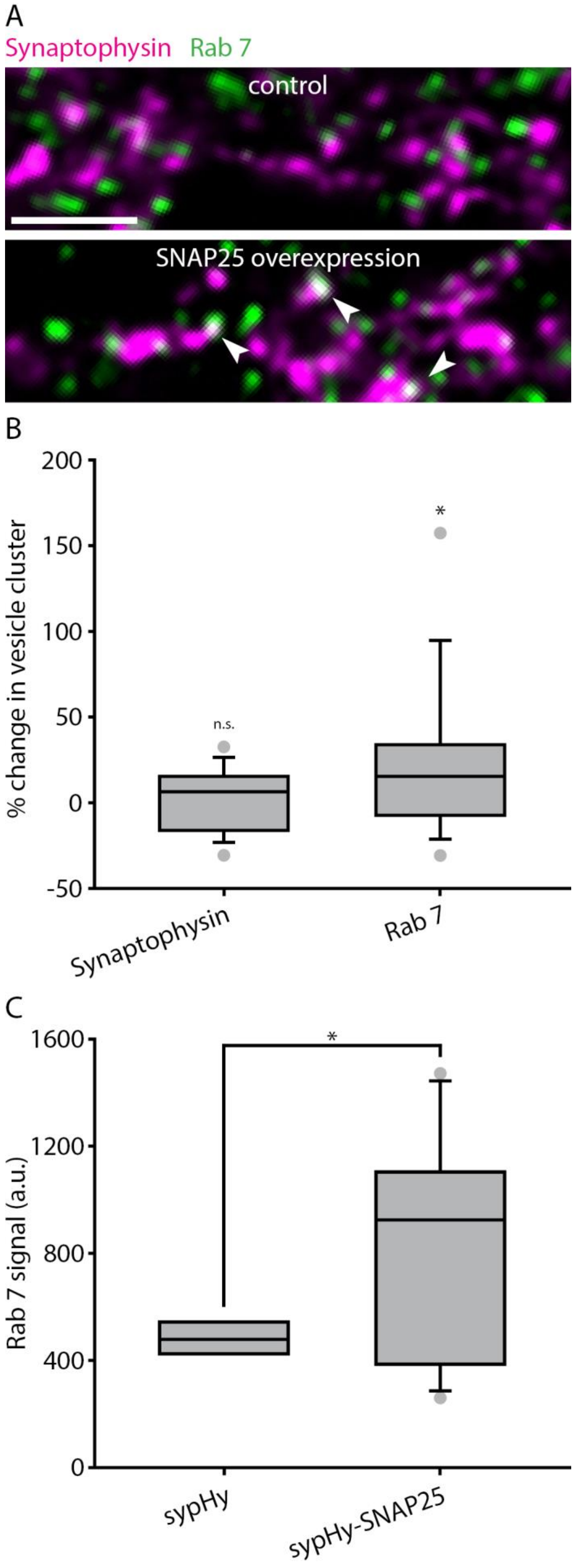
A
Synaptophysin Rab 7
control
SNAP25 overexpression
B
% change in vesicle cluster
200
150
100
50
0
-50
n.s.
*
Synaptophysin
Rab 7
C
Rab 7 signal (a.u.)
1600
1200
800
400
0
*
sypHy
sypHy-SNAP25

Appendix Figure S30: SNAP25 overexpression targets synaptic vesicles to recycling endosomes.

(A) Neurons were transfected with wild-type SNAP25, and were then fixed and co-immunostained for Synaptophysin, as a vesicle marker, and for Rab 7, as a recycling endosome marker (right panel). As a control, the immunostaining was performed in non-transfected neurons. Note that SNAP25 is not shown in the panels. Scale bar: 2 µm.

(B) We quantified the co-localization of Synaptophysin with Rab 7, and we expressed it as a percentage change from the co-localization in normal, non-transfected neurons. As a control, we also analyzed the amount of vesicles, measured by Synaptophysin immunostaining, and we also expressed it as a percentage change from the amount of Synaptophysin in normal, non-transfected neurons. The latter does not change significantly (n.s., $p = 0.3929$, $t(34) = 0.8654$). However, the presence of Rab 7 within vesicle clusters increases significantly ($n = 18$ transfected neurons from 10 independent experiments; *$p = 0.0296$, $t(34) = 2.2710$).

(C) We quantified the co-localization of Synaptophysin with Rab 7 in cells overexpressing either sypHy or sypHy-SNAP25. As sypHy here defines the vesicle clusters, no immunostaining for Synaptophysin was necessary, and the experiment could be quantified by simply measuring the intensity of Rab 7 immunostaining within the sypHy spots. $n = 8$ cells from 4 independent experiments for sypHy, and $n = 14$ cells from 4 independent experiments for sypHy-SNAP25; *$p = 0.0415$, $t(20) = 2.1786$, for sypHy-SNAP25.

Data information: All data are represented as box plots with median and upper and lower quartile boundaries, plus 1.5-times inter-quartile range (whiskers) and outliers (dots). Statistical significance was evaluated using unpaired t-tests. All experiments were imaged using a Leica SP5 confocal microscope. For further information, see source data.

A

protein	copy number	lifetime
Synaptophysin	31	4.1 days
Syntaxin 1	6	5.4 days
SNAP25	2	2.3 days
Synaptotagmin 1	15	2.7 days
Synaptogyrin 1	2	5.2 days
SV2a	10 (SV2a/b)	3.8 days
SV2b	10 (SV2a/b)	3.4 days
vATPase a1	1	3.6 days
total	67	3.8 ± 1.1 days

B

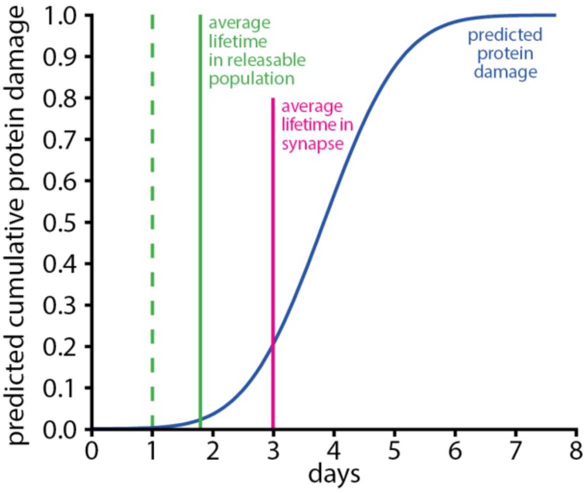

Appendix Figure S31: Synaptic vesicle protein entry in the inactive pool precedes the accumulation of damaged synaptic vesicle proteins, as predicted from their lifetimes.
(A) Table of protein copies per vesicle (Wilhelm *et al*, 2014) and protein lifetimes (Cohen *et al*, 2013) in culture, for the major synaptic vesicle proteins. The average lifetime of the vesicle protein, derived from weighting individual protein lifetimes by protein copy number, and the associated standard deviation are shown in the line "total".
(B) Cumulative Gaussian probability plot of protein damage, assuming that the protein lifetimes end when the proteins become damaged (i.e., assuming that the damaged proteins are degraded within a few hours after becoming damaged). The time point of vesicle precursors entering the synapse was placed at 24 hours (the left-most green dashed line), based on the speed with which GFP-tagged synaptic vesicle proteins such as Synaptophysin, Synaptobrevin/VAMP2 or Synaptotagmin 1 are expressed in synapses in our cultures. Based on our data, we hypothesize that these vesicles enter quickly into the releasable population. The green line indicates the time point at which synaptic vesicles leave the releasable population, counting from the time they entered the synapse, and enter the inactive population (Appendix Fig S12C). The vesicles become inactive just before the accumulation of substantial damage: the blue curve indicating predicted protein damage is close to zero at this time point. The pink line indicates the time point at which synaptic vesicles leave the synapse, and proceed towards degradation in the cell body. Note that this time point, derived from lifetime measurements of tagged Synaptotagmin 1 in Fig 1B,D added to our estimate of protein trafficking to the synapse (see above), is very close to previously measured lifetimes of Synaptotagmin 1 (Cohen *et al*, 2013) (see table in (A)).

MATERIAL AND METHODS

Hippocampal cultures. Neuronal hippocampal cultures were obtained from dissociated hippocampi of new-born rats (Banker & Cowan, 1977; Kaech & Banker, 2006). In brief, brains were extracted from the skulls of two days-old rat pups, and the hippocampi were isolated under a dissection microscope. Following washes with HBSS (Invitrogen, Waltham, MA, USA) to remove tissue debris, the hippocampi were incubated for 1 h in enzyme solution (10 ml DMEM, 2 mg cysteine, 100 mM $CaCl_2$, 50 mM EDTA, and 25 U papain, equilibrated with carbogen for 10 min, and sterile filtered). Before mechanical dissociation, cells were washed thoroughly with HBSS and were incubated for 15 min in inactivating solution (2 mg albumin and 2 mg trypsin inhibitor in 10 ml FCS-containing DMEM medium). Before seeding, coverslips were treated with nitric acid, washed thoroughly with double distilled water, were sterilized and were coated overnight with 1 mg/ml PLL. After coating, coverslips were washed thoroughly with sterile water, and were incubated with plating medium (MEM supplemented with 10% horse serum, 3.3 mM glucose, and 2 mM glutamine). Following dissection, neurons were plated at a concentration of ~30,000/cm^2 and were left to adhere for 1-4 h at 37°C in a 5% CO_2 cell-incubator. After adhesion, the medium was changed to Neurobasal-A medium (Gibco, Life Technologies, Carlsbad, CA, USA) containing 1:50 B27 supplement (Gibco) and 1:100 GlutaMAX (Gibco). To avoid glial proliferation, 5-fluoro-2'-deoxyuridine was added to the cells after ~2-3 d in culture. Neurons were kept in culture at 37°C and 5% CO_2 for 14-21 days before use.

Transfections. Transfections were performed with a standard calcium phosphate kit (Promega, Fitchburg, WI, USA), using a procedure slightly modified from the manufacturer's protocol. To minimize toxicity, neurons were pre-incubated for 30 min in 37°C DMEM complemented with 10 mM $MgCl_2$ at pH 7.5 (fresh-DMEM). Per 18 mm cover slip, 50 µl of transfection mix were applied, containing 2 µg of plasmid DNA for single transfections, or 1.5 µg of each plasmid for double transfections, as well as 5 µl $CaCl_2$, 25 µl HBS, and H_2O to 50 µl; HBS was applied last, followed by vigorous vortexing. Prior to application to the cultured neurons, the transfection mix was incubated at room temperature for 15 min, to allow formation of the calcium phosphate precipitate. The cultured neurons were incubated with the transfection mix for 15 min at 37°C and 5% CO_2. After 3 washes with fresh-DMEM, the cultured neurons were placed back into their original culture medium and were incubated for 4 days at 37°C and 5% CO_2, to allow for sufficient protein expression. Alternatively, lipofection was performed using the Lipofectamine 2000 kit (Thermo Fisher Scientific, Waltham, MA, USA) was used. Briefly, 1 µg of DNA was used per coverslip of a 12-well plate. The DNA was prepared in a total volume of 25 µl Opti-MEM per well, incubated for 5 min on room-temperature before adding 25 µl total volume of Opti-MEM with 1 µl lipofectamine solution. The mixture was then incubated for 20 min before adding it to neurons incubated for 20 min in 1 ml fresh-DMEM (see above). After 25 min incubation at at 37°C and 5% CO_2, the neurons were washed 2-3 times with fresh-DMEM and placed back into their original culture medium for 3-4 days before experiments.

Cloning. GCAMP6s, sypHy, and mOrange2 were obtained through Addgene (Cambridge, MA, USA; plasmid numbers 40753, 24478, and 54650, respectively). The chimeric mOr2-sypHy indicator for synaptic vesicle recycling was created by substituting the superecliptic GFP from the original sypHy construct with the pH-sensitive mOrange2 fluorescent protein. Briefly, the supercliptic GFP was excised by AgeI restriction, and mOrange2 was PCR-amplified to add

the AgeI sites, and was finally ligated between the third and the forth TM domain of rat Synaptophysin 1 (NM_012664). Positive clones were screened by double AgeI/SalI digestion and correct orientation was confirmed by sequencing. YFP-SNAP25 (kind gift from R. Jahn) was constructed and expressed via cloning SNAP25 into the pEYFP-C1 vector (Clontech, Takara Bio Inc., Otsu, Japan) via the SacI and ApaI restriction sites. Syntaxin 1-YFP was obtained as described previously (Vreja *et al*, 2015). Wild type CSPα$_{WT}$ and mutated CSPα$_{mut}$, in which all cysteine residues of the cysteine string had been changed to serine (Sharma *et al*, 2012), were obtained from GeneArt (Invitrogen, Waltham, MA, USA) and were cloned via the NheI and BamHI restriction sites into a pIRES2 AcGFP backbone vector (Clontech, Takara Bio Inc., Otsu, Japan) in which GFP had been excised and replaced with mCherry amplified from the pmCherry-N1 vector (Clontech, Takara Bio Inc., Otsu, Japan), with the addition of BstXI and NotI restriction sites. The VAMP2-TEV-SNAP construct was cloned by inserting the SNAP tag domain into a VAMP2-TEV construct in the pEGFP-N1 backbone ordered from GeneArt (Invitrogen, Waltham, MA, USA) via the NheI and AgeI restriction sites. The sypHy-SNAP25 construct was created by inserting SNAP25 at the C-terminus of the sypHy construct described above, using the HindIII and the XbaI restriction sites. The sypHy-Syntaxin 1 construct was synthesized through GenScript (Piscataway Township, NJ, USA) using the sypHy mOrange2 sequence described above and the canonical rat sequence for Syntaxin 1 as retrieved from NCBI (Bethesda, MA, USA), but without the membrane-integration domain and without the intra-vesicular domain, to prevent mistargeting, separated by a custom linker designed to provide flexibility and avoid interference of the fused protein domains (TGGGSGGGSGGGSAAA).

Stimulation and inhibition of activity. To inhibit lysosomal degradation (Appendix Fig S9C), leupeptin (Sigma-Aldrich, St. Louis, MO, USA) was applied at a concentration of 100 µM. To block neuronal activity, tetrodotoxin (TTX; Cayman Chemical Company, Ann Arbor, MI, USA) was applied at 0.5-1 µM alone or with 10 µM CNQX (Tocris Bioscience, Bristol, UK; Abcam, Cambridge, UK) and 50 µM AP5 (Tocris Bioscience, Bristol, UK; Abcam, Cambridge, UK). Electrical stimulation of neurons was achieved with field pulses at 10 Hz for 60 s or at 20 Hz for 30 s at 100 mA with a 385 Stimulus Isolator and an A310 Accupulser Stimulator (both from World Precision Instruments, Sarasota, FL, USA), using a custom-made platinum plate field stimulator (8-mm distance between the plates). To differentiate between releasable and inactive vesicles using CypHer5E-coupled antibodies, cells were imaged before stimulation and after stimulation in 0.5 µM bafilomycin (Sigma-Aldrich, St. Louis, MO, USA). To increase neuronal activity, 20 µM bicuculline (Sigma-Aldrich) or 8 mM Ca^{2+} were applied for 12 h.

Fused vesicle immunostaining. For the experiment described in Appendix Fig S1, the surface Synaptotagmin 1 epitopes were blocked by incubation with unconjugated 604.2 antibodies, for 10 minutes, in the absence of Ca^{2+} (to inhibit vesicle recycling), at room temperature. The neurons were afterwards incubated for 6 minutes under limited depolarization (using 15 mM KCl), at 4°C, to inhibit endocytosis, but not exocytosis (Hoopmann *et al*, 2010). Fluorescently conjugated Synaptotagmin 1 antibodies (Atto647N) were added during the 6 minutes, together with a rabbit polyclonal serum which recognizes a luminal epitope of Synaptophysin (G96) (Hoopmann et al. 2010). After fixation, the Synaptophysin antibodies were revealed by anti-rabbit secondaries conjugated to Atto590, and the cultures were embedded and imaged by two-color STED microscopy, as described (Hoopmann *et al*, 2010). This experiment enables the quantitative analysis of the loss of Synaptotagmin 1 molecules from co-labelled Synaptotagmin 1 and Synaptophysin vesicle patches. However,

Synaptophysin molecules that do not co-localize with Synaptotagmin 1 may represent vesicles that were blocked with the unconjugated Synaptotagmin 1 antibody, and therefore cannot be considered for an analysis of the diffusion of Synaptophysin from the co-labelled molecule patches.

Embedding and thin-sectioning. For 50 nm ultrathin-sectioning, neurons were embedded in melamine resin. The resin hardener was prepared by dissolving 48 mg p-toluenesulfonic acid monohydrate (Sigma-Aldrich, St. Louis, MO, USA) in 0.576-ml distilled water. 1.344 g 2,4,6-Tris[bis(methoxymethyl)amino]-1,3,5-triazine (melamine) was added, and the mixture was agitated on a horizontal shaker at 250 rpm until the melamine was completely dissolved. After immunostaining (see above), a BEEM capsule (BEEM Inc., West Chester, PA, USA), whose bottom had previously been cut, was placed with the opening down on the cover slip. 200 µl of freshly prepared melamine was poured inside the BEEM capsule, covering the cell layer completely. The mounted sample was placed in a box containing silica beads for removing moisture, and was left overnight at room temperature, to allow penetration of melamine into the cells. The box was then heated to 40°C for 24 h to harden the melamine. The BEEM capsule was then filled with Epon resin (EpoFix kit; Struers, Willich, Germany) and was heated to 60°C for at least 48 h. The melamine around the region of interest was trimmed away with a razor blade. Melamine blocks were then cut into 50 nm thin sections with an EM UC6 ultramicrotome (Leica, Wetzlar, Germany). Sections were dried on a coverslip and were embedded in Mowiol, for two-color STED imaging. For nanoSIMS imaging, cells were embedded in LR White instead, to minimize background signal from amines in melamine. Briefly, samples were dehydrated after immunostaining by washing 5 min with 30% ethanol in ddH_2O, followed by 3x 5 min in 50% ethanol. The samples were then incubated with LR White medium grade acrylic resin (London Resin Company, London, UK) in 50% ethanol (1:1 mixture), followed by 60 min incubation in pure LR White. For the final embedding step, we used LR White with 1:20 LR White accelerator (London Resin Company, London, UK) for 15 min on room temperature followed by 90 min at 40°C. We then cut 200 nm thin sections, as described above, to provide sufficient sample volume for pre-ionization implantation during nanoSIMS imaging, and placed them on silicon wafers for imaging.

Data Analysis. Fluorescence images were analyzed with custom-written MATLAB (MathWorks, Natick, MA, USA) routines. The fluorescence levels of Synaptotagmin 1 staining were normalized in all experiments involving confocal imaging of live-tagged Synaptotagmin 1 with unconjugated antibodies, or with antibodies conjugated to Atto647N, to the levels of Synaptophysin in the respective boutons. These were obtained from co-immunostaining experiments. The normalization was performed to correct for variations in the size of the total vesicle pool in different boutons, and between cultures. For display purposes, the images from Fig 3C, Fig 8A,B, Fig 10A, Appendix Fig S6A,C, Appendix Fig S16A,C, Appendix Fig S17A,B, and Appendix Fig S30A were deconvolved using Huygens Essential 4.4 (Scientific Volume Imaging, Hilversum, Netherlands), based on in-built algorithms that were adjusted to the imaging parameters from the particular images. Automated analyses of synapse morphology (e.g. Appendix Figs S20-25) were performed as follows. The Synaptophysin immunostaining images were subjected to a band pass filter, to remove noise, and the signals remaining, higher than an empirically determined threshold (typically mean + 1 standard deviation of the filtered image fluorescence) were turned into individual regions of interest. All regions of interest larger than 25 pixels in area were then analyzed, using the Regionprops MATLAB function, targeting the areas, major and minor axes, and the perimeter lengths; their intensity in the original raw

data image was also determined, as well as their intensities in other channels, such as the GFP channel. The average values were determined for each neuron analyzed.

Prediction of protein damage from protein lifetimes. We used a published list of synaptic protein lifetimes (Cohen *et al*, 2013) to approximate accumulation of protein damage on the synaptic vesicle over time (Appendix Fig S31). We considered all synaptic vesicle proteins whose lifetimes have been measured (Appendix Fig S31A). The average lifetime of a synaptic vesicle protein is 3.8 days, with a standard deviation of 1.1 days (Appendix Fig S31A). If we assume that each protein is degraded as it becomes damaged, one can derive from these two parameters a cumulative normal distribution, or probability density function, that predicts the accumulation of protein damage on the average vesicle (Appendix Fig S31B). On the same graph we marked the approximate time points at which the synaptic vesicle enters into the releasable population, leaves this population to enter the inactive population, and finally the time point at which the vesicle leaves the synapse for degradation in the cell body (Appendix Fig S12).

Quantitative model of the synaptic vesicle life cycle. We combined the conclusions we drew from our own data with information from the literature to re-construct a least-complexity model of the synaptic vesicle life cycle (Fig 7). The kinetic relationships of the model derived from these purely qualitative considerations were constructed in COPASI (Pedro Mendes, University of Manchester) and were fed with variables known from the literature or directly derived from our data. Using Mathematica (Wolfram Research, Champaign, IL, USA), we obtained the values for not experimentally determined kinetic rates; the simplicity of the model allowed us to derive these values directly from the known variables. For simplicity, we use below the term "vesicle" throughout the explanation, although one may want to use the concept of "meta-stable synaptic vesicle protein assemblies that are assembled during endocytosis to form synaptic vesicles" for the stages between exocytosis and endocytosis.

We assume a very simple model where we have a pool of active and a pool of inactive vesicles as sketched in Fig 7. A and B are the sizes of the two pools. Active vesicles are created at the biogenesis rate k_b and subsequently inactivated at the inactivation rate k_i. Finally with the degradation rate k_d the inactive vesicles are degraded. The dynamics can be described by the following set of coupled differential equations:

$$\frac{dA}{dt} = k_b - k_i \cdot A$$
$$\frac{dB}{dt} = k_i \cdot A - k_d \cdot B$$

Note that k_b is an absolute rate, whereas k_i and k_d are rates per vesicle.
In an equilibrium situation we know that A and B as well as $A + B$ must be constant in size, i.e. their derivatives have to be 0. From the two following equations

$$\frac{d(A+B)}{dt} = k_b - k_d \cdot B = 0$$
$$\frac{d(A)}{dt} = k_b - k_i \cdot A = 0$$

we can deduce that

$$k_d = k_b/B_0$$
$$k_i = k_b/A_0$$

where B_0 is the constant size of the pool of inactive vesicles and A_0 is the constant size of the pool of active vesicles.

If we now introduce a labeling of synaptic vesicles, as in the experiment in Appendix Fig S12, we need to take into account two additional pools, namely the pool of labeled active (AL) and of labeled inactive (BL) vesicles. We also note that $A_0 = A + AL$, and $B_0 = B + BL$,
The rates for inactivation and degradation are the same as for the unlabeled pools, but there is no biogenesis of labeled active vesicles. Hence the dynamics of the system with labeling are described by the following set of equations:

$$\frac{dA}{dt} = k_b - k_i \cdot A$$
$$\frac{dAL}{dt} = -k_i \cdot AL$$
$$\frac{dB}{dt} = k_i \cdot A - k_d \cdot B$$
$$\frac{dBL}{dt} = k_i \cdot AL - k_d \cdot BL$$

If we label all active vesicles instantaneously at $t = 0$ this corresponds to the following initial conditions:

$$A(0) = 0, AL(0) = A_0, B(0) = B_0, BL(0) = 0$$

Using these initial conditions and the equations for k_i and k_d we can solve for the temporal evolution of the pool sizes and find e.g. for the labeled active vesicles

$$AL(t) = A_0 \cdot e^{-k_b \cdot t / A_0}$$

and for the total number of labeled vesicles

$$TL(t) = AL(t) + BL(t) = \frac{A_0}{A_0 - B_0} \cdot \left(A_0 \cdot e^{-k_b \cdot t / A_0} - B_0 \cdot e^{-k_b \cdot t / B_0}\right)$$

Knowing A_0, B_0 and k_b we can thus predict the number of labeled active vesicles as well as the total number of labeled vesicles. We use the biogenesis rate $k_b = 6.8\, h^{-1}$ (estimated by calculating how often a new vesicle would need to arrive at the synapse to maintain a constant rate of activity, assuming an average of 210 release events per synaptic vesicle lifetime, as calculated above) and the ratio of the pool sizes (as shown in Appendix Fig S7E: $B_0/A_0 = 1.1$) as well as the absolute number of vesicles derived from Schikorski and Stevens, 1997, by taking into account the presence of active vesicles on the cell surface as shown in Appendix Fig S7E: $A_0 + B_0 = 260$). Thus $A_0 = 124$ and $B_0 = 136$.

Theoretical prediction and experimental data agree reasonably well (Fi. 7b). Note that there are no free parameters involved to make the model fit to the data, but that the predictions are based on the model and on the independently derived experimental parameters A_0, B_0 and k_b.

The inactivation and degradation rates are the inverse of the respective lifetimes, i.e. the lifetime of active vesicles before inactivation is $\tau_i = {}^{1}/_{k_i} = {}^{A_0}/_{k_0} = 18.2\ h$, corresponding to a half-life

$$t_{i,{}^{1}/_{2}} = \tau_i \cdot \ln(2) = 12.6\ h.$$

The half-life of the inactive vesicles before degradation can be calculated accordingly:

$$t_{d,{}^{1}/_{2}} = 13.9\ h$$

The probability density distribution for an individual vesicle to get inactivated at a certain time t follows an exponential distribution, which is the derivative of $AL(t)$, with the integral normalized to 1,

$$f(t) = \frac{1}{\tau_i}\ e^{-t/\tau_i}$$

As a consequence an individual active vesicle “lives” on average $\tau_i = 18.2\ h$ before inactivation and an inactive vesicle “lives” on average $\tau_d = 20.1\ h$ before degradation.
We can also calculate the distribution of the total lifetime of vesicles, i.e. the time from biogenesis to degradation, which is the negative of the derivative of the total number of vesicles:

$$g(t) \sim \frac{dTL}{dt} \sim \frac{k_b}{A_0 - B_0} \cdot \left(e^{-k_b \cdot t/A_0} - e^{-k_b \cdot t/B_0}\right)$$

Fig 7E shows a histogram of the total lifetimes. The expectation value of this distribution (i.e. the average time between biogenesis and degradation) is

$$\langle g(t) \rangle = \frac{A_0 + B_0}{k_b} = \frac{1}{k_i} + \frac{1}{k_d} = 38.3\ h$$

Knowing the distribution of inactivation times (Fig 7C) we can also calculate how often active vesicles are used before being inactivated. From experiments we know that synapses are activated at a rate of $0.09\ Hz$ (Appendix Fig S15C) and that on average on every activation event $0.45 \cdot 7.5\ \% = 3.375\%$ (see above) of all active vesicles are used. Thus the average usage rate of an active vesicle is $\sim 11\ h^{-1}$. Since this rate is large as compared to the inactivation rate the distribution of how often a vesicle is used is determined by the distribution of inactivation times, i.e. it follows the same distribution but scaled with the usage rate as shown in Fig 7F. Thus on average a vesicle is used ~ 200 times before being inactivated. This matches very well with the prediction derived from calculations made from the experimental parameters (see above).

APPENDIX REFERNCES

Banker GA & Cowan WM (1977) Rat hippocampal neurons in dispersed cell culture. *Brain Res.* **126:** 397–425

Cohen LD, Zuchman R, Sorokina O, Müller A, Dieterich DC, Armstrong JD, Ziv T & Ziv NE (2013) Metabolic turnover of synaptic proteins: kinetics, interdependencies and implications for synaptic maintenance. *PLoS One* **8:** e63191

Hoopmann P, Punge A, Barysch S V, Westphal V, Bückers J, Opazo F, Bethani I, Lauterbach M a, Hell SW & Rizzoli SO (2010) Endosomal sorting of readily releasable synaptic vesicles. *Proc. Natl. Acad. Sci. U. S. A.* **107:** 19055–60

Kaech S & Banker G (2006) Culturing hippocampal neurons. *Nat. Protoc.* **1:** 2406–2415 Available at: http://dx.doi.org/10.1038/nprot.2006.356

Saka SK, Vogts A, Kröhnert K, Hillion F, Rizzoli SO & Wessels JT (2014) Correlated optical and isotopic nanoscopy. *Nat. Commun.* **5:** 3664 Available at: http://www.pubmedcentral.nih.gov/articlerender.fcgi?artid=3996535&tool=pmcentrez&rendertype=abstract

Schikorski T & Stevens CF (1997) Quantitative ultrastructural analysis of hippocampal excitatory synapses. *J. Neurosci.* **17:** 5858–5867

Sharma M, Burré J, Bronk P, Zhang Y, Xu W & Südhof TC (2012) CSPα knockout causes neurodegeneration by impairing SNAP-25 function. *EMBO J.* **31:** 829–41

Vreja IC, Niki I, Go F, Bates M, Kro K, Outeiro TF, Hell SW, Lemke EA & Rizzoli SO (2015) Super-resolution Microscopy of Clickable Amino Acids Reveals the Effects of Fluorescent Protein Tagging on Protein Assemblies. *ACS Nano* **9:** 11034–11041

Wilhelm BG, Groemer TW & Rizzoli SO (2010) The same synaptic vesicles drive active and spontaneous release. *Nat. Neurosci.* **13:** 1454–1456 Available at: http://dx.doi.org/10.1038/nn.2690%5Cnpapers3://publication/uuid/60C550EE-9AB9-4172-8FA6-0892A78DD29A

Wilhelm BG, Mandad S, Truckenbrodt S, Kröhnert K, Schäfer C, Rammner B, Koo SJ, Claßen GA, Krauss M, Haucke V, Urlaub H & Rizzoli SO (2014) Composition of isolated synaptic boutons reveals the amounts of vesicle trafficking proteins. *Science (80-.).* **344:** 1023–1028
