# Supplementary material for: Newly produced synaptic vesicle proteins are preferentially used in synaptic transmission
Source: EMBO J. 2018 Jun 27;37(15):e98044. doi: 10.15252/embj.201798044 (PMC6068464; doi:10.15252/embj.201798044)
Supplement: Supplementary file 2 — Source Data for Appendix [file EMBJ-37-e98044-s011.zip › 180518_Appendix_SourceData/180518_Table10_FigS1.docx]

**Summary Tables of Experiments**

The following tables provide a short summary of the experiments presented in this manuscript, to aid as a quick reference. Each table is preceded by a short description of the experiment, its experimental question, and the result. Additional details (e.g. for common procedures such as immunostainings, company details and order numbers, construct cloning, or on the microscope setups referenced here) can be found in the Online Methods. All experimental applications and procedures are explained in the respective figure legends. This summary is only meant as an additional source of information. For example, multiple figures rely on similar procedures, and therefore the procedures are only explained once, in the first figure where the procedure is used, and are then no longer explained in the subsequent figure legends. However, they are explained here in detail, for each set of experiments, so that one can immediately obtain the necessary details.

**Table 1: Basic time course of synaptic vesicle protein degradation (relates to Fig 1).** In this set of experiments we determined the degradation times of synaptic vesicles, tagged at the synapse during recycling, using antibodies directed against the lumenal domain of two synaptic vesicle proteins, Synaptotagmin 1 and VGAT. The antibodies were applied to live primary hippocampal neuron cultures. We determined that the half-life of degradation is ~2 days for both proteins. To ensure that synaptic signals were exclusively analyzed, only Synaptotagmin 1 or VGAT signals co-localizing with an immunostaining for Synaptophysin (an optimal presynaptic marker) were measured.

| Figure | Fig 1b-e |
| --- | --- |
| number of experiments | Synaptotagmin 1 live tagging: 3 (day 0), 3 (day 1), 2 (day 2), 3 (day 4), 3 (day 7), 2 (day 10) independent experiments, >10 neurons imaged per experiment  VGAT live tagging: 4 (day 0), 4 (day 1), 2 (day 2), 4 (day 4), 4 (day 7), 3 (day 10) independent experiments, >10 neurons imaged per experiment. |
| antibodies used | Synaptotagmin 1: Synaptic Systems, 105 311AT, clone 604.2, lumenal domain, conjugated to Atto647N  VGAT: Synaptic Systems, 131 103CpH, lumenal domain, conjugated to CypHer5E  co-immunostaining after fixation: Synaptic Systems, 101 004, Synaptophysin |
| antibody live tagging | Synaptotagmin 1 or VGAT antibody was applied (1:120 from 1 mg/ml stock), to live primary hippocampal neurons, in their own culture medium, for 1 h at 37°C in a cell culture incubator. The antibody was then washed off with ice-cold Tyrode’s solution (3-times on/off), and the cultures were maintained in their own culture medium until processing for their respective time point. |
| description of time course | Live tagging of releasing synaptic vesicles was performed as described in the previous table row, right before processing for the initial time point (day 0). Separate cultures for each time point (day 0, day 1, day 2, day 4, day 7, day 10) were pulsed in parallel and were maintained in incubator until processing. |
| stimulation paradigm | No external stimulation, only intrinsic network activity of primary hippocampal cultures during live antibody tagging and time course |
| fixation and processing | Synaptotagmin 1: 4% PFA (15 min 4°C, 30 min on room temperature), standard immunostaining for Synaptophysin to detect synapses, embedded in Mowiol  VGAT: methanol (20 min, -20°C), no additional immunostaining, application of pH 5.5 TES buffered solution to activate CypHer5E during imaging |
| imaging setup | Synaptotagmin 1: Leica TCS SP5 (confocal mode), 63x apochromat oil immersion objective  VGAT: Nikon Ti-E, 60x apochromat oil immersion objective |

**Table 2: Releasability of ageing synaptic vesicle proteins in response to stimulation (relates to Fig 2).** In this set of experiments, we determined the fraction of synaptic vesicle molecules tagged with antibody (as described in Table 1) that are still able to exocytose in response to an external stimulus designed to release the entire recycling pool (600 action potentials). Right after tagging, almost all vesicle molecules are releasable. The releasable fraction steadily declines until almost none of the remaining tagged molecules are releasable 7-10 days later.

| Figure | Fig 2b-e |
| --- | --- |
| number of experiments | Synaptotagmin 1: 4 (day 0), 3 (day 1), 4 (day 2), 4 (day 4), 2 (day 7), 2 (day 10) independent experiments, >10 neurons imaged per experiment  VGAT: 4 (day 0), 4 (day 1), 2 (day 2), 4 (day 4), 4 (day 7), 3 (day 10) independent experiments, >10 neurons imaged per experiment |
| antibodies used | Synaptotagmin 1: Synaptic Systems, 105 311CpH, clone 604.2, lumenal domain, conjugated to CypHer5E  VGAT: Synaptic Systems, 131 103CpH, lumenal domain, conjugated to CypHer5E |
| antibody live tagging | Synaptotagmin 1 or VGAT antibody was applied (1:120 from 1 mg/ml stock), to live primary hippocampal neurons, in their own culture medium, for 1 h at 37°C in a cell culture incubator. The antibody was then washed off with ice-cold Tyrode’s solution (3-times on/off), and the cultures were maintained in their own culture medium until processing for their respective time point. |
| description of time course | Live tagging of releasing synaptic vesicles was performed (as described in the previous table row), right before processing for the initial time point (day 0). Separate cultures for each time point (day 0, day 1, day 2, day 4, day 7, day 10) were pulsed in parallel and maintained in the incubator until processing. On the respective time points, cultures were imaged live during stimulation with 600 action potentials (see next table row) before fixation and imaging in low- and high-pH buffers for normalization (see two table rows below). |
| stimulation paradigm | during live antibody tagging and time course : no external stimulation, only intrinsic network activity of primary hippocampal cultures  during experiment, to test releasability of vesicles: 600 action potentials delivered at 20 Hz in electrical field stimulation (in Tyrode’s solution with bafilomycin, to prevent re-acidification during imaging) |
| fixation and processing | Synaptotagmin 1 and VGAT: fixation in methanol (20 min, -20°C) after live imaging (with stimulation), no additional immunostaining. To determine the entire amount of antibodies present in the preparation, an application of pH 5.5 TES buffered salt solution was performed to activate CypHer5E. To determine the background fluorescence, not due to CypHer5E antibodies, an application of pH 7.4 PBS, to quench all CypHer5E molecules, was performed. |
| imaging setup | Synaptotagmin 1: Olympus, 60x apochromat oil immersion objective  VGAT: Nikon Ti-E, 60x apochromat oil immersion objective; heating chamber to maintain neurons at 37°C during imaging |

**Table 3: Co-localization of actively recycling synaptic vesicles and aged synaptic vesicles with metabolic markers for recently produced proteins (relates to Fig 3 and Fig 4).** In this set of experiments, we tagged recycling synaptic vesicles (as described in Table 1), and determined the co-localization of the fluorophore-conjugated antibody with markers for recently produced proteins. We used two approaches to reveal recently produced proteins: FUNCAT, in which the unnatural amino acid AHA is incorporated into proteins during biogenesis, and can be detected after fluorophore-conjugation, and COIN, in which heavy ^15^N leucine is incorporated into proteins during biogenesis, and can be detected with a nanoSIMS mass spectrometry imaging device. The analysis shows that actively recycling synaptic vesicles have a significantly stronger co-localization with recently produced proteins.

| Figure | Fig 3c-e (FUNCAT), Fig 4 (COIN) |
| --- | --- |
| number of experiments | FUNCAT (Fig 3c-e): 3 (actively recycling vesicles, day 0), 3 (inactive vesicles, day 4) independent experiments, >10 neurons sampled per experiment  COIN (Fig 4): 57 synapses from 3 independent experiments (actively recycling vesicles, day 0), 47 synapses from 2 independent experiments (inactive vesicles, day 4) |
| statistics | FUNCAT (Fig 3e): the unpaired t-test determined a significant difference, with p = 0.0037, t(4) = 6.09.  COIN (Fig 4b): the unpaired t-tests determined that the difference between releasable and inactive vesicles was significant, with p = 0.0001, t(102) = 5.5378. Unpaired t-tests further determined that the difference between releasable vesicles and the axon was significant, with p = 0.0004, t(116) = 3.6156, and that the difference between inactive vesicles and the axon was significant, with p = 0.0001, t(96) = 4.0691 (see figure legend for details). |
| antibodies used | live antibody tagging, FUNCAT and COIN: lumenal domain of Synaptotagmin 1, Synaptic Systems, 105 311AT, clone 604.2, against, conjugated to Atto647N  co-immunostaining after fixation, COIN: Synaptophysin, Synaptic Systems, 101 004 |
| antibody live tagging | Synaptotagmin 1 or VGAT antibody was applied (1:120 from 1 mg/ml stock), to live primary hippocampal neurons, in their own culture medium, for 1 h at 37°C in a cell culture incubator. The antibody was then washed off with ice-cold Tyrode’s solution (3-times on/off), and the cultures were maintained in their own culture medium until processing for their respective time point. |
| metabolic labelling | FUNCAT: AHA was fed to the cultured neurons (in culture medium free of methionine, which is the amino acid AHA competes with for integration into proteins during biogenesis) for 9 h prior to processing the samples for the respective time points (see below).  COIN: ^15^N leucine was fed to the cultured neurons (in 3-fold molar excess over ^14^N leucine in the culture medium) for 1-3 days prior to processing the samples for the respective time points (see below). The extended feeding time compared to AHA in FUNCAT was necessary to reliably obtain a signal in nanoSIMS imaging. |
| description of time course | For actively recycling vesicles (d0 time point, directly after antibody tagging): AHA was fed to the cultured neurons for 9 h (see table row above), ^15^N leucine was fed to the cultured neurons for 1-3 days (see table row above); this metabolic labelling was immediately followed by live antibody tagging of actively recycling synaptic vesicles (see two table rows above).  For inactive vesicles (day 4 time point after antibody tagging): live antibody tagging of actively recycling synaptic vesicles in cultured neurons was performed (see two table rows above), and the neurons were maintained in culture for 4 days until processing. During the last 9 hours of this 4 day interval, AHA was fed to the cultured neurons. Alternatively, during the last 24-72 hours of this 4 day interval, ^15^N leucine was fed to the cultured neurons. |
| stimulation paradigm | no external stimulation, only intrinsic network activity of primary hippocampal cultures during live antibody tagging and time course |
| fixation and processing | FUNCAT: 4% PFA (15 min 4°C, 30 min on room temperature), standard immunostaining for Synaptophysin to detect synapses, melamine embedding and thin-sectioning at 20 nm per slice  COIN: 4% PFA (15 min 4°C, 30 min on room temperature), standard immunostaining for Synaptophysin and PSD95 to detect synapses, LR-White embedding and thin-sectioning at 200 nm per slice; melamine embedding is unsuitable for nanoSIMS imaging, as the amines in the resin mask the mass spectrometry signal from the sample in the nitrogen channel |
| imaging setup | FUNCAT: Leica TCS SP5 STED (two-color STED mode), 100x apochromat oil immersion objective  COIN, fluorescence imaging: Nikon Ti-E, 100x apochromat oil immersion objective, additional 1.5x magnification lens  COIN, ^15^N leucine mass spectrometry imaging: Cameca nanoSIMS 50L |

**Table 4: Blocking Synaptotagmin 1 epitopes on actively recycling synaptic vesicles to observe the influx of unused epitopes from biogenesis or from the reserve pool (relates to Fig 5b,c).** In this set of experiments, we used monoclonal antibodies not conjugated to any fluorophores to block access to actively recycling epitopes for clonally identical fluorophore-conjugated antibodies applied afterwards. This allowed us to monitor the influx of new, recently unused (and thus unblocked) epitopes into the recycling pool of synaptic vesicles. We applied drugs that prevent the production or transport of new synaptic vesicles to dissect whether the new, not recently used (and thus unblocked) epitopes enter the recycling pool from biogenesis or from the reserve pool. We found that the reserve pool alone is unable to provide synaptic vesicles for release under physiological stimulation conditions, and that they come from biogenesis.

| Figure | Fig 5b,c |
| --- | --- |
| number of experiments | 4 independent experiments for time point 0 h of the untreated condition, 3 independent experiments for all other data points, >10 neurons sampled per experiment |
| statistics | Fig 5c: one-way ANOVA indicated that significant differences were present in the data, with p = 0.2205, F(2, 8) = 1.97. Significant differences were found with the post-hoc Bonferroni procedure between the conditions “untreated” and “anisomycin” (p = 0.0070) as well as between the conditions “untreated” and “colchicine” (p = 0.0022). All other comparisons were not significant. |
| antibodies used | for blocking epitopes of recently exocytosed Synaptotagmin 1: Synaptic Systems, 105 311, clone 604.2, lumenal domain, unconjugated  for detecting new, unblocked Synaptotagmin 1: Synaptic Systems, 105 311AT, clone 604.2, lumenal domain, conjugated to Atto647N |
| antibody live tagging | For blocking: unconjugated Synaptotagmin 1 antibody was applied (1:120 from 1 mg/ml stock), to live primary hippocampal neurons, in their own culture medium, for 2 h at 37°C in the cell culture incubator. The antibody was then washed off with ice-cold Tyrode’s solution (3-times on/off), and the cultures were maintained in their own culture medium until processing for their respective time point.  For detection: conjugated Synaptotagmin 1 antibody was applied (1:120 from 1 mg/ml stock), to live primary hippocampal neurons, in their own culture medium, for 30 min at 37°C in the cell culture incubator. The antibody was then washed off with ice-cold Tyrode’s solution (3-times on/off), and the cultures were maintained in their own culture medium until processing for their respective time point. |
| drug application | anisomycin (40 µM) to inhibit protein biogenesis, or colchicine (10 µM) to disrupt vesicle transport along the microtubule network |
| description of time course | The unconjugated antibody was applied for blocking Synaptotagmin 1 epitopes on actively recycling synaptic vesicles to all samples for all time points in parallel, 2 h before the first 30 min pulse of fluorophore-conjugated antibody. The fluorophore-conjugated antibody was then applied at the respective time points, and the samples were fixed and processed (see two table rows below). |
| stimulation paradigm | no external stimulation, only intrinsic network activity of primary hippocampal cultures during live antibody blocking and tagging for detection during the time course. |
| fixation and processing | 4% PFA (15 min 4°C, 30 min on room temperature), standard immunostaining for Synaptophysin to detect synapses, embedded in Mowiol |
| imaging setup | Leica TCS SP5 (confocal mode), 63x apochromat oil immersion objective |

**Table 5: Controls for synaptic activity during drug treatments described in Table 4 (relates to Fig 5d-f).** As the drug treatments described above (Table 4, Fig 5a-c) might by themselves alter synaptic activity, irrespective of any effect arising solely from the cut-off of synapses from newly produced vesicles, we performed several controls addressing the following physiological parameters of untreated and drugged cultures: fraction of actively recycling synaptic vesicles in response to stimulation, fraction of actively recycling synaptic vesicles during intrinsic network activity, and size of the total pool of synaptic vesicles per synapse. We found that none of these parameters were significantly altered by the drug treatments we employed here.

| Figure | Fig 5d (fraction of actively recycling synaptic vesicles in response to stimulation), Fig 5e (fraction of actively recycling synaptic vesicles during intrinsic network activity), Fig 5f (size of the total pool of synaptic vesicles per synapse). |
| --- | --- |
| number of experiments | number of independent experiments per condition: 3 (all data points) |
| statistics | Fig 5d: one-way ANOVA determined that no significant differences were present in the data, with p = 0.2205, F(2, 8) = 1.97.  Fig 5e: one-way ANOVA determined that no significant differences were present in the data, with p = 0.8835, F(2, 8) = 0.13.  Fig 5f: one-way ANOVA determined that no significant differences were present in the data, with p = 0.5519, F(2, 8) = 0.66. |
| antibodies used | Synaptotagmin 1: Synaptic Systems, 105 311AT, clone 604.2, lumenal domain, conjugated to Atto647N |
| antibody live tagging | Synaptotagmin 1 antibody was applied (1:120 from 1 mg/ml stock), to live primary hippocampal neurons, in their own culture medium, for 1 h at 37°C in a cell culture incubator. The antibody was then washed off with ice-cold Tyrode’s solution (3-times on/off), and the cultures were maintained in their own culture medium until processing for their respective time point. Alternatively, for live antibody tagging during stimulation (Fig 5d), the same antibody was applied during stimulation in Tyrode’s solution at room temperature, followed by a resting period of 5 min to allow synaptic vesicle recycling, followed by fixation. |
| drug application | anisomycin (40 µM) to inhibit protein biogenesis, or colchicine (10 µM) to disrupt vesicle transport along the microtubule network |
| description of time course | Neurons were treated with drugs (as described in Table 4) or left untreated for 24 h (the longest time point in time courses of experiments described in Table 4, Fig 5b,c). Then antibodies for live tagging were applied either during intrinsic network activity (Fig 5e) or during electrical field stimulation (Fig 5d). The amount of synaptic vesicles (Fig 5f) was determined from the samples used in Fig 5d, using a post-fixation immunostaining for Synaptophysin (>95% localization to synaptic vesicles). |
| stimulation paradigm | for 5e: no external stimulation, only intrinsic network activity of primary hippocampal cultures during live antibody tagging  for 5d: 600 action potentials delivered at 20 Hz in electrical field stimulation during antibody live tagging |
| fixation and processing | 4% PFA (15 min 4°C, 30 min on room temperature), standard immunostaining for Synaptophysin to detect synapses, embedded in Mowiol |
| imaging setup | Leica TCS SP5 (confocal mode), 63x apochromat oil immersion objective |

**Table 6: Increased synaptic activity results in faster inactivation of actively recycling synaptic vesicles (relates to Fig 6).** In this set of experiments, we pharmacologically increased the intrinsic network activity of primary hippocampal cultures to observe whether this would result in an accelerated inactivation of synaptic vesicles. We found that synaptic vesicles are inactivated faster when network activity is increased, suggesting that inactivation depends on the number of times vesicles have been used, rather than the amount of time that has passed since their biogenesis.

| Figure | Fig 6 |
| --- | --- |
| number of experiments | >30 neurons from 3 independent experiments for all conditions (untreated, bicuculline, Ca^2+^ 8 mM) |
| statistics | Fig 6c: one-way ANOVA indicated that significant differences were present in the data, with p = 3.68e-12, F(2, 86) = 36.61. Significant differences were found with the post-hoc Bonferroni procedure between the conditions “untreated” and “bicuculline” (p < 0.0001) as well as between the conditions “untreated” and “Ca^2+^ 8mM” (p < 0.0001). All other comparisons were not significant. |
| antibodies used | Synaptotagmin 1 live tagging: Synaptic Systems, 105 311, clone 604.2, against lumenal domain of Synaptotagmin 1, unconjugated  live detection of Synaptotagmin 1 primary antibody via a secondary antibody: Dianova, 115-175-146, anti-mouse, conjugated to Cy5, dialyzed into Tyrode’s solution for application in live cell immunolabeling |
| antibody live tagging | Unconjugated Synaptotagmin 1 antibody was applied (1:120 from 1 mg/ml stock), to live primary hippocampal neurons, in their own culture medium, for 1 h at 37°C in the cell culture incubator. The antibody was then washed off with ice-cold Tyrode’s solution (3-times on/off), and the cultures were maintained in their own culture medium until processing.  Secondary antibody conjugated to Cy5, against the Synaptotagmin 1 antibody, was applied live, 12 h after tagging with the Synaptotagmin 1 antibody, in the neurons’ own culture medium, for 1 h at 37°C in the cell culture incubator, to detect those epitopes still in the recycling pool. Secondary antibody conjugated to Cy3, against the Synaptotagmin 1 antibody, was then applied after fixation and permeabilization to detect inactive epitopes no longer in the recycling pool. |
| drug application | 20 µM bicuculline or 8 mM Ca^2+^ were applied to the neuron cultures directly after antibody tagging and kept there until processing of the samples 12 h later. |
| description of time course | At time point 0 h, recycling synaptic vesicles were tagged with the unconjugated antibody against Synaptotagmin 1, followed by detection of this primary antibody in the recycling population via live secondary labelling 12 h later, under the three conditions described above (untreated, bicuculline, Ca^2+^). Following fixation and permeabilization, the internalized population of tagged vesicles that was no longer participating in release was revealed using a differently conjugated secondary antibody. |
| stimulation paradigm | no external stimulation, only intrinsic network activity (manipulated through the application of bicuculline or Ca^2+^, which both increased the activity by ~50%) of primary hippocampal cultures during live antibody blocking and tagging for detection during the time course |
| fixation and processing | 4% PFA (15 min 4°C, 30 min on room temperature), standard immunostaining for Synaptophysin to detect synapses, embedded in Mowiol |
| imaging setup | Leica TCS SP5 (confocal mode), 63x apochromat oil immersion objective |

**Table 7: Changes in the protein composition of synaptic vesicles as they age (relates to Fig 8 and Appendix Fig S17a).** In this set of experiments, we used two-color STED microscopy to investigate the association of young (0 days after live antibody tagging) and old (4 days after live antibody tagging) synaptic vesicles with various synaptic proteins. The goal of these experiments was to determine whether the molecular composition of synaptic vesicles is altered during their life cycle. We did not find any significant changes, apart from a 2-fold increase of SNAP25 association with old vesicles compared to young vesicles.

| Figure | Fig 8, Appendix Fig S17a |
| --- | --- |
| number of experiments | number of independent experiments per protein of interest (d0, [# of vesicles analyzed], d4 [# of vesicles analyzed]), >10 neurons sampled per experiment: SNAP25 (4 [36599], 3 [35419]), Syntaxin 1 (3 [17186], 3 [13231]), VGlut 1/2 (2 [20109], 3 [26268]), vATPase (3 [14506], 4 [38032]), VAMP2 (4 [29213], 4 [31892]), Synaptotagmin 1 (3 [23387], 3 [17523]), Syntaxin 16 (4 [32862], 4 [32199]), VAMP4 (3 [22538], 3 [26999]), Synapsin I/II (3 [15735], 3 [17656]). |
| statistics | Fig 8c: the unpaired t-test determined that the difference between day 0 and day 4 was significant for SNAP25, with p = 0.0124, t(5) = 3.8200. Unpaired t-tests found no significant differences between day 0 and day 4 for all other proteins of interest: for Syntaxin 1, p = 0.8850, t(4) = 0.1541; for VGlut 1/2, p = 0.1986, t(3) = 1.6447; for vATPase, p = 0.7340, t(5) = 0.3594; for VAMP2, p = 0.8837, t(6) = 0.1527; for Synaptotagmin 1, p = 0.1604, t(4) = 1.7208; for Syntaxin 16, p = 0.7406, t(6) = 0.3468; for VAMP4, p = 0.9863, t(4) = 0.0183; for Synapsin I/II, p = 0.6638, t(4) = 0.4685. |
| antibodies used | Synaptotagmin 1 live tagging: Synaptic Systems, 105 311AT, clone 604.2, lumenal domain, conjugated to Atto647N  immunostainings for proteins of interest, after fixation: SNAP25 (Synaptic Systems, 111 002), Syntaxin 1 (Synaptic Systems, 110 302), VGlut 1/2 (Synaptic Systems, 135 503), vATPase (Synaptic Systems, 109 002), VAMP2 (Synaptic Systems, 104 202), Synaptotagmin 1 (Synaptic Systems, 105 102), Syntaxin 16 (Synaptic Systems, 110 162), VAMP4 (Synaptic Systems, 136 002), Synapsin I/II (Synaptic Systems, 106 002) |
| antibody live tagging | Synaptotagmin 1 antibody was applied (1:120 from 1 mg/ml stock), to live primary hippocampal neurons, in their own culture medium, for 1 h at 37°C in a cell culture incubator. The antibody was then washed off with ice-cold Tyrode’s solution (3-times on/off), and the cultures were maintained in their own culture medium until processing for their respective time point. |
| description of time course | Synaptic vesicles in primary hippocampal cultures were tagged (as described in the previous table row). Directly after tagging, half of the cultures were processed immediately (day 0 time point, young synaptic vesicles); the other half were kept in culture for processing on day 4 after tagging (day 4 time point, aged synaptic vesicles); see two table rows below for details on processing. |
| stimulation paradigm | no external stimulation, only intrinsic network activity of primary hippocampal cultures during live antibody tagging and time course |
| fixation and processing | 4% PFA (15 min 4°C, 30 min on room temperature), standard immunostaining for proteins of interest (see above), melamine embedding and thin-sectioning at 20 nm per slice |
| imaging setup | Leica TCS SP5 STED (two-color STED mode), 100x apochromat oil immersion objective |

**Table 8: Expression of sypHy, sypHy-SNAP25, or sypHy-Syntaxin 1 to determine whether increased amounts of SNAP25 on a synaptic vesicle lead to its inactivation (relates to Fig 9, Appendix Fig S18, and Appendix Fig S26).** In this set of experiments, we expressed the lumenal pH-sensor sypHy on synaptic vesicles, as well as a sypHy construct coupled to SNAP25, and compared the response of both constructs to stimulation. In case of sypHy-SNAP25, release was severely depressed.

| Figure | Fig 9, Appendix Fig S18, Appendix Fig S26 |
| --- | --- |
| number of experiments | 9 independent experiments for sypHy, 9 independent experiments for sypHy-SNAP25, 12 independent experiments for sypHy-Syntaxin 1 |
| statistics | Fig 9e: one-way ANOVA indicated that significant differences were present in the data, with p = 0.0035, F(2, 31) = 6.90. Significant differences were found with the post-hoc Bonferroni procedure between the conditions “sypHy” and “sypHy-SNAP25” (p = 0.0160) as well as between the conditions “sypHy” and “sypHy-Syntaxin 1” (p = 0.0050). All other comparisons were not significant.  Appendix Fig S18b: one-way ANOVA determined that no significant differences were present in the data, with p = 0.6816, F(2, 17) = 0.39.  Appendix Fig S26e: one-way ANOVA determined that no significant differences were present in the data, with p = 0.0705, F(2, 30) = 2.92. |
| constructs used | sypHy (Synaptophysin coupled to pH-sensitive GFP-variant in an internal lumenal loop)  sypHy-SNAP25 (Synaptophysin coupled to pH-sensitive GFP-variant in an internal lumenal loop and SNAP25, mutated to remove all palmitoylation sites, on the cytoplasmic C-terminus)  sypHy-Syntaxin 1 (Synaptophysin coupled to pH-sensitive GFP-variant in an internal lumenal loop, and Syntaxin 1, without the membrane-integration domain and the intra-vesicular domain, on the cytoplasmic C-terminus) |
| description of time course | Neurons were transfected and maintained in culture for 3-4 days, until expression was sufficient for imaging. The neurons were then subjected to stimulation during imaging (see below) to detect differences in release between the two constructs. |
| stimulation paradigm | 600 action potentials (Fig 9) or 60 action potentials (Appendix Fig S26) delivered at 20 Hz in electrical field stimulation. |
| fixation and processing | no fixation, application of a pulse of 100 mM NH_4_Cl to achieve maximum fluorescence of all sypHy proteins in the neurons for normalization of the data acquired during the stimulation (Fig 9 and Appendix Fig S26) |
| imaging setup | Nikon Ti-E, 60x apochromat oil immersion objective; heating chamber to maintain neurons at 37°C during imaging |

**Table 9: SNAP25 overexpression leads to inactivation of vesicle recycling, which is reversed by overexpression of CSPα (relates to Fig 10 and Appendix Figs S20-25).** In this set of experiments, we further tested our assumption that SNAP25 overexpression leads to the inactivation of synaptic vesicle recycling. We also tested our hypothesis that overexpression of CSPα can compensate for this inactivation. We tested the level of recycling (the size of the actively recycling pool) with a live antibody tagging assay. The results show that overexpression of SNAP25 leads to decreased release, overexpression of CSPα leads to increased release, and co-overexpression of both proteins also leads to increased release.

| Figure | Fig 10a-g, Appendix Figs S20-25 |
| --- | --- |
| number of experiments | number of independent experiments (independent experiments, neurons imaged in total): SNAP25 (4, 10), Syntaxin 1 (7, 13), CSPα_WT_ (4, 14), CSPα_mut_ (4, 11), SNAP25 + CSPα_WT_ (8, 20), SNAP25 + CSPα_mut_ (6, 20) |
| statistics | Fig 10b: the paired t-test determined that the difference between control and over-expression was significant, with p = 0.0017, t(3) = 10.8862.  Fig 10c: the paired t-test determined that the difference between control and over-expression was significant, with p = 0.0003, t(6) = 7.5661.  Fig 10d: the paired t-test determined that the difference between control and over-expression was significant, with p = 0.0034, t(3) = 8.5006.  Fig 10e: the paired t-test determined that the difference between control and over-expression was not significant, with p = 0.6004, t(3) = 0.5837.  Fig 10f: the paired t-test determined that the difference between control and over-expression was significant, with p = 0.0173, t(7) = 3.0992.  Fig 10g: the paired t-test determined that the difference between control and over-expression was significant, with p = 0.0165, t(5) = 3.5410. |
| antibodies used | Synaptotagmin 1 live tagging: Synaptic Systems, 105 311AT, clone 604.2, lumenal domain, conjugated to Atto647N  co-immunostaining after fixation: Synaptic Systems, 101 004, Synaptophysin |
| constructs used | YFP-SNAP25 (YFP for detection of expression), CSPα_WT_ (wild-type CSPα, mCherry IRES for detection of expression), CSPα_mut_ (CSPα mutated to not target to vesicle membrane, mCherry IRES for detection of expression), Syntaxin 1-YFP (YFP for detection of expression) |
| antibody live  tagging | Synaptotagmin 1 antibody was applied (1:120 from 1 mg/ml stock), to live primary hippocampal neurons, in their own culture medium, for 1 h at 37°C in a cell culture incubator. The antibody was then washed off with ice-cold Tyrode’s solution (3-times on/off), and the cultures were maintained in their own culture medium until processing for their respective time point. |
| description of time course | Neurons were transfected with the respective constructs and maintained in culture for 3-4 days, until expression was sufficient for imaging. The actively recycling synaptic vesicles were then tagged live with Synaptotagmin 1 antibody for 1 h, to determine the actively recycling population under intrinsic network activity conditions. This served as a measure for the overall activity state of synaptic vesicles in neurons expressing the respective constructs, and thus for synaptic vesicle releasability during altered molecular composition. The cells were immunostained for Synaptophysin after fixation, to detect the synaptic boutons, and the intensity of the Synaptotagmin 1 labeling was determined in Fig 10. The intensity and geometry of the Synaptophysin immunostaining was determined in Appendix Figs S20-25. |
| stimulation paradigm | no external stimulation, only intrinsic network activity of primary hippocampal cultures during live antibody tagging |
| fixation and processing | 4% PFA (15 min 4°C, 30 min on room temperature), immunostaining for Synaptophysin, embedded in Mowiol |
| imaging setup | Leica TCS SP5 (confocal mode), 63x apochromat oil immersion objective for Fig 10a,b,d-f, Appendix Figs S20, S21, S23-25  Nikon Ti-E, 100x apochromat oil immersion objective with additional 1.5x lense (for a total 150x magnification) for Fig 10c, Appendix Fig S22 |

**Table 10: Synaptic vesicles do not disintegrate upon fusion to the cell membrane, but the molecules are maintained as meta-stable assemblies (relates to Appendix Fig S1).** We allowed synaptic vesicle release to take place, and then tagged the two synaptic vesicle proteins Synaptotagmin 1 and Synaptophysin on the cell surface. We found that both markers co-localized to a large extent when investigated with two-color STED microscopy, and that only ~3% of Synaptotagmin 1 epitopes appeared to be lost from the synaptic vesicle clusters.

| Figure | Appendix Fig S1 |
| --- | --- |
| number of experiments | 3 independent experiments (comprising 1219 synaptic vesicle patches and 136 Synaptotagmin 1 spots outside the patches). |
| antibodies used | for blocking epitopes of recently exocytosed Synaptotagmin 1: Synaptic Systems, 105 311, clone 604.2, lumenal domain, unconjugated  for detecting new, unblocked Synaptotagmin 1: Synaptic Systems, 105 311AT, clone 604.2, lumenal domain, conjugated to Atto647N  for detecting Synaptophysin on the cell surface: G96 serum, a kind gift from Reinhard Jahn, Max Planck Institute for Biophysical Chemistry, Göttingen |
| antibody live  tagging | Synaptotagmin 1 or Synaptophysin antibodies were applied to live primary hippocampal neurons, as explained in the description of the time course below. |
| description of time course | Surface epitopes of Synaptotagmin 1 in primary hippocampal neuron cultures were blocked with the unconjugated Synaptotagmin 1 antibody for 10 min in Ca^2+^-free buffer (to inhibit synaptic vesicle recycling during blocking). After washing of the antibodies, the neurons were allowed to release synaptic vesicles for 6 min in the presence of the Atto647N-conjugated Synaptotagmin 1 antibody, to tag epitopes of those synaptic vesicles that exocytosed during that time. After the 6 min were over, the neurons were fixed, and an antibody against Synaptophysin was applied to reveal this molecule. The Synaptophysin antibody was revealed by secondary staining, and the samples were imaged in two-color STED microscopy. |
| stimulation paradigm | during blocking Synaptotagmin 1 surface epitopes with the unconjugated Synaptotagmin 1 antibody: Ca^2+^-free buffer, to inhibit synaptic vesicle recycling  during live antibody tagging with the Atto647N-conjugated Synaptotagmin 1 antibody: 15 mM KCl at 4°C, to facilitate exocytosis but prevent endocytosis. |
| fixation and processing | 4% PFA (15 min on ice, 30 min on room temperature), standard immunostaining for Synaptophysin to detect synapses, but without permeabilization, to only detect surface epitopes, embedded in Mowiol |
| imaging setup | two-color STED setup, see figure legend and Material and Methods |

**Table 11: The Synaptotagmin 1 antibody is taken up in a specific fashion (relates to Appendix Fig S2).** In this set of experiments we determined whether Synaptotagmin 1 antibodies conjugated to Atto647N are taken up more efficiently than non-specific secondary antibodies against mouse IgG conjugated to Atto647N, which do not recognize lumenal epitopes in neurons. The antibodies were applied to live primary hippocampal neuron cultures.

| Figure | Appendix Fig S2 |
| --- | --- |
| number of experiments | 3 independent experiments per condition, >10 neurons imaged per experiment |
| antibodies used | Synaptotagmin 1: Synaptic Systems, 105 311AT, clone 604.2, lumenal domain, conjugated to Atto647N  anti-mouse IgG: Dianova, 115-005-146, goat polyclonal, conjugated to Atto647N |
| antibody live tagging | Synaptotagmin 1 or anti-mouse IgG antibody was applied (1:120 from 1 mg/ml stock), to live primary hippocampal neurons, in their own culture medium, for 1 h at 37°C in a cell culture incubator. The antibody was then washed off with ice-cold Tyrode’s solution (3-times on/off), and the cultures were fixed and processed. |
| stimulation paradigm | No external stimulation, only intrinsic network activity of primary hippocampal cultures during live antibody tagging and time course |
| fixation and processing | 4% PFA (15 min on ice, 30 min on room temperature), standard immunostaining for Synaptophysin to detect synapses, embedded in Mowiol |
| imaging setup | Nikon Ti-E, 60x apochromat oil immersion objective |

**Table 12: Synaptotagmin 1 antibodies applied during live tagging do not lose contact to their epitopes for at least 10 days in culture (relates to Appendix Fig S3).** In this set of experiments, we strove to confirm that Synaptotagmin 1 antibodies applied during live tagging do not come off their epitopes during the time course we used in our other experiments. We fixed neurons, applied the antibody, and left the neurons for up to 10 days in the incubator at 37°C, in a pH 5.5 buffer to simulate the synaptic vesicle lumen, with a 100x molar excess of antigenic peptide. There was no detectable loss of antibody.

| Figure | Appendix Fig S3 |
| --- | --- |
| number of experiments | 5 (day 0), 3 (day 1), 3 (day 4), 3 (day 10) independent experiments, >10 neurons imaged per experiment |
| statistics | Appendix Fig S3b: one-way ANOVA determined that no significant differences were present in the data, with p = 0.9945, F(3, 13) = 0.02. |
| antibodies used | Synaptotagmin 1: Synaptic Systems, 105 311AT, clone 604.2, lumenal domain, conjugated to Atto647N |
| description of time course | After fixation and permeabilization (as described below), the Synaptotagmin 1 antibody was applied to the cultures. The fixed and labelled cultures were then maintained until their respective time point (day 0 directly after immunostaining, day 1, day 4, day 10) of processing at 37°C in the cell culture incubator, in a pH 5.5 TES-buffered salt solution (to simulate the intravesicular environment), with a 100x molar excess of antigenic peptide. |
| stimulation paradigm | no stimulation, all stainings performed post-fixation |
| fixation and processing | 4% PFA (15 min 4°C, 30 min on room temperature), 20 min 100 mM NH_4_Cl to quench residual PFA activity, time course as described two table rows above; post-fixation with 4% PFA (15 min 4°C, 30 min on room temperature), standard immunostaining for Synaptophysin to detect synapses, embedded in Mowiol |
| imaging setup | Leica TCS SP5 (confocal mode), 63x apochromat oil immersion objective |

**Table 13: Releasability of newly tagged synaptic vesicle proteins in response to stimulation (refers to Appendix Fig S4).** In this set of experiments, we determined the fraction of synaptic vesicle molecules tagged with antibody (as described in Table 1) that are still to exocytose in response to an external stimulus designed to release the entire recycling pool (1200 action potentials at 20 Hz) immediately after tagging.

| Figure | Appendix Fig S4 |
| --- | --- |
| number of experiments | 3 independent experiments, >10 neurons imaged per experiment |
| statistics | Appendix Fig S4b: the paired t-test determined that the release upon stimulation is highly significant, with p < 0.0001, t(2) = 82.1314. |
| antibodies used | Synaptotagmin 1: Synaptic Systems, 105 311CpH, clone 604.2, lumenal domain, conjugated to CypHer5E |
| antibody live tagging | Synaptotagmin 1 antibody was applied (1:120 from 1 mg/ml stock), to live primary hippocampal neurons, in their own culture medium, for 1 h at 37°C in a cell culture incubator. The antibody was then washed off with ice-cold Tyrode’s solution (3-times on/off), and the cultures were maintained in their own culture medium until processing for their respective time point. |
| description of time course | Live tagging of releasing synaptic vesicles was performed (as described in the previous table row), right before processing for the initial time point (day 0). The cultures were then imaged live during stimulation with 1200 action potentials before fixation and imaging in low- and high-pH buffers for normalization (see two table rows below). |
| stimulation paradigm | during live antibody tagging and time course: no external stimulation, only intrinsic network activity of primary hippocampal cultures  during experiment, to test releasability of vesicles: 1200 action potentials delivered at 20 Hz in electrical field stimulation (in Tyrode’s solution with bafilomycin, to prevent re-acidification during imaging) |
| fixation and processing | Fixation was done with methanol (20 min, -20°C) after live imaging (with stimulation) to preserve the pH-sensitivity and fluorescence of CypHer5E, no additional immunostaining. To determine the entire amount of antibodies present in the preparation, an application of pH 5.5 TES buffered salt solution was performed to activate CypHer5E. To determine the background fluorescence, not due to CypHer5E antibodies, an application of pH 7.4 PBS, to quench all CypHer5E molecules, was performed. |
| imaging setup | Nikon Ti-E, 60x apochromat oil immersion objective; heating chamber to maintain neurons at 37°C during imaging |

**Table 14: Available epitopes in the recycling population of synaptic vesicles are saturated after 30-60 min of incubation with lumenal domain Synaptotagmin 1 antibodies during intrinsic network activity (relates to Appendix Fig S5).** To determine which incubation time is necessary to tag the entire releasable and recycling population of synaptic vesicles in our primary hippocampal cultures, we incubated them with the lumenal domain Synaptotagmin 1 antibody for varying times and determined that saturation is achieved after 30-60 min. This was necessary to establish the experimental parameters for all other experiments, which rely on complete tagging of the releasable and recycling population of synaptic vesicles. Alternatively, the 60-minute incubated cultures were stimulated at 20 Hz for 30 seconds.

| Figure | Appendix Fig S5 |
| --- | --- |
| number of experiments | 3 independent experiments per time point, >10 neurons imaged per data point |
| antibodies used | Synaptotagmin 1: Synaptic Systems, 105 311AT, clone 604.2, lumenal domain, conjugated to Atto647N |
| antibody live  tagging | Synaptotagmin 1 antibody was applied (1:120 from 1 mg/ml stock), to live primary hippocampal neurons, in their own culture medium, for 1 min, 10 min, 30 min, or 6 0 min at 37°C in a cell culture incubator. The antibody was then washed off with Tyrode’s solution (3-times on/off), and the cultures were maintained in their own culture medium until processing for their respective time point. |
| description of conditions | Live-tagging with the Synaptotagmin 1 was allowed to proceed for 1 min, 10 min, 30 min, or 60 min to determine after what time saturation of all releasable epitopes would be achieved during intrinsic network activity. |
| stimulation paradigm | a-b: no external stimulation, only intrinsic network activity of primary hippocampal cultures during live antibody tagging and time course  c-d: 20 Hz, 30 seconds electrical stimulation |
| fixation and processing | 4% PFA (15 min 4°C, 30 min on room temperature), standard immunostaining for Synaptophysin to detect synapses and determine co-localization with the live tagging Synaptotagmin 1 antibody, embedded in Mowiol |
| imaging setup | a-b: Cytation 3 cell imaging, multi-mode reader (BioTek), equipped with a 20x objective  c-d: Nikon Ti-E epifluorescence microscope, equipped with a 100x objective |

**Table 15: The Synaptotagmin 1 antibody is taken up in synaptic vesicles (relates to Appendix Fig S6).** In this set of experiments we determined whether Synaptotagmin 1 antibodies are taken up in organelles that co-localize with the synaptic vesicle marker Synaptophysin, with the dense-core vesicle marker Chromogranin A, or with the endosomal markers Rab 5 and Rab 7. The Synaptotagmin 1 lumenal antibody was applied to live primary hippocampal neuron cultures. Following 1 h incubation at 37°C, the cultures were fixed, permeabilized and immunostained for the respective markers. Samples were analyzed by 2-color 3D STED microscopy.

| Figure | Appendix Fig S6 |
| --- | --- |
| number of experiments | 3 independent experiments, >10 neurons imaged per experiment, >1000 vesicles analyzed per experiment. |
| statistics | Appendix Fig S6b: one-way ANOVA indicated that significant differences were present in the data, with p < 0.0001, F(2, 12) = 332.86. Significant differences were found with the post-hoc Bonferroni procedure between the random data (negative control) and Synaptotagmin 1 (p < 0.0001) as well as between the random data (negative control) and Synaptophysin (p < 0.0001). All other comparisons were not significant.  Appendix Fig S6d: the unpaired t-test determined that there are no significant differences between the protein of interest and random data in association with Synaptotagmin 1 live tagging, with p = 0.9999, t(20) = 0.0001.  Appendix Fig S6e: the unpaired t-test determined that there are no significant differences between the protein of interest and random data in association with Synaptotagmin 1 live tagging, with p = 0.9999, t(20) < 0.0001.  Appendix Fig S6f: the unpaired t-test determined that there are no significant differences between the protein of interest and random data in association with Synaptotagmin 1 live tagging, with p = 0.9999, t(20) < 0.0001. |
| antibodies used | Synaptotagmin 1, for live-tagging: Synaptic Systems, 105 311AT, clone 604.2, lumenal domain, conjugated to Atto647N  Synaptophysin 1 (co-immunostaining): Synaptic Systems, 101 004, guinea pig polyclonal  Chromogranin A (co-immunostaining): Synaptic Systems, 259 003, rabbit polyclonal  Rab 5 (co-immunostaining): Cell Signaling, 3547, rabbit polyclonal  Rab 7 (co-immunostaining): Cell Signaling, 9367, rabbit polyclonal  secondary antibodies (co-immunostaining): goat anti-guinea pig IgG conjugated to Abberior STAR580 (Abberior, 2-0112-005-7) or goat anti-rabbit IgG conjugated to Abberior STAR580 (Abberior, 2-0012-005-8) |
| antibody live tagging | Synaptotagmin 1 antibody was applied (1:120 from 1 mg/ml stock), to live primary hippocampal neurons, in their own culture medium, for 1 h at 37°C in a cell culture incubator. The antibody was then washed off with ice-cold Tyrode’s solution (3-times on/off), and the cultures were fixed and processed. |
| stimulation paradigm | No external stimulation, only intrinsic network activity of primary hippocampal cultures during live antibody tagging and time course |
| fixation and processing | 4% PFA (15 min 4°C, 30 min on room temperature), 20 min 100 mM NH_4_^+^ to quench residual PFA activity, standard immunostaining for the proteins of interest, embedded in Mowiol |
| imaging setup | Abberior easy3D STED microscope (two-color STED mode), 100x apochromat oil immersion objective |

**Table 16: Investigation of the synaptic vesicle populations marked by antibody live tagging (relates to Appendix Fig S7).** In this set of experiments, we tested to which functional populations of synaptic vesicles the Synaptotagmin 1 antibody used for live tagging binds to. We found that it labels the surface pool (~27% of all epitopes), the spontaneously releasing pool of synaptic vesicles (~4% of all epitopes), and the recycling pool (~22% of all epitopes). The reserve pool (~47% of all epitopes) is not labelled by live cell tagging.

| Figure | Appendix Fig S7 |
| --- | --- |
| number of experiments | 3 independent experiments per condition, >10 neurons sampled per experiment |
| antibodies used | Synaptotagmin 1: Synaptic Systems, 105 311AT, clone 604.2, lumenal domain, conjugated to Atto647N |
| antibody live  tagging | Synaptotagmin 1 antibody was applied (1:120 from 1 mg/ml stock), to live primary hippocampal neurons, in their own culture medium, as described in the table row below for the different conditions. The antibody was then washed off with ice-cold Tyrode’s solution (3-times on/off), and the cultures were fixed and processed immediately as described below. |
| description of conditions | Live tagging of Synaptotagmin 1 (standard conditions, as used in most experiments here): 1 h of live tagging (as described in the table row above) at 37°C in a cell culture incubator.  Additional tagging of Synaptotagmin 1 after fixation and permeabilization: as above, but after fixation and permeabilization we performed an additional immunostaining for Synaptotagmin 1 with the same antibody to reveal all Synaptotagmin 1 epitopes (surface, recycling, and internalized). This was performed on the same coverslips used in the condition above, after cutting them in half after fixation (one half was treated as described in this condition, the other half was not subjected to an additional Synaptotagmin 1 staining).  Live tagging of Synaptotagmin 1 in TTX (0.5-1 µM): 1 h of live tagging (as described in the table row above) at 37°C in a cell culture incubator.  Live tagging of Synaptotagmin 1 on 4°C: 1 h of live tagging (as described in the table row above) at 4°C, with TTX (0.5-1 µM) added. |
| stimulation paradigm | no external stimulation, only intrinsic network activity of primary hippocampal cultures during live antibody tagging (or inhibition of activity through TTX or 4°C as described in the table row above). |
| fixation and processing | 4% PFA (15 min 4°C, 30 min on room temperature), standard immunostaining for Synaptophysin to detect synapses, embedded in Mowiol |
| imaging setup | Leica TCS SP5 (confocal mode), 63x apochromat oil immersion objective |

**Table 17: Synaptotagmin 1 antibodies applied during live tagging stay within synapses for up to 10 days in culture (relates to Appendix Fig S8).** In this set of experiments, we followed the association of the live antibody tag for Synaptotagmin 1 with synaptic vesicles throughout a time course of up to 10 days after live tagging. To do this, we live-tagged Synaptotagmin 1 with antibodies, as described in Table 1, and performed a co-immunostaining for Synaptophysin (as synaptic vesicle marker) after fixation of the cultures at each respective time point. We found that the association of the Synaptotagmin 1 antibody live tag did not significantly decrease, even after 10 days in culture.

| Figure | Appendix Fig S8 |
| --- | --- |
| number of experiments | 3 (day 0), 3 (day 1), 2 (day 2), 3 (day 4), 3 (day 7), 2 (day 10) independent experiments, >10 neurons imaged per experiment |
| statistics | Appendix Fig S8b: one-way ANOVA determined that no significant differences were present in the data, with p = 0.8582, F(5, 15) = 0.37. |
| antibodies used | Synaptotagmin 1: Synaptic Systems, 105 311AT, clone 604.2, lumenal domain, conjugated to Atto647N |
| antibody live  tagging | Synaptotagmin 1 antibody was applied (1:120 from 1 mg/ml stock), to live primary hippocampal neurons, in their own culture medium, for 1 h at 37°C in a cell culture incubator. The antibody was then washed off with ice-cold Tyrode’s solution (3-times on/off), and the cultures were maintained in their own culture medium until processing for their respective time point. |
| description of time course | Live tagging of releasing synaptic vesicles was performed (as described in the previous table row), right before processing for the initial time point (day 0). Separate cultures for each time point (day 0, day 1, day 2, day 4, day 7, day 10) were pulsed in parallel and maintained in the incubator until processing. |
| stimulation paradigm | no external stimulation, only intrinsic network activity of primary hippocampal cultures during live antibody tagging and time course. |
| fixation and processing | 4% PFA (15 min 4°C, 30 min on room temperature), standard immunostaining for Synaptophysin to detect synapses and determine co-localization with the live tagging Synaptotagmin 1 antibody, embedded in Mowiol |
| imaging setup | Leica TCS SP5 (confocal mode), 63x apochromat oil immersion objective |

**Table 18: Synaptotagmin 1 antibodies end up in lysosomes in the neuron soma after ~2-4 days (relates to Appendix Fig S9a,b).** In this set of experiments, we determined the sub-cellular localization of Synaptotagmin 1 antibodies in a time course after live tagging. We found that after 2 days in culture, the Synaptotagmin 1 antibody appeared in highly acidic (determined through CypHer5E fluorescence, which is brightly fluorescent in pH <5.5, Appendix Fig S9a; further determined via co-localization with Lysotracker, Appendix Fig S9b) compartments in the neuron soma, which were resistant to electrically stimulated release (did not move or decrease in brightness, as imaged with CypHer5E). We concluded that these are degradation organelles of the lysosomal lineage.

| Figure | Appendix Fig S9a,b |
| --- | --- |
| number of experiments | Appendix Fig S9a: 5 (day 0), 4 (day 1), 5 (day 2) independent experiments, >10 neurons imaged per experiment  Appendix Fig S9b: 3 independent experiments per time point, >10 neurons imaged per experiment (exemplary images shown) |
| antibodies used | Appendix Fig S9a: Synaptotagmin 1, lumenal domain, Synaptic Systems, 105 311CpH, lumenal domain, conjugated to CypHer5E  Appendix Fig S9b: Synaptotagmin 1, lumenal domain, Synaptic Systems, 105 311AT, conjugated to Atto647N |
| antibody live tagging | Synaptotagmin 1 antibody was applied (1:120 from 1 mg/ml stock), to live primary hippocampal neurons, in their own culture medium, for 1 h at 37°C in a cell culture incubator. The antibody was then washed off with ice-cold Tyrode’s solution (3-times on/off), and the cultures were maintained in their own culture medium until processing for their respective time point. |
| description of time course | Live tagging of releasing synaptic vesicles was performed (as described in the previous table row), right before processing for the initial time point (day 0). Separate cultures for each time point (day 0, day 1, day 2, day 4) were pulsed in parallel and maintained in the incubator until processing. On the respective time points, cultures were imaged live after stimulation with 600 action potentials (see next table row) to reveal non-releasable CypHer5E-coupled antibody (Appendix Fig S9a) or incubated with Lysotracker and immediately imaged live (Appendix Fig S9b). |
| stimulation paradigm | during live antibody tagging and time course: no external stimulation, only intrinsic network activity of primary hippocampal cultures  during experiment, to test releasability Synaptotagmin 1-positive acidic organelles in the neuron soma: 600 action potentials delivered at 20 Hz in electrical field stimulation (in Tyrode’s solution with bafilomycin, to prevent re-acidification during imaging) |
| fixation and processing | no fixation, only live imaging |
| imaging setup | Nikon Ti-E, 60x apochromat oil immersion objective; heating chamber to maintain neurons at 37°C during imaging |

**Table 19: Inhibition of lysosome activity delays loss of live tagged Synaptotagmin 1 (relates to Appendix Fig S9c).** In this set of experiments, we tested whether the live tagging Synaptotagmin 1 antibody is degraded via lysosomes. We performed live antibody tagging and inhibited lysosomal activity for 24 h, and compared the amount of fluorescence intensity in synapses to a control condition where lysosomal activity was not inhibited. We found that there was significantly more fluorescence signal remaining in neuros where lysosomal activity was inhibited.

| Figure | Appendix Fig S9c |
| --- | --- |
| number of experiments | 3 independent experiments per data point, >10 neurons imaged per experiment |
| statistics | The unpaired t-test determined that the difference between the untreated control condition and the condition treated with leupeptin was significant, with p = 0.0237, t(4) = 3.5561. |
| antibodies used | Synaptotagmin 1: Synaptic Systems, 105 311AT, lumenal domain, conjugated to Atto647N |
| antibody live tagging | Synaptotagmin 1 antibody was applied (1:120 from 1 mg/ml stock), to live primary hippocampal neurons, in their own culture medium, for 1 h at 37°C in a cell culture incubator. The antibody was then washed off with ice-cold Tyrode’s solution (3-times on/off), and the cultures were maintained in their own culture medium until processing for their respective time point. |
| drug application | leupeptin (100 µM) to inhibit lysosomal enzymes |
| description of time course | Live tagging of releasing synaptic vesicles was performed (as described above). The cultures were then maintained for 24 h in the incubator, either without treatment or with the leupeptin treatment described in the table row above. After 24 h, the cultures were processed (see two table rows below), imaged, and compared. |
| stimulation paradigm | no external stimulation, only intrinsic network activity of primary hippocampal cultures |
| fixation and processing | 4% PFA (15 min 4°C, 30 min on room temperature), standard immunostaining for Synaptophysin to detect synapses, embedded in Mowiol |
| imaging setup | Leica TCS SP5 (confocal mode), 63x apochromat oil immersion objective |

**Table 20: Neurons of different ages show similar levels of synaptic activity, and similar synapses (relates to Appendix Figs S10 and S11).** To determine whether the neurons change during the long experiments, we incubated cultures with Synaptotagmin 1 antibodies at different days *in vitro*, and we also immunostained them for Synaptophysin, to determine their synapse morphologies.

| Figure | Appendix Fig S10, Appendix Fig S11 |
| --- | --- |
| number of experiments | 3 independent experiments per time point, >10 neurons imaged per data point |
| statistics | Appendix Fig S10b: one-way ANOVA determined that no significant differences were present in the data, with p = 0.3046, F(5, 17) = 1.36.  Appendix Fig S10b-g: one-way ANOVAs determined that no significant differences were present in the data, with (b) p = 0.4743, F(5, 17) = 0.97; (c) p = 0.3046, F(5, 17) = 1.36; (d) p = 0.6040, F(5, 17) = 0.75; (e) p = 0.6899, F(5, 17) = 0.62; (f) p = 0.3462, F(5, 17) = 1.25; (g) p = 0.7542, F(5, 17) = 0.52. |
| antibodies used | Synaptotagmin 1 (live-tagging): Synaptic Systems, 105 311AT, clone 604.2, lumenal domain, conjugated to Atto647N  Synaptophysin (co-immunostaining): Synaptic Systems, 101 004, guinea pig polyclonal  secondary antibody (co-immunostaining): goat anti-guinea pig conjugated to Abberior STAR580 (Abberior, 2-0112-005-7) |
| antibody live  tagging | Synaptotagmin 1 antibody was applied (1:120 from 1 mg/ml stock), to live primary hippocampal neurons, in their own culture medium, for 60 min at 37°C in a cell culture incubator. The antibody was then washed off with Tyrode’s solution (3-times on/off), and the cultures were fixed and processed. |
| description of conditions | Live-tagging with the Synaptotagmin 1 was allowed to proceed for 1 hour, before fixation and immunostaining for Synaptophysin, in cultures of different ages (days in vitro). |
| stimulation paradigm | no external stimulation, only intrinsic network activity of primary hippocampal cultures during live antibody tagging and time course |
| fixation and processing | 4% PFA (15 min 4°C, 30 min on room temperature), standard immunostaining for Synaptophysin to detect synapses and determine co-localization with the live tagging Synaptotagmin 1 antibody, embedded in Mowiol |
| imaging setup | Nikon Ti-E, 60x apochromat oil immersion objective |

**Table 21: Synaptic vesicle inactivation is faster under physiological stimulation conditions (relates to Appendix Fig S12).** In this set of experiments, we determined how soon ageing synaptic vesicles stop releasing under physiological stimulation conditions, refining the data from stimulated release (Fig 2). To test this, we performed standard Synaptotagmin 1 live tagging (as described, for example, in Table 1), but with an unconjugated Synaptotagmin 1 antibody. We then performed additional live antibody tagging with a fluorophore-conjugated secondary antibody to reveal the initially tagged Synaptotagmin 1 epitopes that were still active in release and recycling. We found that inactivation was indeed substantially faster under physiological stimulation conditions, and determined that essentially all synaptic vesicles active at the start of the experiment were no longer employed in release under intrinsic network activity stimulation after ~24 h.

| Figure | Appendix Fig S12b,c |
| --- | --- |
| number of experiments | 5 (0 h), 3 (12 h), 4 (24 h), 4 (48 h) independent experiments, >10 neurons imaged per experiment. |
| antibodies used | Synaptotagmin 1 live tagging: Synaptic Systems, 105 311, clone 604.2, against lumenal domain of Synaptotagmin 1, unconjugated  live detection of Synaptotagmin 1 primary antibody via a secondary antibody: Dianova, 115-175-146, anti-mouse, conjugated to Cy5, dialyzed into Tyrode’s solution for application in live cell immunolabeling |
| antibody live tagging | Unconjugated Synaptotagmin 1 antibody was applied (1:120 from 1 mg/ml stock), to live primary hippocampal neurons, in their own culture medium, for 1 h at 37°C in the cell culture incubator. The antibody was then washed off with ice-cold Tyrode’s solution (3-times on/off), and the cultures were maintained in their own culture medium until processing.  Secondary antibody conjugated to Cy5, against the Synaptotagmin 1 antibody, was applied live (at 12 h, 24 h, or 48 h after tagging with the Synaptotagmin 1 antibody), in the neurons’ own culture medium, for 1 h at 37°C in the cell culture incubator), to detect those epitopes still in the recycling pool. Secondary antibody conjugated to Cy3, against the Synaptotagmin 1 antibody, was then applied after fixation and permeabilization to detect inactive epitopes no longer in the recycling pool. |
| description of time course | At time point 0 h, recycling synaptic vesicles were tagged with the unconjugated antibody against Synaptotagmin 1, followed by detection of this primary antibody in the recycling population via live secondary labelling (Cy5; 12 h, 24 h, or 48 h after tagging with the Synaptotagmin 1 antibody). Following fixation and permeabilization, the internalized population of tagged vesicles that was no longer participating in release was revealed using a differently conjugated secondary antibody (Cy3). For time point 0 h, this protocol was altered to exclude the live tagging with the Cy5-conjugated secondary antibody against the Synaptotagmin 1 antibody, which was applied in parallel with the Cy3-conjugated secondary antibody against the Synaptotagmin 1 antibody after fixation and permeabilization; this was done to not cause any delay in live detection of the tagged Synaptotagmin 1 in the recycling population and gain a more reliable baseline fluorescence (all Synaptotagmin 1 epitopes were in the recycling population, so this entire population was considered to be the baseline, independent of the fact that some synaptic vesicles tagged at the beginning of the 1 h live tagging period would inevitably have become inactive by the time the live secondary antibody tagging could have been performed). |
| stimulation paradigm | no external stimulation, only intrinsic network activity of primary hippocampal cultures during live antibody tagging for detection during the time course |
| fixation and processing | 4% PFA (15 min 4°C, 30 min on room temperature), standard immunostaining for Synaptophysin to detect synapses, embedded in Mowiol |
| imaging setup | Leica TCS SP5 (confocal mode), 63x apochromat oil immersion objective |

**Table 22: Young VAMP2 proteins also participate preferentially in release (relates to Appendix Fig S13).** In this set of experiments, we tested the inactivation of synaptic vesicle proteins on an additional candidate, VAMP2. VAMP2 cannot be live tagged with antibodies, as it lacks sufficient intra-vesicular epitopes (as do most synaptic vesicle proteins). We thus devised an alternative method, relying on overexpression of a VAMP2-TEV-SNAPtag construct. This construct carries a TEV cleavage site and a SNAP-tag on its vesicle lumenal domain. It can be covalently conjugated to cell-permeable fluorophores via the self-catalyzing function of the SNAP-tag enzyme. This allows to label VAMP2 populations of different age with sequential pulses of different fluorophores (the first pulse saturates the current population, the second pulse saturates the population produced since the first pulse). We then applied AcTEV protease to the neurons during intrinsic network activity, removing the fluorescent SNAP-tag label from releasing epitopes, and observed the loss in fluorescence from the young and the old labeled VAMP2 populations. We found that the young population loses more fluorescence, indicating that ageing VAMP2 proteins also are located on inactive synaptic vesicles.

| Figure | Appendix Fig S13b,c |
| --- | --- |
| number of experiments | 6 independent experiments for sypHy, 9 independent experiments for sypHy-SNAP25 |
| statistics | Appendix Fig S13c (rightmost panel): the unpaired t-test determined that the difference between the time point before TEV-cleavage and the time point after TEV-cleavage was significant, with p < 0.0001, t(20) = 4.9700. |
| constructs used | sypHy (Synaptophysin coupled to pH-sensitive GFP-variant in an internal lumenal loop)  sypHy-SNAP25 (Synaptophysin coupled to pH-sensitive GFP-variant in an internal lumenal loop and SNAP25, mutated to remove all palmitoylation sites, on the cytoplasmic C-terminus) |
| description of time course | Neurons were transfected with the VAMP2-TEV-SNAPtag construct, and maintained in culture for 3-4 days, until expression was sufficient for conjugation and imaging. The neurons were then subjected to a pulse with the cell-permeable fluorophore TMR-Star, in their own culture medium, for 24 h (to achieve sufficient labelling to detect the signal) at 37°C in the cell culture incubator. TMR-Star was then washed out thoroughly with Tyrode’s solution (5- to 6-times, 1 min each), and the neurons were maintained in culture for another 24 h. After this resting period, in which new, unlabeled VAMP2-TEV-SNAPtag could be produced by the neurons, we applied a second cell-permeable dye, 647-SiR, again for 24 h at 37°C in the cell culture incubator. 647-SiR was then washed out thoroughly with Tyrode’s solution (5- to 6-times, 1 min each), and the neurons were imaged live. During continuous live imaging, AcTEV protease was applied and the change in fluorescence intensity monitored in both fluorophore channels to be compared later. |
| stimulation paradigm | no external stimulation, only intrinsic network activity of primary hippocampal cultures during the time course and during the experiment |
| fixation and processing | no fixation, only live imaging |
| imaging setup | Nikon Ti-E, 60x apochromat oil immersion objective; heating chamber to maintain neurons at 37°C during imaging |

**Table 23: Synaptic activity increases with bicuculline and 8 mM Ca^2+^ treatment.** We incubated cultures with either 25 µM bicuculline or with 8 mM Ca^2+^ for 12 hours, before analyzing their activity. We assessed synaptic activity with Ca^2+^ measurements via GCaMP6 for bicuculline, and with sypHy measurements of synaptic vesicle release during stimulation with 600 AP at 20 Hz for 8 mM Ca^2+^. The reason was that the elevated Ca^2+^ in the 8 mM Ca^2+^ condition interferes with Ca^2+^ imaging, and an exogenous stimulation was necessary to quantify a signal change with sypHy, which is less sensitive than GCaMP6.

| Figure | Appendix Figure S14 |
| --- | --- |
| number of experiments | Ca^2+^ measurements with GCaMP6: 3 independent experiments  measurements of synaptic vesicle release with mOr2-sypHy: 3 independent experiments |
| statistics | Appendix Fig S14c: the unpaired t-test determined that the difference between untreated control and bicuculline treatment was significant, with p = 0.0100, t(4) = 4.6005.  Appendix Fig S14f: the unpaired t-test determined that the difference between untreated control and 8 mM Ca^2+^ treatment was significant, with p = 0.0175, t(4) = 3.9012. |
| constructs used | GCaMP6 (see Appendix Fig S15 and Material and Methods) and sypHy (see Appendix Fig S15 and Material and Methods) |
| description of time course | Neurons were transfected with GCaMP6s and sypHy, and were maintained in culture for 3-4 days, until expression was sufficient for imaging. Prior to imaging, neurons were subject to incubation with the indicated drugs, or were left untouched, as controls. The neurons were then either observed at their intrinsic network activity (for GCaMP6 Ca^2+^ imaging), or were subjected to electrical stimulation in the presence of AP5/CNQX during imaging (for sypHy imaging of synaptic vesicle release, 600 AP at 20 Hz). |
| stimulation paradigm | GCaMP6 Ca^2+^ imaging for bicuculline: no external stimulation, only intrinsic network activity of primary hippocampal cultures during observation of individual bursts during intrinsic network activity  sypHy imaging of synaptic vesicle release for 8 mM Ca^2+^: 600 action potentials delivered at 20 Hz in electrical field stimulation for observation |
| fixation and processing | 600 action potentials delivered at 20 Hz in electrical field stimulation |
| imaging setup | Nikon Ti-E, 60x apochromat oil immersion objective; heating chamber to maintain neurons at 37°C during imaging |

**Table 24: Measurements of synaptic activity with sypHy and GCaMP6 (relates to Appendix Fig S15).** In this set of experiments, we set out to characterize the activity of synapses in our culture system both on the level of synaptic vesicle release (monitored via sypHy) and on the level of synaptic Ca^2+^ influx (monitored via GCaMP6). Our goal was to determine the exact release parameters to calculate the number of release events a synaptic vesicle undergoes throughout its life cycle (see Material and Methods).

| Figure | Ca^2+^ measurements with GCaMP6: Appendix Fig S15a-c  parallel Ca^2+^ measurements with GCaMP6 and synaptic vesicle release measurements with sypHy: Appendix Fig S15d-h |
| --- | --- |
| number of experiments | Ca^2+^ measurements with GCaMP6: 7 independent experiments (Appendix Fig S15c)  parallel Ca^2+^ measurements with GCaMP6 and measurements of synaptic vesicle release with sypHy (Appendix Fig S15h): 4 (both for measuring synaptic vesicle release during individual Ca^2+^ bursts and during 600 action potential stimulation) |
| constructs used | GCaMP6 (Appendix Fig S15a-f,h) and sypHy (Appendix Fig S15d-h) |
| description of time course | Neurons were transfected with either GCaMP6 alone (Appendix Fig S15a-c) or GCaMP6 and sypHy together (Appendix Fig S15 d-h), and maintained in culture for 3-4 days, until expression was sufficient for imaging. The neurons were then subjected to stimulation in the presence of AP5/CNQX during imaging (see below; Appendix Fig S15g, Appendix Fig S15h 600 AP), or were observed at their intrinsic network activity (Appendix Fig S15a-c, Appendix Fig S15d-f, Appendix Fig S15h individual Ca^2+^ bursts). |
| stimulation paradigm | no external stimulation, only intrinsic network activity of primary hippocampal cultures during observation of individual bursts during intrinsic network activity (Appendix Fig S15a-f, Appendix Fig S15h individual Ca^2+^ bursts)  600 action potentials delivered at 20 Hz in electrical field stimulation for observation of synaptic vesicle release in response to stimulation (Appendix Fig S15g, Appendix Fig S15h 600 AP) |
| imaging setup | Nikon Ti-E, 60x apochromat oil immersion objective; heating chamber to maintain neurons at 37°C during imaging |

**Table 25: Additional experiments confirm that SNAP25, but not Syntaxin 1, enters ageing synaptic vesicles (relates to Appendix Fig S16 and Appendix Fig S17b).** In this set of experiments we determined whether Synaptotagmin 1 antibodies co-localize differently with SNAP25 or Syntaxin 1 at 0 or 4 days after application to the neuronal cultures. The antibodies were applied to live primary hippocampal neuron cultures. The cultures were fixed and permeabilized, either immediately or after a 4-day delay, and were then immunostained for the respective markers, and were analyzed by 2-color 3D STED microscopy.

| Figure | Appendix Fig S16, Appendix Fig S17b |
| --- | --- |
| number of experiments | 5 independent datasets from 3 independent cultures, >10 neurons imaged per dataset. Total numbers of vesicles analyzed: SNAP25 day 0: 6564 vesicles; SNAP24 day 4: 4625 vesicles; Syntaxin 1 day 0: 6380 vesicles; Syntaxin 1 day 4: 5690 vesicles. |
| statistics | Appendix Fig S16b: the unpaired t-test determined that the difference between day 0 and day 4 was significant, with p = 0.0008, t(8) = 5.1912.  Appendix Fig S16d: the unpaired t-test determined that the difference between day 0 and day 4 was not significant, with p = 0.4903, t(8) = 0.7229. |
| antibodies used | Synaptotagmin 1 (live-tagging): Synaptic Systems, 105 311AT, clone 604.2, lumenal domain, conjugated to Atto647N  SNAP25 (co-immunostaining): Synaptic Systems, 111 002, rabbit polyclonal  Syntaxin 1 (co-immunostaining): Synaptic Systems, 111 302, rabbit polyclonal  secondary antibodies (co-immunostaining): goat anti-rabbit IgG conjugated to Abberior STAR580 (Abberior, 2-0012-005-8) |
| antibody live tagging | Synaptotagmin 1 antibody was applied (1:120 from 1 mg/ml stock), to live primary hippocampal neurons, in their own culture medium, for 1 h at 37°C in a cell culture incubator. The antibody was then washed off with ice-cold Tyrode’s solution (3-times on/off), and the cultures were fixed and processed, or were returned to the incubator for 4 days, before fixation and processing. |
| description of time course | 0 days or 4 days incubation at 37°C, in the normal culture medium |
| stimulation paradigm | no external stimulation, only intrinsic network activity of primary hippocampal cultures during live antibody tagging and time course |
| fixation and processing | 4% PFA (15 min 4°C, 30 min on room temperature), standard immunostaining for the proteins of interest, embedding in Mowiol |
| imaging setup | Abberior easy3D STED microscope (two-color STED mode), 100x apochromat oil immersion objective |

**Table 26: Targeting Syntaxin 1 to synaptic vesicles increases the amount of SNAP25 on synaptic vesicles (relates to Appendix Fig S19).** In this set of experiments, we determined whether over-expression of sypHy-Syntaxin 1 targets endogenous SNAP25 to synaptic vesicles with increased propensity. We overexpressed the construct and performed co-immunostainings for Synaptophysin, to identify synaptic boutons, and SNAP25, to measure the amount of that protein associated with synaptic vesicles. We used the intensity of the Synaptophysin staining to correct for the amount of synaptic vesicles in the synaptic boutons.

| Figure | Appendix Fig S19 |
| --- | --- |
| number of experiments | 5 independent experiments, with 23,275 vesicles analyzed in total |
| statistics | Appendix Fig S19b: the paired t-test determined that the difference between day 0 and day 4 was significant, with p = 0.0387, t(8) = 2.4707. |
| antibodies used | SNAP25: Synaptic Systems, 111 002, rabbit polyclonal  Synaptophysin: Synaptic Systems, 101 004, guinea pig polyclonal  secondary antibodies: goat anti-rabbit IgG conjugated to Atto647N (Rockland, 611-156-122) and goat anti-guinea pig IgG conjugated to Alexa 488 (Dianova, 706-545-148) |
| constructs used | sypHy-Syntaxin 1 (Synaptophysin coupled to pH-sensitive GFP-variant in an internal lumenal loop, and Syntaxin 1, without the membrane-integration domain and the intra-vesicular domain, on the cytoplasmic C-terminus) |
| stimulation paradigm | no external stimulation, only intrinsic network activity of primary hippocampal cultures |
| fixation and processing | 4% PFA (15 min 4°C, 30 min on room temperature), standard immunostaining for the proteins of interest, embedding in Mowiol |
| imaging setup | Nikon Ti-E, 150x apochromat oil immersion objective, with added 1.5x lens for 150x total magnification |

**Table 27: Prolonged use of aged synaptic vesicles leads to neuronal degradation (relates to Appendix Fig S27).** In this set of experiments, we determined the amount of neuronal degradation observed during overexpression of the CSPα_WT_ and CSPα_mut_ constructs (used in Fig 10; see Table 9), as a measure of neuron health during prolonged use of aged synaptic vesicles. CSPα_WT_ promotes such a prolonged use, while CSPα_mut_ does not (see Fig 10). CSPα_mut_ was used as a control, as expression of any construct can impair neuron health. To observe neuronal morphology, we co-expressed cytosolic GFP (which filled the cell processes, allowing us to observe deteriorations). We found that neuronal degradation was significantly increased during prolonged use of ageing synaptic vesicles (overexpression of CSPα_WT_) compared to overexpression of CSPα_mut_, which did not prolong usage of synaptic vesicles.

| Figure | Appendix Fig S27 |
| --- | --- |
| number of experiments | number of independent experiments (independent experiments, neurons imaged): CSPα_WT_ (4, 17), CSPα_mut_ (5, 8) |
| statistics | Appendix Fig S27b: the unpaired t-test determined that the difference between CSPα_WT_ and CSPα_mut_ was significant, with p = 0.0008, t(8) = 5.1912. |
| constructs used | CSPα_WT_ (wild-type CSPα, mCherry IRES for detection of expression), CSPα_mut_ (CSPα mutated to not target to vesicle membrane, mCherry IRES for detection of expression), GFP (cytosolic) |
| description of time course | Neurons were transfected with the respective constructs and maintained in culture for 3-4 days, until expression was sufficient for imaging. The samples were then fixed, processed, and imaged in parallel. |
| stimulation paradigm | no external stimulation, only intrinsic network activity of primary hippocampal cultures during live antibody tagging |
| fixation and processing | 4% PFA (15 min 4°C, 30 min on room temperature), no additional immunostaining, embedded in Mowiol |
| imaging setup | Leica TCS SP5 (confocal mode), 63x apochromat oil immersion objective |

**Table 28: No changes occur in synaptic activity upon overexpression of CSPα_WT_.** To confirm that over-expression of CSPα_WT_ does not alter synaptic activity, and the increase in the releasable pool of synaptic vesicles we observe in Fig 10d is not simply due to this change in activity, we performed GCaMP6 Ca^2+^ imaging on neurons over-expressing CSPα_WT_. We could not observe any changes in activity burst frequency or the size of the activity bursts.

| Figure | Appendix Fig S28 |
| --- | --- |
| number of experiments | GCaMP6 alone (control): 7 independent experiments  GCaMP6 and CSPα_WT_: 6 independent experiments |
| statistics | Appendix Fig S28g: the unpaired t-test determined that the difference between control and CSPα_WT_ was not significant, with p = 0.5518, t(11) = 0.6138.  Appendix Fig S28h: the unpaired t-test determined that the difference between control and CSPα_WT_ was not significant, with p = 0.9028, t(11) = 0.1249. |
| constructs used | GCaMP6, CSPα_WT_ and IRES-cherry plasmid for control |
| description of time course | Neurons were transfected with both GCaMP6 and CSPα_WT_, or only GCaMP6 (as control), and were maintained in culture for 3-4 days, until expression was sufficient for imaging. The neurons were then observed at their intrinsic network activity (GCaMP6 Ca^2+^ imaging). |
| stimulation paradigm | no external stimulation, only intrinsic network activity of primary hippocampal cultures during observation of individual bursts during intrinsic network activity |
| fixation and processing | live imaging, no fixation or other processing |
| imaging setup | Nikon Ti-E, 60x apochromat oil immersion objective; heating climate chamber to maintain neurons at 37°C during imaging |

**Table 29: Prolonged use of aged synaptic vesicles leads to defects in synaptic vesicle recycling (relates to Appendix Fig S29).** In this set of experiments, we investigated the effectiveness of synaptic vesicles recycling after release during prolonged use of aged synaptic vesicles. We used anisomycin and colchicine to disrupt the supply of newly produced synaptic vesicles (as in Fig 5; also see Table 4). After 24 h of treatment with these drugs (or without treatment, as control), we subjected the neurons to electrical stimulation with 600 action potentials, straining the endocytotic machinery, and monitored the result via live antibody tagging of Synaptotagmin 1. Untreated neurons were able to cope with the demand and efficiently endocytosed all exocytosed molecules, but drugged neurons were unable to do so and Synaptotagmin 1 and Synaptophysin segregated to some extent.

| Figure | Appendix Fig S29 |
| --- | --- |
| number of experiments | 3 independent experiments per data point, >10 neurons sampled per experiment |
| antibodies used | Synaptotagmin 1 live tagging: Synaptic Systems, 105 311AT, clone 604.2, lumenal domain, conjugated to Atto647N |
| antibody live tagging | Synaptotagmin 1 antibody was applied (1:120 from 1 mg/ml stock), to live primary hippocampal neurons, during electrical field stimulation (600 action potentials, delivered at 20 Hz). |
| drug application | anisomycin (40 µM) to inhibit protein biogenesis, or colchicine (10 µM) to disrupt vesicle transport along the microtubule network |
| description of time course | After 24 h of drug treatment (or no treatment, as control), we subjected neurons to stimulation in the presence of the Synaptotagmin 1 antibody for live tagging (600 action potentials, delivered at 20 Hz). We allowed recycling to occur for 5 min before fixation and further processing took place (see table rows below for details) |
| stimulation paradigm | 600 action potentials, delivered at 20 Hz, during live antibody tagging |
| fixation and processing | 4% PFA (15 min 4°C, 30 min on room temperature), standard immunostaining for Synaptophysin to detect synapses, embedded in Mowiol |
| imaging setup | Leica TCS SP5 (confocal mode), 63x apochromat oil immersion objective |

**Table 30: Overexpression of SNAP25 or sypHy-SNAP25 targets synaptic vesicles to late endosomes (relates to Appendix Fig S30).** In this set of experiments, we determined that an increased amount of SNAP25 on synaptic vesicles increases the probability of the vesicle to target to late endosomal degradation or maintenance compartments. We overexpressed either SNAP25 or sypHy-SNAP25 (see Table 8, Fig 9) and performed immunostainings for Rab 7 (as late endosome marker) and Synaptophysin (as synaptic vesicle marker) to determine their co-localization in synapses. For SNAP25, untransfected neurons served as control; for sypHy-SNAP25, neurons transfected with sypHy served as control. We found that there was a significant increase in the association of synaptic vesicles (Synaptophysin) with late endosomes (Rab 7) in both cases (SNAP25 and sypHy-SNAP25 overexpression).

| Figure | Appendix Fig S30 |
| --- | --- |
| number of experiments | over-expression of SNAP25: 10 independent experiments (18 transfected neurons)  over-expression of sypHy or sypHy-SNAP25: 4 (sypHy, 8 neurons) and 4 (sypHy-SNAP25, 14 neurons) independent experiments, respectively |
| statistics | Appendix Fig S30b: unpaired t-tests determined that the difference between control and SNAP25 over-expression was not significant for changes in Synaptophysin, with p = 0.3929, t(34) = 0.8654, but was significant for changes in Rab 7, with p = 0.0296, t(34) = 2.2710.  Appendix Fig S30c: the unpaired t-test determined that the difference between sypHy and sypHy-SNAP25 was significant for changes inRab 7, with p = 0.0415, t(20) = 2.1786. |
| constructs used | sypHy (Synaptophysin coupled to pH-sensitive GFP-variant in an internal lumenal loop)  sypHy-SNAP25 (Synaptophysin coupled to pH-sensitive GFP-variant in an internal lumenal loop and SNAP25, mutated to remove all palmitoylation sites, on the cytoplasmic C-terminus)  YFP-SNAP25 (YFP added for detection of expression) |
| description of time course | Neurons were transfected with either sypHy, sypHy-SNAP25, or SNAP25 and maintained in culture for 3-4 days, until expression was sufficient for imaging. The neurons were then fixed and immunostained for Synaptophysin (synaptic vesicle marker, >95% targeting to synaptic vesicles) and Rab 7 (late endosome marker), and the degree of co-localization of the two targets investigated in confocal microscopy. |
| stimulation paradigm | no external stimulation, only intrinsic network activity of primary hippocampal cultures throughout the experiment |
| fixation and processing | 4% PFA (15 min 4°C, 30 min on room temperature), standard immunostaining for Synaptophysin to detect synapses, embedded in Mowiol |
| imaging setup | Leica TCS SP5 (confocal mode), 63x apochromat oil immersion objective |
