# Supplementary material for: Newly produced synaptic vesicle proteins are preferentially used in synaptic transmission
Source: EMBO J. 2018 Jun 27;37(15):e98044. doi: 10.15252/embj.201798044 (PMC6068464; doi:10.15252/embj.201798044)
Supplement: Supplementary file 2 — Source Data for Appendix [file EMBJ-37-e98044-s011.zip › 180518_Appendix_SourceData/180518_Table11_FigS2.docx]

**Table 11: The Synaptotagmin 1 antibody is taken up in a specific fashion (relates to Appendix Fig S2).** In this set of experiments we determined whether Synaptotagmin 1 antibodies conjugated to Atto647N are taken up more efficiently than non-specific secondary antibodies against mouse IgG conjugated to Atto647N, which do not recognize lumenal epitopes in neurons. The antibodies were applied to live primary hippocampal neuron cultures.

| Figure | Appendix Fig S2 |
| --- | --- |
| number of experiments | 3 independent experiments per condition, >10 neurons imaged per experiment |
| antibodies used | Synaptotagmin 1: Synaptic Systems, 105 311AT, clone 604.2, lumenal domain, conjugated to Atto647N  anti-mouse IgG: Dianova, 115-005-146, goat polyclonal, conjugated to Atto647N |
| antibody live tagging | Synaptotagmin 1 or anti-mouse IgG antibody was applied (1:120 from 1 mg/ml stock), to live primary hippocampal neurons, in their own culture medium, for 1 h at 37°C in a cell culture incubator. The antibody was then washed off with ice-cold Tyrode’s solution (3-times on/off), and the cultures were fixed and processed. |
| stimulation paradigm | No external stimulation, only intrinsic network activity of primary hippocampal cultures during live antibody tagging and time course |
| fixation and processing | 4% PFA (15 min on ice, 30 min on room temperature), standard immunostaining for Synaptophysin to detect synapses, embedded in Mowiol |
| imaging setup | Nikon Ti-E, 60x apochromat oil immersion objective |
