# Supplementary material for: Newly produced synaptic vesicle proteins are preferentially used in synaptic transmission
Source: EMBO J. 2018 Jun 27;37(15):e98044. doi: 10.15252/embj.201798044 (PMC6068464; doi:10.15252/embj.201798044)
Supplement: Supplementary file 2 — Source Data for Appendix [file EMBJ-37-e98044-s011.zip › 180518_Appendix_SourceData/180518_Table12_FigS3.docx]

**Table 12: Synaptotagmin 1 antibodies applied during live tagging do not lose contact to their epitopes for at least 10 days in culture (relates to Appendix Fig S3).** In this set of experiments, we strove to confirm that Synaptotagmin 1 antibodies applied during live tagging do not come off their epitopes during the time course we used in our other experiments. We fixed neurons, applied the antibody, and left the neurons for up to 10 days in the incubator at 37°C, in a pH 5.5 buffer to simulate the synaptic vesicle lumen, with a 100x molar excess of antigenic peptide. There was no detectable loss of antibody.

| Figure | Appendix Fig S3 |
| --- | --- |
| number of experiments | 5 (day 0), 3 (day 1), 3 (day 4), 3 (day 10) independent experiments, >10 neurons imaged per experiment |
| statistics | Appendix Fig S3b: one-way ANOVA determined that no significant differences were present in the data, with p = 0.9945, F(3, 13) = 0.02. |
| antibodies used | Synaptotagmin 1: Synaptic Systems, 105 311AT, clone 604.2, lumenal domain, conjugated to Atto647N |
| description of time course | After fixation and permeabilization (as described below), the Synaptotagmin 1 antibody was applied to the cultures. The fixed and labelled cultures were then maintained until their respective time point (day 0 directly after immunostaining, day 1, day 4, day 10) of processing at 37°C in the cell culture incubator, in a pH 5.5 TES-buffered salt solution (to simulate the intravesicular environment), with a 100x molar excess of antigenic peptide. |
| stimulation paradigm | no stimulation, all stainings performed post-fixation |
| fixation and processing | 4% PFA (15 min 4°C, 30 min on room temperature), 20 min 100 mM NH_4_Cl to quench residual PFA activity, time course as described two table rows above; post-fixation with 4% PFA (15 min 4°C, 30 min on room temperature), standard immunostaining for Synaptophysin to detect synapses, embedded in Mowiol |
| imaging setup | Leica TCS SP5 (confocal mode), 63x apochromat oil immersion objective |
