# Supplementary material for: Newly produced synaptic vesicle proteins are preferentially used in synaptic transmission
Source: EMBO J. 2018 Jun 27;37(15):e98044. doi: 10.15252/embj.201798044 (PMC6068464; doi:10.15252/embj.201798044)
Supplement: Supplementary file 2 — Source Data for Appendix [file EMBJ-37-e98044-s011.zip › 180518_Appendix_SourceData/180518_Table13_FigS4.docx]

**Table 13: Releasability of newly tagged synaptic vesicle proteins in response to stimulation (refers to Appendix Fig S4).** In this set of experiments, we determined the fraction of synaptic vesicle molecules tagged with antibody (as described in Table 1) that are still to exocytose in response to an external stimulus designed to release the entire recycling pool (1200 action potentials at 20 Hz) immediately after tagging.

| Figure | Appendix Fig S4 |
| --- | --- |
| number of experiments | 3 independent experiments, >10 neurons imaged per experiment |
| statistics | Appendix Fig S4b: the paired t-test determined that the release upon stimulation is highly significant, with p < 0.0001, t(2) = 82.1314. |
| antibodies used | Synaptotagmin 1: Synaptic Systems, 105 311CpH, clone 604.2, lumenal domain, conjugated to CypHer5E |
| antibody live tagging | Synaptotagmin 1 antibody was applied (1:120 from 1 mg/ml stock), to live primary hippocampal neurons, in their own culture medium, for 1 h at 37°C in a cell culture incubator. The antibody was then washed off with ice-cold Tyrode’s solution (3-times on/off), and the cultures were maintained in their own culture medium until processing for their respective time point. |
| description of time course | Live tagging of releasing synaptic vesicles was performed (as described in the previous table row), right before processing for the initial time point (day 0). The cultures were then imaged live during stimulation with 1200 action potentials before fixation and imaging in low- and high-pH buffers for normalization (see two table rows below). |
| stimulation paradigm | during live antibody tagging and time course: no external stimulation, only intrinsic network activity of primary hippocampal cultures  during experiment, to test releasability of vesicles: 1200 action potentials delivered at 20 Hz in electrical field stimulation (in Tyrode’s solution with bafilomycin, to prevent re-acidification during imaging) |
| fixation and processing | Fixation was done with methanol (20 min, -20°C) after live imaging (with stimulation) to preserve the pH-sensitivity and fluorescence of CypHer5E, no additional immunostaining. To determine the entire amount of antibodies present in the preparation, an application of pH 5.5 TES buffered salt solution was performed to activate CypHer5E. To determine the background fluorescence, not due to CypHer5E antibodies, an application of pH 7.4 PBS, to quench all CypHer5E molecules, was performed. |
| imaging setup | Nikon Ti-E, 60x apochromat oil immersion objective; heating chamber to maintain neurons at 37°C during imaging |
