# Supplementary material for: Newly produced synaptic vesicle proteins are preferentially used in synaptic transmission
Source: EMBO J. 2018 Jun 27;37(15):e98044. doi: 10.15252/embj.201798044 (PMC6068464; doi:10.15252/embj.201798044)
Supplement: Supplementary file 2 — Source Data for Appendix [file EMBJ-37-e98044-s011.zip › 180518_Appendix_SourceData/180518_Table14_FigS5.docx]

**Table 14: Available epitopes in the recycling population of synaptic vesicles are saturated after 30-60 min of incubation with lumenal domain Synaptotagmin 1 antibodies during intrinsic network activity (relates to Appendix Fig S5).** To determine which incubation time is necessary to tag the entire releasable and recycling population of synaptic vesicles in our primary hippocampal cultures, we incubated them with the lumenal domain Synaptotagmin 1 antibody for varying times and determined that saturation is achieved after 30-60 min. This was necessary to establish the experimental parameters for all other experiments, which rely on complete tagging of the releasable and recycling population of synaptic vesicles. Alternatively, the 60-minute incubated cultures were stimulated at 20 Hz for 30 seconds.

| Figure | Appendix Fig S5 |
| --- | --- |
| number of experiments | 3 independent experiments per time point, >10 neurons imaged per data point |
| antibodies used | Synaptotagmin 1: Synaptic Systems, 105 311AT, clone 604.2, lumenal domain, conjugated to Atto647N |
| antibody live  tagging | Synaptotagmin 1 antibody was applied (1:120 from 1 mg/ml stock), to live primary hippocampal neurons, in their own culture medium, for 1 min, 10 min, 30 min, or 6 0 min at 37°C in a cell culture incubator. The antibody was then washed off with Tyrode’s solution (3-times on/off), and the cultures were maintained in their own culture medium until processing for their respective time point. |
| description of conditions | Live-tagging with the Synaptotagmin 1 was allowed to proceed for 1 min, 10 min, 30 min, or 60 min to determine after what time saturation of all releasable epitopes would be achieved during intrinsic network activity. |
| stimulation paradigm | a-b: no external stimulation, only intrinsic network activity of primary hippocampal cultures during live antibody tagging and time course  c-d: 20 Hz, 30 seconds electrical stimulation |
| fixation and processing | 4% PFA (15 min 4°C, 30 min on room temperature), standard immunostaining for Synaptophysin to detect synapses and determine co-localization with the live tagging Synaptotagmin 1 antibody, embedded in Mowiol |
| imaging setup | a-b: Cytation 3 cell imaging, multi-mode reader (BioTek), equipped with a 20x objective  c-d: Nikon Ti-E epifluorescence microscope, equipped with a 100x objective |
