# Supplementary material for: Newly produced synaptic vesicle proteins are preferentially used in synaptic transmission
Source: EMBO J. 2018 Jun 27;37(15):e98044. doi: 10.15252/embj.201798044 (PMC6068464; doi:10.15252/embj.201798044)
Supplement: Supplementary file 2 — Source Data for Appendix [file EMBJ-37-e98044-s011.zip › 180518_Appendix_SourceData/180518_Table15_FigS6.docx]

**Table 15: The Synaptotagmin 1 antibody is taken up in synaptic vesicles (relates to Appendix Fig S6).** In this set of experiments we determined whether Synaptotagmin 1 antibodies are taken up in organelles that co-localize with the synaptic vesicle marker Synaptophysin, with the dense-core vesicle marker Chromogranin A, or with the endosomal markers Rab 5 and Rab 7. The Synaptotagmin 1 lumenal antibody was applied to live primary hippocampal neuron cultures. Following 1 h incubation at 37°C, the cultures were fixed, permeabilized and immunostained for the respective markers. Samples were analyzed by 2-color 3D STED microscopy.

| Figure | Appendix Fig S6 |
| --- | --- |
| number of experiments | 3 independent experiments, >10 neurons imaged per experiment, >1000 vesicles analyzed per experiment. |
| statistics | Appendix Fig S6b: one-way ANOVA indicated that significant differences were present in the data, with p < 0.0001, F(2, 12) = 332.86. Significant differences were found with the post-hoc Bonferroni procedure between the random data (negative control) and Synaptotagmin 1 (p < 0.0001) as well as between the random data (negative control) and Synaptophysin (p < 0.0001). All other comparisons were not significant.  Appendix Fig S6d: the unpaired t-test determined that there are no significant differences between the protein of interest and random data in association with Synaptotagmin 1 live tagging, with p = 0.9999, t(20) = 0.0001.  Appendix Fig S6e: the unpaired t-test determined that there are no significant differences between the protein of interest and random data in association with Synaptotagmin 1 live tagging, with p = 0.9999, t(20) < 0.0001.  Appendix Fig S6f: the unpaired t-test determined that there are no significant differences between the protein of interest and random data in association with Synaptotagmin 1 live tagging, with p = 0.9999, t(20) < 0.0001. |
| antibodies used | Synaptotagmin 1, for live-tagging: Synaptic Systems, 105 311AT, clone 604.2, lumenal domain, conjugated to Atto647N  Synaptophysin 1 (co-immunostaining): Synaptic Systems, 101 004, guinea pig polyclonal  Chromogranin A (co-immunostaining): Synaptic Systems, 259 003, rabbit polyclonal  Rab 5 (co-immunostaining): Cell Signaling, 3547, rabbit polyclonal  Rab 7 (co-immunostaining): Cell Signaling, 9367, rabbit polyclonal  secondary antibodies (co-immunostaining): goat anti-guinea pig IgG conjugated to Abberior STAR580 (Abberior, 2-0112-005-7) or goat anti-rabbit IgG conjugated to Abberior STAR580 (Abberior, 2-0012-005-8) |
| antibody live tagging | Synaptotagmin 1 antibody was applied (1:120 from 1 mg/ml stock), to live primary hippocampal neurons, in their own culture medium, for 1 h at 37°C in a cell culture incubator. The antibody was then washed off with ice-cold Tyrode’s solution (3-times on/off), and the cultures were fixed and processed. |
| stimulation paradigm | No external stimulation, only intrinsic network activity of primary hippocampal cultures during live antibody tagging and time course |
| fixation and processing | 4% PFA (15 min 4°C, 30 min on room temperature), 20 min 100 mM NH_4_^+^ to quench residual PFA activity, standard immunostaining for the proteins of interest, embedded in Mowiol |
| imaging setup | Abberior easy3D STED microscope (two-color STED mode), 100x apochromat oil immersion objective |
