# Supplementary material for: Newly produced synaptic vesicle proteins are preferentially used in synaptic transmission
Source: EMBO J. 2018 Jun 27;37(15):e98044. doi: 10.15252/embj.201798044 (PMC6068464; doi:10.15252/embj.201798044)
Supplement: Supplementary file 2 — Source Data for Appendix [file EMBJ-37-e98044-s011.zip › 180518_Appendix_SourceData/180518_Table16_FigS7.docx]

**Table 16: Investigation of the synaptic vesicle populations marked by antibody live tagging (relates to Appendix Fig S7).** In this set of experiments, we tested to which functional populations of synaptic vesicles the Synaptotagmin 1 antibody used for live tagging binds to. We found that it labels the surface pool (~27% of all epitopes), the spontaneously releasing pool of synaptic vesicles (~4% of all epitopes), and the recycling pool (~22% of all epitopes). The reserve pool (~47% of all epitopes) is not labelled by live cell tagging.

| Figure | Appendix Fig S7 |
| --- | --- |
| number of experiments | 3 independent experiments per condition, >10 neurons sampled per experiment |
| antibodies used | Synaptotagmin 1: Synaptic Systems, 105 311AT, clone 604.2, lumenal domain, conjugated to Atto647N |
| antibody live  tagging | Synaptotagmin 1 antibody was applied (1:120 from 1 mg/ml stock), to live primary hippocampal neurons, in their own culture medium, as described in the table row below for the different conditions. The antibody was then washed off with ice-cold Tyrode’s solution (3-times on/off), and the cultures were fixed and processed immediately as described below. |
| description of conditions | Live tagging of Synaptotagmin 1 (standard conditions, as used in most experiments here): 1 h of live tagging (as described in the table row above) at 37°C in a cell culture incubator.  Additional tagging of Synaptotagmin 1 after fixation and permeabilization: as above, but after fixation and permeabilization we performed an additional immunostaining for Synaptotagmin 1 with the same antibody to reveal all Synaptotagmin 1 epitopes (surface, recycling, and internalized). This was performed on the same coverslips used in the condition above, after cutting them in half after fixation (one half was treated as described in this condition, the other half was not subjected to an additional Synaptotagmin 1 staining).  Live tagging of Synaptotagmin 1 in TTX (0.5-1 µM): 1 h of live tagging (as described in the table row above) at 37°C in a cell culture incubator.  Live tagging of Synaptotagmin 1 on 4°C: 1 h of live tagging (as described in the table row above) at 4°C, with TTX (0.5-1 µM) added. |
| stimulation paradigm | no external stimulation, only intrinsic network activity of primary hippocampal cultures during live antibody tagging (or inhibition of activity through TTX or 4°C as described in the table row above). |
| fixation and processing | 4% PFA (15 min 4°C, 30 min on room temperature), standard immunostaining for Synaptophysin to detect synapses, embedded in Mowiol |
| imaging setup | Leica TCS SP5 (confocal mode), 63x apochromat oil immersion objective |
