# Supplementary material for: Newly produced synaptic vesicle proteins are preferentially used in synaptic transmission
Source: EMBO J. 2018 Jun 27;37(15):e98044. doi: 10.15252/embj.201798044 (PMC6068464; doi:10.15252/embj.201798044)
Supplement: Supplementary file 2 — Source Data for Appendix [file EMBJ-37-e98044-s011.zip › 180518_Appendix_SourceData/180518_Table17_FigS8.docx]

**Table 17: Synaptotagmin 1 antibodies applied during live tagging stay within synapses for up to 10 days in culture (relates to Appendix Fig S8).** In this set of experiments, we followed the association of the live antibody tag for Synaptotagmin 1 with synaptic vesicles throughout a time course of up to 10 days after live tagging. To do this, we live-tagged Synaptotagmin 1 with antibodies, as described in Table 1, and performed a co-immunostaining for Synaptophysin (as synaptic vesicle marker) after fixation of the cultures at each respective time point. We found that the association of the Synaptotagmin 1 antibody live tag did not significantly decrease, even after 10 days in culture.

| Figure | Appendix Fig S8 |
| --- | --- |
| number of experiments | 3 (day 0), 3 (day 1), 2 (day 2), 3 (day 4), 3 (day 7), 2 (day 10) independent experiments, >10 neurons imaged per experiment |
| statistics | Appendix Fig S8b: one-way ANOVA determined that no significant differences were present in the data, with p = 0.8582, F(5, 15) = 0.37. |
| antibodies used | Synaptotagmin 1: Synaptic Systems, 105 311AT, clone 604.2, lumenal domain, conjugated to Atto647N |
| antibody live  tagging | Synaptotagmin 1 antibody was applied (1:120 from 1 mg/ml stock), to live primary hippocampal neurons, in their own culture medium, for 1 h at 37°C in a cell culture incubator. The antibody was then washed off with ice-cold Tyrode’s solution (3-times on/off), and the cultures were maintained in their own culture medium until processing for their respective time point. |
| description of time course | Live tagging of releasing synaptic vesicles was performed (as described in the previous table row), right before processing for the initial time point (day 0). Separate cultures for each time point (day 0, day 1, day 2, day 4, day 7, day 10) were pulsed in parallel and maintained in the incubator until processing. |
| stimulation paradigm | no external stimulation, only intrinsic network activity of primary hippocampal cultures during live antibody tagging and time course. |
| fixation and processing | 4% PFA (15 min 4°C, 30 min on room temperature), standard immunostaining for Synaptophysin to detect synapses and determine co-localization with the live tagging Synaptotagmin 1 antibody, embedded in Mowiol |
| imaging setup | Leica TCS SP5 (confocal mode), 63x apochromat oil immersion objective |
