# Supplementary material for: Newly produced synaptic vesicle proteins are preferentially used in synaptic transmission
Source: EMBO J. 2018 Jun 27;37(15):e98044. doi: 10.15252/embj.201798044 (PMC6068464; doi:10.15252/embj.201798044)
Supplement: Supplementary file 2 — Source Data for Appendix [file EMBJ-37-e98044-s011.zip › 180518_Appendix_SourceData/180518_Table19_FigS9c.docx]

**Table 19: Inhibition of lysosome activity delays loss of live tagged Synaptotagmin 1 (relates to Appendix Fig S9c).** In this set of experiments, we tested whether the live tagging Synaptotagmin 1 antibody is degraded via lysosomes. We performed live antibody tagging and inhibited lysosomal activity for 24 h, and compared the amount of fluorescence intensity in synapses to a control condition where lysosomal activity was not inhibited. We found that there was significantly more fluorescence signal remaining in neuros where lysosomal activity was inhibited.

| Figure | Appendix Fig S9c |
| --- | --- |
| number of experiments | 3 independent experiments per data point, >10 neurons imaged per experiment |
| statistics | The unpaired t-test determined that the difference between the untreated control condition and the condition treated with leupeptin was significant, with p = 0.0237, t(4) = 3.5561. |
| antibodies used | Synaptotagmin 1: Synaptic Systems, 105 311AT, lumenal domain, conjugated to Atto647N |
| antibody live tagging | Synaptotagmin 1 antibody was applied (1:120 from 1 mg/ml stock), to live primary hippocampal neurons, in their own culture medium, for 1 h at 37°C in a cell culture incubator. The antibody was then washed off with ice-cold Tyrode’s solution (3-times on/off), and the cultures were maintained in their own culture medium until processing for their respective time point. |
| drug application | leupeptin (100 µM) to inhibit lysosomal enzymes |
| description of time course | Live tagging of releasing synaptic vesicles was performed (as described above). The cultures were then maintained for 24 h in the incubator, either without treatment or with the leupeptin treatment described in the table row above. After 24 h, the cultures were processed (see two table rows below), imaged, and compared. |
| stimulation paradigm | no external stimulation, only intrinsic network activity of primary hippocampal cultures |
| fixation and processing | 4% PFA (15 min 4°C, 30 min on room temperature), standard immunostaining for Synaptophysin to detect synapses, embedded in Mowiol |
| imaging setup | Leica TCS SP5 (confocal mode), 63x apochromat oil immersion objective |
