# Supplementary material for: Newly produced synaptic vesicle proteins are preferentially used in synaptic transmission
Source: EMBO J. 2018 Jun 27;37(15):e98044. doi: 10.15252/embj.201798044 (PMC6068464; doi:10.15252/embj.201798044)
Supplement: Supplementary file 2 — Source Data for Appendix [file EMBJ-37-e98044-s011.zip › 180518_Appendix_SourceData/180518_Table20_FigS10_FigS11.docx]

**Table 20: Neurons of different ages show similar levels of synaptic activity, and similar synapses (relates to Appendix Figs S10 and S11).** To determine whether the neurons change during the long experiments, we incubated cultures with Synaptotagmin 1 antibodies at different days *in vitro*, and we also immunostained them for Synaptophysin, to determine their synapse morphologies.

| Figure | Appendix Fig S10, Appendix Fig S11 |
| --- | --- |
| number of experiments | 3 independent experiments per time point, >10 neurons imaged per data point |
| statistics | Appendix Fig S10b: one-way ANOVA determined that no significant differences were present in the data, with p = 0.3046, F(5, 17) = 1.36.  Appendix Fig S10b-g: one-way ANOVAs determined that no significant differences were present in the data, with (b) p = 0.4743, F(5, 17) = 0.97; (c) p = 0.3046, F(5, 17) = 1.36; (d) p = 0.6040, F(5, 17) = 0.75; (e) p = 0.6899, F(5, 17) = 0.62; (f) p = 0.3462, F(5, 17) = 1.25; (g) p = 0.7542, F(5, 17) = 0.52. |
| antibodies used | Synaptotagmin 1 (live-tagging): Synaptic Systems, 105 311AT, clone 604.2, lumenal domain, conjugated to Atto647N  Synaptophysin (co-immunostaining): Synaptic Systems, 101 004, guinea pig polyclonal  secondary antibody (co-immunostaining): goat anti-guinea pig conjugated to Abberior STAR580 (Abberior, 2-0112-005-7) |
| antibody live  tagging | Synaptotagmin 1 antibody was applied (1:120 from 1 mg/ml stock), to live primary hippocampal neurons, in their own culture medium, for 60 min at 37°C in a cell culture incubator. The antibody was then washed off with Tyrode’s solution (3-times on/off), and the cultures were fixed and processed. |
| description of conditions | Live-tagging with the Synaptotagmin 1 was allowed to proceed for 1 hour, before fixation and immunostaining for Synaptophysin, in cultures of different ages (days in vitro). |
| stimulation paradigm | no external stimulation, only intrinsic network activity of primary hippocampal cultures during live antibody tagging and time course |
| fixation and processing | 4% PFA (15 min 4°C, 30 min on room temperature), standard immunostaining for Synaptophysin to detect synapses and determine co-localization with the live tagging Synaptotagmin 1 antibody, embedded in Mowiol |
| imaging setup | Nikon Ti-E, 60x apochromat oil immersion objective |
