# Supplementary material for: Newly produced synaptic vesicle proteins are preferentially used in synaptic transmission
Source: EMBO J. 2018 Jun 27;37(15):e98044. doi: 10.15252/embj.201798044 (PMC6068464; doi:10.15252/embj.201798044)
Supplement: Supplementary file 2 — Source Data for Appendix [file EMBJ-37-e98044-s011.zip › 180518_Appendix_SourceData/180518_Table23_FigS14.docx]

**Table 23: Synaptic activity increases with bicuculline and 8 mM Ca^2+^ treatment.** We incubated cultures with either 25 µM bicuculline or with 8 mM Ca^2+^ for 12 hours, before analyzing their activity. We assessed synaptic activity with Ca^2+^ measurements via GCaMP6 for bicuculline, and with sypHy measurements of synaptic vesicle release during stimulation with 600 AP at 20 Hz for 8 mM Ca^2+^. The reason was that the elevated Ca^2+^ in the 8 mM Ca^2+^ condition interferes with Ca^2+^ imaging, and an exogenous stimulation was necessary to quantify a signal change with sypHy, which is less sensitive than GCaMP6.

| Figure | Appendix Figure S14 |
| --- | --- |
| number of experiments | Ca^2+^ measurements with GCaMP6: 3 independent experiments  measurements of synaptic vesicle release with mOr2-sypHy: 3 independent experiments |
| statistics | Appendix Fig S14c: the unpaired t-test determined that the difference between untreated control and bicuculline treatment was significant, with p = 0.0100, t(4) = 4.6005.  Appendix Fig S14f: the unpaired t-test determined that the difference between untreated control and 8 mM Ca^2+^ treatment was significant, with p = 0.0175, t(4) = 3.9012. |
| constructs used | GCaMP6 (see Appendix Fig S15 and Material and Methods) and sypHy (see Appendix Fig S15 and Material and Methods) |
| description of time course | Neurons were transfected with GCaMP6s and sypHy, and were maintained in culture for 3-4 days, until expression was sufficient for imaging. Prior to imaging, neurons were subject to incubation with the indicated drugs, or were left untouched, as controls. The neurons were then either observed at their intrinsic network activity (for GCaMP6 Ca^2+^ imaging), or were subjected to electrical stimulation in the presence of AP5/CNQX during imaging (for sypHy imaging of synaptic vesicle release, 600 AP at 20 Hz). |
| stimulation paradigm | GCaMP6 Ca^2+^ imaging for bicuculline: no external stimulation, only intrinsic network activity of primary hippocampal cultures during observation of individual bursts during intrinsic network activity  sypHy imaging of synaptic vesicle release for 8 mM Ca^2+^: 600 action potentials delivered at 20 Hz in electrical field stimulation for observation |
| fixation and processing | 600 action potentials delivered at 20 Hz in electrical field stimulation |
| imaging setup | Nikon Ti-E, 60x apochromat oil immersion objective; heating chamber to maintain neurons at 37°C during imaging |
