# Supplementary material for: Newly produced synaptic vesicle proteins are preferentially used in synaptic transmission
Source: EMBO J. 2018 Jun 27;37(15):e98044. doi: 10.15252/embj.201798044 (PMC6068464; doi:10.15252/embj.201798044)
Supplement: Supplementary file 2 — Source Data for Appendix [file EMBJ-37-e98044-s011.zip › 180518_Appendix_SourceData/180518_Table24_FigS15.docx]

**Table 24: Measurements of synaptic activity with sypHy and GCaMP6 (relates to Appendix Fig S15).** In this set of experiments, we set out to characterize the activity of synapses in our culture system both on the level of synaptic vesicle release (monitored via sypHy) and on the level of synaptic Ca^2+^ influx (monitored via GCaMP6). Our goal was to determine the exact release parameters to calculate the number of release events a synaptic vesicle undergoes throughout its life cycle (see Material and Methods).

| Figure | Ca^2+^ measurements with GCaMP6: Appendix Fig S15a-c  parallel Ca^2+^ measurements with GCaMP6 and synaptic vesicle release measurements with sypHy: Appendix Fig S15d-h |
| --- | --- |
| number of experiments | Ca^2+^ measurements with GCaMP6: 7 independent experiments (Appendix Fig S15c)  parallel Ca^2+^ measurements with GCaMP6 and measurements of synaptic vesicle release with sypHy (Appendix Fig S15h): 4 (both for measuring synaptic vesicle release during individual Ca^2+^ bursts and during 600 action potential stimulation) |
| constructs used | GCaMP6 (Appendix Fig S15a-f,h) and sypHy (Appendix Fig S15d-h) |
| description of time course | Neurons were transfected with either GCaMP6 alone (Appendix Fig S15a-c) or GCaMP6 and sypHy together (Appendix Fig S15 d-h), and maintained in culture for 3-4 days, until expression was sufficient for imaging. The neurons were then subjected to stimulation in the presence of AP5/CNQX during imaging (see below; Appendix Fig S15g, Appendix Fig S15h 600 AP), or were observed at their intrinsic network activity (Appendix Fig S15a-c, Appendix Fig S15d-f, Appendix Fig S15h individual Ca^2+^ bursts). |
| stimulation paradigm | no external stimulation, only intrinsic network activity of primary hippocampal cultures during observation of individual bursts during intrinsic network activity (Appendix Fig S15a-f, Appendix Fig S15h individual Ca^2+^ bursts)  600 action potentials delivered at 20 Hz in electrical field stimulation for observation of synaptic vesicle release in response to stimulation (Appendix Fig S15g, Appendix Fig S15h 600 AP) |
| imaging setup | Nikon Ti-E, 60x apochromat oil immersion objective; heating chamber to maintain neurons at 37°C during imaging |
