# Supplementary material for: Newly produced synaptic vesicle proteins are preferentially used in synaptic transmission
Source: EMBO J. 2018 Jun 27;37(15):e98044. doi: 10.15252/embj.201798044 (PMC6068464; doi:10.15252/embj.201798044)
Supplement: Supplementary file 2 — Source Data for Appendix [file EMBJ-37-e98044-s011.zip › 180518_Appendix_SourceData/180518_Table26_FigS19.docx]

**Table 26: Targeting Syntaxin 1 to synaptic vesicles increases the amount of SNAP25 on synaptic vesicles (relates to Appendix Fig S19).** In this set of experiments, we determined whether over-expression of sypHy-Syntaxin 1 targets endogenous SNAP25 to synaptic vesicles with increased propensity. We overexpressed the construct and performed co-immunostainings for Synaptophysin, to identify synaptic boutons, and SNAP25, to measure the amount of that protein associated with synaptic vesicles. We used the intensity of the Synaptophysin staining to correct for the amount of synaptic vesicles in the synaptic boutons.

| Figure | Appendix Fig S19 |
| --- | --- |
| number of experiments | 5 independent experiments, with 23,275 vesicles analyzed in total |
| statistics | Appendix Fig S19b: the paired t-test determined that the difference between day 0 and day 4 was significant, with p = 0.0387, t(8) = 2.4707. |
| antibodies used | SNAP25: Synaptic Systems, 111 002, rabbit polyclonal  Synaptophysin: Synaptic Systems, 101 004, guinea pig polyclonal  secondary antibodies: goat anti-rabbit IgG conjugated to Atto647N (Rockland, 611-156-122) and goat anti-guinea pig IgG conjugated to Alexa 488 (Dianova, 706-545-148) |
| constructs used | sypHy-Syntaxin 1 (Synaptophysin coupled to pH-sensitive GFP-variant in an internal lumenal loop, and Syntaxin 1, without the membrane-integration domain and the intra-vesicular domain, on the cytoplasmic C-terminus) |
| stimulation paradigm | no external stimulation, only intrinsic network activity of primary hippocampal cultures |
| fixation and processing | 4% PFA (15 min 4°C, 30 min on room temperature), standard immunostaining for the proteins of interest, embedding in Mowiol |
| imaging setup | Nikon Ti-E, 150x apochromat oil immersion objective, with added 1.5x lens for 150x total magnification |
