# Supplementary material for: Newly produced synaptic vesicle proteins are preferentially used in synaptic transmission
Source: EMBO J. 2018 Jun 27;37(15):e98044. doi: 10.15252/embj.201798044 (PMC6068464; doi:10.15252/embj.201798044)
Supplement: Supplementary file 2 — Source Data for Appendix [file EMBJ-37-e98044-s011.zip › 180518_Appendix_SourceData/180518_Table27_FigS27.docx]

**Table 27: Prolonged use of aged synaptic vesicles leads to neuronal degradation (relates to Appendix Fig S27).** In this set of experiments, we determined the amount of neuronal degradation observed during overexpression of the CSPα_WT_ and CSPα_mut_ constructs (used in Fig 10; see Table 9), as a measure of neuron health during prolonged use of aged synaptic vesicles. CSPα_WT_ promotes such a prolonged use, while CSPα_mut_ does not (see Fig 10). CSPα_mut_ was used as a control, as expression of any construct can impair neuron health. To observe neuronal morphology, we co-expressed cytosolic GFP (which filled the cell processes, allowing us to observe deteriorations). We found that neuronal degradation was significantly increased during prolonged use of ageing synaptic vesicles (overexpression of CSPα_WT_) compared to overexpression of CSPα_mut_, which did not prolong usage of synaptic vesicles.

| Figure | Appendix Fig S27 |
| --- | --- |
| number of experiments | number of independent experiments (independent experiments, neurons imaged): CSPα_WT_ (4, 17), CSPα_mut_ (5, 8) |
| statistics | Appendix Fig S27b: the unpaired t-test determined that the difference between CSPα_WT_ and CSPα_mut_ was significant, with p = 0.0008, t(8) = 5.1912. |
| constructs used | CSPα_WT_ (wild-type CSPα, mCherry IRES for detection of expression), CSPα_mut_ (CSPα mutated to not target to vesicle membrane, mCherry IRES for detection of expression), GFP (cytosolic) |
| description of time course | Neurons were transfected with the respective constructs and maintained in culture for 3-4 days, until expression was sufficient for imaging. The samples were then fixed, processed, and imaged in parallel. |
| stimulation paradigm | no external stimulation, only intrinsic network activity of primary hippocampal cultures during live antibody tagging |
| fixation and processing | 4% PFA (15 min 4°C, 30 min on room temperature), no additional immunostaining, embedded in Mowiol |
| imaging setup | Leica TCS SP5 (confocal mode), 63x apochromat oil immersion objective |
