# Supplementary material for: Newly produced synaptic vesicle proteins are preferentially used in synaptic transmission
Source: EMBO J. 2018 Jun 27;37(15):e98044. doi: 10.15252/embj.201798044 (PMC6068464; doi:10.15252/embj.201798044)
Supplement: Supplementary file 2 — Source Data for Appendix [file EMBJ-37-e98044-s011.zip › 180518_Appendix_SourceData/180518_Table28_FigS28.docx]

**Table 28: No changes occur in synaptic activity upon overexpression of CSPα_WT_.** To confirm that over-expression of CSPα_WT_ does not alter synaptic activity, and the increase in the releasable pool of synaptic vesicles we observe in Fig 10d is not simply due to this change in activity, we performed GCaMP6 Ca^2+^ imaging on neurons over-expressing CSPα_WT_. We could not observe any changes in activity burst frequency or the size of the activity bursts.

| Figure | Appendix Fig S28 |
| --- | --- |
| number of experiments | GCaMP6 alone (control): 7 independent experiments  GCaMP6 and CSPα_WT_: 6 independent experiments |
| statistics | Appendix Fig S28g: the unpaired t-test determined that the difference between control and CSPα_WT_ was not significant, with p = 0.5518, t(11) = 0.6138.  Appendix Fig S28h: the unpaired t-test determined that the difference between control and CSPα_WT_ was not significant, with p = 0.9028, t(11) = 0.1249. |
| constructs used | GCaMP6, CSPα_WT_ and IRES-cherry plasmid for control |
| description of time course | Neurons were transfected with both GCaMP6 and CSPα_WT_, or only GCaMP6 (as control), and were maintained in culture for 3-4 days, until expression was sufficient for imaging. The neurons were then observed at their intrinsic network activity (GCaMP6 Ca^2+^ imaging). |
| stimulation paradigm | no external stimulation, only intrinsic network activity of primary hippocampal cultures during observation of individual bursts during intrinsic network activity |
| fixation and processing | live imaging, no fixation or other processing |
| imaging setup | Nikon Ti-E, 60x apochromat oil immersion objective; heating climate chamber to maintain neurons at 37°C during imaging |
