# Supplementary material for: Newly produced synaptic vesicle proteins are preferentially used in synaptic transmission
Source: EMBO J. 2018 Jun 27;37(15):e98044. doi: 10.15252/embj.201798044 (PMC6068464; doi:10.15252/embj.201798044)
Supplement: Supplementary file 2 — Source Data for Appendix [file EMBJ-37-e98044-s011.zip › 180518_Appendix_SourceData/180518_Table29_FigS29.docx]

**Table 29: Prolonged use of aged synaptic vesicles leads to defects in synaptic vesicle recycling (relates to Appendix Fig S29).** In this set of experiments, we investigated the effectiveness of synaptic vesicles recycling after release during prolonged use of aged synaptic vesicles. We used anisomycin and colchicine to disrupt the supply of newly produced synaptic vesicles (as in Fig 5; also see Table 4). After 24 h of treatment with these drugs (or without treatment, as control), we subjected the neurons to electrical stimulation with 600 action potentials, straining the endocytotic machinery, and monitored the result via live antibody tagging of Synaptotagmin 1. Untreated neurons were able to cope with the demand and efficiently endocytosed all exocytosed molecules, but drugged neurons were unable to do so and Synaptotagmin 1 and Synaptophysin segregated to some extent.

| Figure | Appendix Fig S29 |
| --- | --- |
| number of experiments | 3 independent experiments per data point, >10 neurons sampled per experiment |
| antibodies used | Synaptotagmin 1 live tagging: Synaptic Systems, 105 311AT, clone 604.2, lumenal domain, conjugated to Atto647N |
| antibody live tagging | Synaptotagmin 1 antibody was applied (1:120 from 1 mg/ml stock), to live primary hippocampal neurons, during electrical field stimulation (600 action potentials, delivered at 20 Hz). |
| drug application | anisomycin (40 µM) to inhibit protein biogenesis, or colchicine (10 µM) to disrupt vesicle transport along the microtubule network |
| description of time course | After 24 h of drug treatment (or no treatment, as control), we subjected neurons to stimulation in the presence of the Synaptotagmin 1 antibody for live tagging (600 action potentials, delivered at 20 Hz). We allowed recycling to occur for 5 min before fixation and further processing took place (see table rows below for details) |
| stimulation paradigm | 600 action potentials, delivered at 20 Hz, during live antibody tagging |
| fixation and processing | 4% PFA (15 min 4°C, 30 min on room temperature), standard immunostaining for Synaptophysin to detect synapses, embedded in Mowiol |
| imaging setup | Leica TCS SP5 (confocal mode), 63x apochromat oil immersion objective |
