# Supplementary material for: Newly produced synaptic vesicle proteins are preferentially used in synaptic transmission
Source: EMBO J. 2018 Jun 27;37(15):e98044. doi: 10.15252/embj.201798044 (PMC6068464; doi:10.15252/embj.201798044)
Supplement: Supplementary file 2 — Source Data for Appendix [file EMBJ-37-e98044-s011.zip › 180518_Appendix_SourceData/180518_Table30_FigS30.docx]

**Table 30: Overexpression of SNAP25 or sypHy-SNAP25 targets synaptic vesicles to late endosomes (relates to Appendix Fig S30).** In this set of experiments, we determined that an increased amount of SNAP25 on synaptic vesicles increases the probability of the vesicle to target to late endosomal degradation or maintenance compartments. We overexpressed either SNAP25 or sypHy-SNAP25 (see Table 8, Fig 9) and performed immunostainings for Rab 7 (as late endosome marker) and Synaptophysin (as synaptic vesicle marker) to determine their co-localization in synapses. For SNAP25, untransfected neurons served as control; for sypHy-SNAP25, neurons transfected with sypHy served as control. We found that there was a significant increase in the association of synaptic vesicles (Synaptophysin) with late endosomes (Rab 7) in both cases (SNAP25 and sypHy-SNAP25 overexpression).

| Figure | Appendix Fig S30 |
| --- | --- |
| number of experiments | over-expression of SNAP25: 10 independent experiments (18 transfected neurons)  over-expression of sypHy or sypHy-SNAP25: 4 (sypHy, 8 neurons) and 4 (sypHy-SNAP25, 14 neurons) independent experiments, respectively |
| statistics | Appendix Fig S30b: unpaired t-tests determined that the difference between control and SNAP25 over-expression was not significant for changes in Synaptophysin, with p = 0.3929, t(34) = 0.8654, but was significant for changes in Rab 7, with p = 0.0296, t(34) = 2.2710.  Appendix Fig S30c: the unpaired t-test determined that the difference between sypHy and sypHy-SNAP25 was significant for changes inRab 7, with p = 0.0415, t(20) = 2.1786. |
| constructs used | sypHy (Synaptophysin coupled to pH-sensitive GFP-variant in an internal lumenal loop)  sypHy-SNAP25 (Synaptophysin coupled to pH-sensitive GFP-variant in an internal lumenal loop and SNAP25, mutated to remove all palmitoylation sites, on the cytoplasmic C-terminus)  YFP-SNAP25 (YFP added for detection of expression) |
| description of time course | Neurons were transfected with either sypHy, sypHy-SNAP25, or SNAP25 and maintained in culture for 3-4 days, until expression was sufficient for imaging. The neurons were then fixed and immunostained for Synaptophysin (synaptic vesicle marker, >95% targeting to synaptic vesicles) and Rab 7 (late endosome marker), and the degree of co-localization of the two targets investigated in confocal microscopy. |
| stimulation paradigm | no external stimulation, only intrinsic network activity of primary hippocampal cultures throughout the experiment |
| fixation and processing | 4% PFA (15 min 4°C, 30 min on room temperature), standard immunostaining for Synaptophysin to detect synapses, embedded in Mowiol |
| imaging setup | Leica TCS SP5 (confocal mode), 63x apochromat oil immersion objective |
