# Supplementary material for: Newly produced synaptic vesicle proteins are preferentially used in synaptic transmission
Source: EMBO J. 2018 Jun 27;37(15):e98044. doi: 10.15252/embj.201798044 (PMC6068464; doi:10.15252/embj.201798044)
Supplement: Supplementary file 2 — Source Data for Appendix [file EMBJ-37-e98044-s011.zip › 180518_Appendix_SourceData/180518_Table7_Fig8_FigS17a.docx]

**Table 7: Changes in the protein composition of synaptic vesicles as they age (relates to Fig 8 and Appendix Fig S17a).** In this set of experiments, we used two-color STED microscopy to investigate the association of young (0 days after live antibody tagging) and old (4 days after live antibody tagging) synaptic vesicles with various synaptic proteins. The goal of these experiments was to determine whether the molecular composition of synaptic vesicles is altered during their life cycle. We did not find any significant changes, apart from a 2-fold increase of SNAP25 association with old vesicles compared to young vesicles.

| Figure | Fig 8, Appendix Fig S17a |
| --- | --- |
| number of experiments | number of independent experiments per protein of interest (d0, [# of vesicles analyzed], d4 [# of vesicles analyzed]), >10 neurons sampled per experiment: SNAP25 (4 [36599], 3 [35419]), Syntaxin 1 (3 [17186], 3 [13231]), VGlut 1/2 (2 [20109], 3 [26268]), vATPase (3 [14506], 4 [38032]), VAMP2 (4 [29213], 4 [31892]), Synaptotagmin 1 (3 [23387], 3 [17523]), Syntaxin 16 (4 [32862], 4 [32199]), VAMP4 (3 [22538], 3 [26999]), Synapsin I/II (3 [15735], 3 [17656]). |
| statistics | Fig 8c: the unpaired t-test determined that the difference between day 0 and day 4 was significant for SNAP25, with p = 0.0124, t(5) = 3.8200. Unpaired t-tests found no significant differences between day 0 and day 4 for all other proteins of interest: for Syntaxin 1, p = 0.8850, t(4) = 0.1541; for VGlut 1/2, p = 0.1986, t(3) = 1.6447; for vATPase, p = 0.7340, t(5) = 0.3594; for VAMP2, p = 0.8837, t(6) = 0.1527; for Synaptotagmin 1, p = 0.1604, t(4) = 1.7208; for Syntaxin 16, p = 0.7406, t(6) = 0.3468; for VAMP4, p = 0.9863, t(4) = 0.0183; for Synapsin I/II, p = 0.6638, t(4) = 0.4685. |
| antibodies used | Synaptotagmin 1 live tagging: Synaptic Systems, 105 311AT, clone 604.2, lumenal domain, conjugated to Atto647N  immunostainings for proteins of interest, after fixation: SNAP25 (Synaptic Systems, 111 002), Syntaxin 1 (Synaptic Systems, 110 302), VGlut 1/2 (Synaptic Systems, 135 503), vATPase (Synaptic Systems, 109 002), VAMP2 (Synaptic Systems, 104 202), Synaptotagmin 1 (Synaptic Systems, 105 102), Syntaxin 16 (Synaptic Systems, 110 162), VAMP4 (Synaptic Systems, 136 002), Synapsin I/II (Synaptic Systems, 106 002) |
| antibody live tagging | Synaptotagmin 1 antibody was applied (1:120 from 1 mg/ml stock), to live primary hippocampal neurons, in their own culture medium, for 1 h at 37°C in a cell culture incubator. The antibody was then washed off with ice-cold Tyrode’s solution (3-times on/off), and the cultures were maintained in their own culture medium until processing for their respective time point. |
| description of time course | Synaptic vesicles in primary hippocampal cultures were tagged (as described in the previous table row). Directly after tagging, half of the cultures were processed immediately (day 0 time point, young synaptic vesicles); the other half were kept in culture for processing on day 4 after tagging (day 4 time point, aged synaptic vesicles); see two table rows below for details on processing. |
| stimulation paradigm | no external stimulation, only intrinsic network activity of primary hippocampal cultures during live antibody tagging and time course |
| fixation and processing | 4% PFA (15 min 4°C, 30 min on room temperature), standard immunostaining for proteins of interest (see above), melamine embedding and thin-sectioning at 20 nm per slice |
| imaging setup | Leica TCS SP5 STED (two-color STED mode), 100x apochromat oil immersion objective |
