# Supplementary material for: Newly produced synaptic vesicle proteins are preferentially used in synaptic transmission
Source: EMBO J. 2018 Jun 27;37(15):e98044. doi: 10.15252/embj.201798044 (PMC6068464; doi:10.15252/embj.201798044)
Supplement: Supplementary file 2 — Source Data for Appendix [file EMBJ-37-e98044-s011.zip › 180518_Appendix_SourceData/180518_Table8_Fig9_FigS18_FigS26.docx]

**Table 8: Expression of sypHy, sypHy-SNAP25, or sypHy-Syntaxin 1 to determine whether increased amounts of SNAP25 on a synaptic vesicle lead to its inactivation (relates to Fig 9, Appendix Fig S18, and Appendix Fig S26).** In this set of experiments, we expressed the lumenal pH-sensor sypHy on synaptic vesicles, as well as a sypHy construct coupled to SNAP25, and compared the response of both constructs to stimulation. In case of sypHy-SNAP25, release was severely depressed.

| Figure | Fig 9, Appendix Fig S18, Appendix Fig S26 |
| --- | --- |
| number of experiments | 9 independent experiments for sypHy, 9 independent experiments for sypHy-SNAP25, 12 independent experiments for sypHy-Syntaxin 1 |
| statistics | Fig 9e: one-way ANOVA indicated that significant differences were present in the data, with p = 0.0035, F(2, 31) = 6.90. Significant differences were found with the post-hoc Bonferroni procedure between the conditions “sypHy” and “sypHy-SNAP25” (p = 0.0160) as well as between the conditions “sypHy” and “sypHy-Syntaxin 1” (p = 0.0050). All other comparisons were not significant.  Appendix Fig S18b: one-way ANOVA determined that no significant differences were present in the data, with p = 0.6816, F(2, 17) = 0.39.  Appendix Fig S26e: one-way ANOVA determined that no significant differences were present in the data, with p = 0.0705, F(2, 30) = 2.92. |
| constructs used | sypHy (Synaptophysin coupled to pH-sensitive GFP-variant in an internal lumenal loop)  sypHy-SNAP25 (Synaptophysin coupled to pH-sensitive GFP-variant in an internal lumenal loop and SNAP25, mutated to remove all palmitoylation sites, on the cytoplasmic C-terminus)  sypHy-Syntaxin 1 (Synaptophysin coupled to pH-sensitive GFP-variant in an internal lumenal loop, and Syntaxin 1, without the membrane-integration domain and the intra-vesicular domain, on the cytoplasmic C-terminus) |
| description of time course | Neurons were transfected and maintained in culture for 3-4 days, until expression was sufficient for imaging. The neurons were then subjected to stimulation during imaging (see below) to detect differences in release between the two constructs. |
| stimulation paradigm | 600 action potentials (Fig 9) or 60 action potentials (Appendix Fig S26) delivered at 20 Hz in electrical field stimulation. |
| fixation and processing | no fixation, application of a pulse of 100 mM NH_4_Cl to achieve maximum fluorescence of all sypHy proteins in the neurons for normalization of the data acquired during the stimulation (Fig 9 and Appendix Fig S26) |
| imaging setup | Nikon Ti-E, 60x apochromat oil immersion objective; heating chamber to maintain neurons at 37°C during imaging |
