# Supplementary material for: Newly produced synaptic vesicle proteins are preferentially used in synaptic transmission
Source: EMBO J. 2018 Jun 27;37(15):e98044. doi: 10.15252/embj.201798044 (PMC6068464; doi:10.15252/embj.201798044)
Supplement: Supplementary file 4 — Source Data for Figure 1 [file EMBJ-37-e98044-s002.docx]

**Table 1: Basic time course of synaptic vesicle protein degradation (relates to Fig 1).** In this set of experiments we determined the degradation times of synaptic vesicles, tagged at the synapse during recycling, using antibodies directed against the lumenal domain of two synaptic vesicle proteins, Synaptotagmin 1 and VGAT. The antibodies were applied to live primary hippocampal neuron cultures. We determined that the half-life of degradation is ~2 days for both proteins. To ensure that synaptic signals were exclusively analyzed, only Synaptotagmin 1 or VGAT signals co-localizing with an immunostaining for Synaptophysin (an optimal presynaptic marker) were measured.

| Figure | Fig 1b-e |
| --- | --- |
| number of experiments | Synaptotagmin 1 live tagging: 3 (day 0), 3 (day 1), 2 (day 2), 3 (day 4), 3 (day 7), 2 (day 10) independent experiments, >10 neurons imaged per experiment  VGAT live tagging: 4 (day 0), 4 (day 1), 2 (day 2), 4 (day 4), 4 (day 7), 3 (day 10) independent experiments, >10 neurons imaged per experiment. |
| antibodies used | Synaptotagmin 1: Synaptic Systems, 105 311AT, clone 604.2, lumenal domain, conjugated to Atto647N  VGAT: Synaptic Systems, 131 103CpH, lumenal domain, conjugated to CypHer5E  co-immunostaining after fixation: Synaptic Systems, 101 004, Synaptophysin |
| antibody live tagging | Synaptotagmin 1 or VGAT antibody was applied (1:120 from 1 mg/ml stock), to live primary hippocampal neurons, in their own culture medium, for 1 h at 37°C in a cell culture incubator. The antibody was then washed off with ice-cold Tyrode’s solution (3-times on/off), and the cultures were maintained in their own culture medium until processing for their respective time point. |
| description of time course | Live tagging of releasing synaptic vesicles was performed as described in the previous table row, right before processing for the initial time point (day 0). Separate cultures for each time point (day 0, day 1, day 2, day 4, day 7, day 10) were pulsed in parallel and were maintained in incubator until processing. |
| stimulation paradigm | No external stimulation, only intrinsic network activity of primary hippocampal cultures during live antibody tagging and time course |
| fixation and processing | Synaptotagmin 1: 4% PFA (15 min 4°C, 30 min on room temperature), standard immunostaining for Synaptophysin to detect synapses, embedded in Mowiol  VGAT: methanol (20 min, -20°C), no additional immunostaining, application of pH 5.5 TES buffered solution to activate CypHer5E during imaging |
| imaging setup | Synaptotagmin 1: Leica TCS SP5 (confocal mode), 63x apochromat oil immersion objective  VGAT: Nikon Ti-E, 60x apochromat oil immersion objective |
