# Supplementary material for: Newly produced synaptic vesicle proteins are preferentially used in synaptic transmission
Source: EMBO J. 2018 Jun 27;37(15):e98044. doi: 10.15252/embj.201798044 (PMC6068464; doi:10.15252/embj.201798044)
Supplement: Supplementary file 5 — Source Data for Figure 2 [file EMBJ-37-e98044-s003.docx]

**Table 2: Releasability of ageing synaptic vesicle proteins in response to stimulation (relates to Fig 2).** In this set of experiments, we determined the fraction of synaptic vesicle molecules tagged with antibody (as described in Table 1) that are still able to exocytose in response to an external stimulus designed to release the entire recycling pool (600 action potentials). Right after tagging, almost all vesicle molecules are releasable. The releasable fraction steadily declines until almost none of the remaining tagged molecules are releasable 7-10 days later.

| Figure | Fig 2b-e |
| --- | --- |
| number of experiments | Synaptotagmin 1: 4 (day 0), 3 (day 1), 4 (day 2), 4 (day 4), 2 (day 7), 2 (day 10) independent experiments, >10 neurons imaged per experiment  VGAT: 4 (day 0), 4 (day 1), 2 (day 2), 4 (day 4), 4 (day 7), 3 (day 10) independent experiments, >10 neurons imaged per experiment |
| antibodies used | Synaptotagmin 1: Synaptic Systems, 105 311CpH, clone 604.2, lumenal domain, conjugated to CypHer5E  VGAT: Synaptic Systems, 131 103CpH, lumenal domain, conjugated to CypHer5E |
| antibody live tagging | Synaptotagmin 1 or VGAT antibody was applied (1:120 from 1 mg/ml stock), to live primary hippocampal neurons, in their own culture medium, for 1 h at 37°C in a cell culture incubator. The antibody was then washed off with ice-cold Tyrode’s solution (3-times on/off), and the cultures were maintained in their own culture medium until processing for their respective time point. |
| description of time course | Live tagging of releasing synaptic vesicles was performed (as described in the previous table row), right before processing for the initial time point (day 0). Separate cultures for each time point (day 0, day 1, day 2, day 4, day 7, day 10) were pulsed in parallel and maintained in the incubator until processing. On the respective time points, cultures were imaged live during stimulation with 600 action potentials (see next table row) before fixation and imaging in low- and high-pH buffers for normalization (see two table rows below). |
| stimulation paradigm | during live antibody tagging and time course : no external stimulation, only intrinsic network activity of primary hippocampal cultures  during experiment, to test releasability of vesicles: 600 action potentials delivered at 20 Hz in electrical field stimulation (in Tyrode’s solution with bafilomycin, to prevent re-acidification during imaging) |
| fixation and processing | Synaptotagmin 1 and VGAT: fixation in methanol (20 min, -20°C) after live imaging (with stimulation), no additional immunostaining. To determine the entire amount of antibodies present in the preparation, an application of pH 5.5 TES buffered salt solution was performed to activate CypHer5E. To determine the background fluorescence, not due to CypHer5E antibodies, an application of pH 7.4 PBS, to quench all CypHer5E molecules, was performed. |
| imaging setup | Synaptotagmin 1: Olympus, 60x apochromat oil immersion objective  VGAT: Nikon Ti-E, 60x apochromat oil immersion objective; heating chamber to maintain neurons at 37°C during imaging |
