# Supplementary material for: Newly produced synaptic vesicle proteins are preferentially used in synaptic transmission
Source: EMBO J. 2018 Jun 27;37(15):e98044. doi: 10.15252/embj.201798044 (PMC6068464; doi:10.15252/embj.201798044)
Supplement: Supplementary file 6 — Source Data for Figure 3 [file EMBJ-37-e98044-s004.docx]

**Table 3: Co-localization of actively recycling synaptic vesicles and aged synaptic vesicles with metabolic markers for recently produced proteins (relates to Fig 3 and Fig 4).** In this set of experiments, we tagged recycling synaptic vesicles (as described in Table 1), and determined the co-localization of the fluorophore-conjugated antibody with markers for recently produced proteins. We used two approaches to reveal recently produced proteins: FUNCAT, in which the unnatural amino acid AHA is incorporated into proteins during biogenesis, and can be detected after fluorophore-conjugation, and COIN, in which heavy ^15^N leucine is incorporated into proteins during biogenesis, and can be detected with a nanoSIMS mass spectrometry imaging device. The analysis shows that actively recycling synaptic vesicles have a significantly stronger co-localization with recently produced proteins.

| Figure | Fig 3c-e (FUNCAT), Fig 4 (COIN) |
| --- | --- |
| number of experiments | FUNCAT (Fig 3c-e): 3 (actively recycling vesicles, day 0), 3 (inactive vesicles, day 4) independent experiments, >10 neurons sampled per experiment  COIN (Fig 4): 57 synapses from 3 independent experiments (actively recycling vesicles, day 0), 47 synapses from 2 independent experiments (inactive vesicles, day 4) |
| statistics | FUNCAT (Fig 3e): the unpaired t-test determined a significant difference, with p = 0.0037, t(4) = 6.09.  COIN (Fig 4b): the unpaired t-tests determined that the difference between releasable and inactive vesicles was significant, with p = 0.0001, t(102) = 5.5378. Unpaired t-tests further determined that the difference between releasable vesicles and the axon was significant, with p = 0.0004, t(116) = 3.6156, and that the difference between inactive vesicles and the axon was significant, with p = 0.0001, t(96) = 4.0691 (see figure legend for details). |
| antibodies used | live antibody tagging, FUNCAT and COIN: lumenal domain of Synaptotagmin 1, Synaptic Systems, 105 311AT, clone 604.2, against, conjugated to Atto647N  co-immunostaining after fixation, COIN: Synaptophysin, Synaptic Systems, 101 004 |
| antibody live tagging | Synaptotagmin 1 or VGAT antibody was applied (1:120 from 1 mg/ml stock), to live primary hippocampal neurons, in their own culture medium, for 1 h at 37°C in a cell culture incubator. The antibody was then washed off with ice-cold Tyrode’s solution (3-times on/off), and the cultures were maintained in their own culture medium until processing for their respective time point. |
| metabolic labelling | FUNCAT: AHA was fed to the cultured neurons (in culture medium free of methionine, which is the amino acid AHA competes with for integration into proteins during biogenesis) for 9 h prior to processing the samples for the respective time points (see below).  COIN: ^15^N leucine was fed to the cultured neurons (in 3-fold molar excess over ^14^N leucine in the culture medium) for 1-3 days prior to processing the samples for the respective time points (see below). The extended feeding time compared to AHA in FUNCAT was necessary to reliably obtain a signal in nanoSIMS imaging. |
| description of time course | For actively recycling vesicles (d0 time point, directly after antibody tagging): AHA was fed to the cultured neurons for 9 h (see table row above), ^15^N leucine was fed to the cultured neurons for 1-3 days (see table row above); this metabolic labelling was immediately followed by live antibody tagging of actively recycling synaptic vesicles (see two table rows above).  For inactive vesicles (day 4 time point after antibody tagging): live antibody tagging of actively recycling synaptic vesicles in cultured neurons was performed (see two table rows above), and the neurons were maintained in culture for 4 days until processing. During the last 9 hours of this 4 day interval, AHA was fed to the cultured neurons. Alternatively, during the last 24-72 hours of this 4 day interval, ^15^N leucine was fed to the cultured neurons. |
| stimulation paradigm | no external stimulation, only intrinsic network activity of primary hippocampal cultures during live antibody tagging and time course |
| fixation and processing | FUNCAT: 4% PFA (15 min 4°C, 30 min on room temperature), standard immunostaining for Synaptophysin to detect synapses, melamine embedding and thin-sectioning at 20 nm per slice  COIN: 4% PFA (15 min 4°C, 30 min on room temperature), standard immunostaining for Synaptophysin and PSD95 to detect synapses, LR-White embedding and thin-sectioning at 200 nm per slice; melamine embedding is unsuitable for nanoSIMS imaging, as the amines in the resin mask the mass spectrometry signal from the sample in the nitrogen channel |
| imaging setup | FUNCAT: Leica TCS SP5 STED (two-color STED mode), 100x apochromat oil immersion objective  COIN, fluorescence imaging: Nikon Ti-E, 100x apochromat oil immersion objective, additional 1.5x magnification lens  COIN, ^15^N leucine mass spectrometry imaging: Cameca nanoSIMS 50L |
