# Supplementary material for: Newly produced synaptic vesicle proteins are preferentially used in synaptic transmission
Source: EMBO J. 2018 Jun 27;37(15):e98044. doi: 10.15252/embj.201798044 (PMC6068464; doi:10.15252/embj.201798044)
Supplement: Supplementary file 8 — Source Data for Figure 5 [file EMBJ-37-e98044-s006.zip › SourceData_Fig5def.docx]

**Table 5: Controls for synaptic activity during drug treatments described in Table 4 (relates to Fig 5d-f).** As the drug treatments described above (Table 4, Fig 5a-c) might by themselves alter synaptic activity, irrespective of any effect arising solely from the cut-off of synapses from newly produced vesicles, we performed several controls addressing the following physiological parameters of untreated and drugged cultures: fraction of actively recycling synaptic vesicles in response to stimulation, fraction of actively recycling synaptic vesicles during intrinsic network activity, and size of the total pool of synaptic vesicles per synapse. We found that none of these parameters were significantly altered by the drug treatments we employed here.

| Figure | Fig 5d (fraction of actively recycling synaptic vesicles in response to stimulation), Fig 5e (fraction of actively recycling synaptic vesicles during intrinsic network activity), Fig 5f (size of the total pool of synaptic vesicles per synapse). |
| --- | --- |
| number of experiments | number of independent experiments per condition: 3 (all data points) |
| statistics | Fig 5d: one-way ANOVA determined that no significant differences were present in the data, with p = 0.2205, F(2, 8) = 1.97.  Fig 5e: one-way ANOVA determined that no significant differences were present in the data, with p = 0.8835, F(2, 8) = 0.13.  Fig 5f: one-way ANOVA determined that no significant differences were present in the data, with p = 0.5519, F(2, 8) = 0.66. |
| antibodies used | Synaptotagmin 1: Synaptic Systems, 105 311AT, clone 604.2, lumenal domain, conjugated to Atto647N |
| antibody live tagging | Synaptotagmin 1 antibody was applied (1:120 from 1 mg/ml stock), to live primary hippocampal neurons, in their own culture medium, for 1 h at 37°C in a cell culture incubator. The antibody was then washed off with ice-cold Tyrode’s solution (3-times on/off), and the cultures were maintained in their own culture medium until processing for their respective time point. Alternatively, for live antibody tagging during stimulation (Fig 5d), the same antibody was applied during stimulation in Tyrode’s solution at room temperature, followed by a resting period of 5 min to allow synaptic vesicle recycling, followed by fixation. |
| drug application | anisomycin (40 µM) to inhibit protein biogenesis, or colchicine (10 µM) to disrupt vesicle transport along the microtubule network |
| description of time course | Neurons were treated with drugs (as described in Table 4) or left untreated for 24 h (the longest time point in time courses of experiments described in Table 4, Fig 5b,c). Then antibodies for live tagging were applied either during intrinsic network activity (Fig 5e) or during electrical field stimulation (Fig 5d). The amount of synaptic vesicles (Fig 5f) was determined from the samples used in Fig 5d, using a post-fixation immunostaining for Synaptophysin (>95% localization to synaptic vesicles). |
| stimulation paradigm | for 5e: no external stimulation, only intrinsic network activity of primary hippocampal cultures during live antibody tagging  for 5d: 600 action potentials delivered at 20 Hz in electrical field stimulation during antibody live tagging |
| fixation and processing | 4% PFA (15 min 4°C, 30 min on room temperature), standard immunostaining for Synaptophysin to detect synapses, embedded in Mowiol |
| imaging setup | Leica TCS SP5 (confocal mode), 63x apochromat oil immersion objective |
