# Supplementary material for: Newly produced synaptic vesicle proteins are preferentially used in synaptic transmission
Source: EMBO J. 2018 Jun 27;37(15):e98044. doi: 10.15252/embj.201798044 (PMC6068464; doi:10.15252/embj.201798044)
Supplement: Supplementary file 8 — Source Data for Figure 5 [file EMBJ-37-e98044-s006.zip › SourceData_Fig5bc.docx]

**Table 4: Blocking Synaptotagmin 1 epitopes on actively recycling synaptic vesicles to observe the influx of unused epitopes from biogenesis or from the reserve pool (relates to Fig 5b,c).** In this set of experiments, we used monoclonal antibodies not conjugated to any fluorophores to block access to actively recycling epitopes for clonally identical fluorophore-conjugated antibodies applied afterwards. This allowed us to monitor the influx of new, recently unused (and thus unblocked) epitopes into the recycling pool of synaptic vesicles. We applied drugs that prevent the production or transport of new synaptic vesicles to dissect whether the new, not recently used (and thus unblocked) epitopes enter the recycling pool from biogenesis or from the reserve pool. We found that the reserve pool alone is unable to provide synaptic vesicles for release under physiological stimulation conditions, and that they come from biogenesis.

| Figure | Fig 5b,c |
| --- | --- |
| number of experiments | 4 independent experiments for time point 0 h of the untreated condition, 3 independent experiments for all other data points, >10 neurons sampled per experiment |
| statistics | Fig 5c: one-way ANOVA indicated that significant differences were present in the data, with p = 0.2205, F(2, 8) = 1.97. Significant differences were found with the post-hoc Bonferroni procedure between the conditions “untreated” and “anisomycin” (p = 0.0070) as well as between the conditions “untreated” and “colchicine” (p = 0.0022). All other comparisons were not significant. |
| antibodies used | for blocking epitopes of recently exocytosed Synaptotagmin 1: Synaptic Systems, 105 311, clone 604.2, lumenal domain, unconjugated  for detecting new, unblocked Synaptotagmin 1: Synaptic Systems, 105 311AT, clone 604.2, lumenal domain, conjugated to Atto647N |
| antibody live tagging | For blocking: unconjugated Synaptotagmin 1 antibody was applied (1:120 from 1 mg/ml stock), to live primary hippocampal neurons, in their own culture medium, for 2 h at 37°C in the cell culture incubator. The antibody was then washed off with ice-cold Tyrode’s solution (3-times on/off), and the cultures were maintained in their own culture medium until processing for their respective time point.  For detection: conjugated Synaptotagmin 1 antibody was applied (1:120 from 1 mg/ml stock), to live primary hippocampal neurons, in their own culture medium, for 30 min at 37°C in the cell culture incubator. The antibody was then washed off with ice-cold Tyrode’s solution (3-times on/off), and the cultures were maintained in their own culture medium until processing for their respective time point. |
| drug application | anisomycin (40 µM) to inhibit protein biogenesis, or colchicine (10 µM) to disrupt vesicle transport along the microtubule network |
| description of time course | The unconjugated antibody was applied for blocking Synaptotagmin 1 epitopes on actively recycling synaptic vesicles to all samples for all time points in parallel, 2 h before the first 30 min pulse of fluorophore-conjugated antibody. The fluorophore-conjugated antibody was then applied at the respective time points, and the samples were fixed and processed (see two table rows below). |
| stimulation paradigm | no external stimulation, only intrinsic network activity of primary hippocampal cultures during live antibody blocking and tagging for detection during the time course. |
| fixation and processing | 4% PFA (15 min 4°C, 30 min on room temperature), standard immunostaining for Synaptophysin to detect synapses, embedded in Mowiol |
| imaging setup | Leica TCS SP5 (confocal mode), 63x apochromat oil immersion objective |
